# Flower-fruit dynamics, visitor-predator patterns and chemical preferences in the tropical bamboo, *Melocanna baccifera*

**DOI:** 10.1371/journal.pone.0277341

**Published:** 2022-11-16

**Authors:** Konnath Chacko Koshy, Bhaskaran Gopakumar, Antony Sebastian, Ajikumaran Nair S., Anil John Johnson, Balaji Govindan, Sabulal Baby

**Affiliations:** 1 Plant Genetic Resources Division, Jawaharlal Nehru Tropical Botanic Garden and Research Institute, Thiruvananthapuram, Kerala, India; 2 Phytochemistry and Phytopharmacology Division, Jawaharlal Nehru Tropical Botanic Garden and Research Institute, Thiruvananthapuram, Kerala, India; University of Southampton, UNITED KINGDOM

## Abstract

Mast seeding and associated events in *Melocanna baccifera*, the largest fruit producing bamboo, is an enigma. So far there are no comprehensive accounts on its flowering phenology, fruiting dynamics and animal interactions. In this study, spanning over 13 years (2009 to 2022), we observed eight *M*. *baccifera* clumps in JNTBGRI Bambusetum from flowering initiation, fruiting to eventual death. Flowering phenology, floral characteristics, floret types, breeding system, bee visitation, pollination, fruit production and predators were recorded; predation patterns were correlated with fruit chemistry. Flowering duration of clumps ranged from 20 (March 2009—October 2010) to 120 (September 2012—August 2022) months. Bisexual florets are dichogamous and protogynous; and female duration (22–72 h) is many times higher than male duration (2–6 h). The highest ever fruit production for an individual bamboo clump (456.67 Kg) was recorded. Of the total fallen fruits (38371), 38.11% were predated, 43.80% good fruits (no predator hits) and 18.09% immature fruits. A positive correlation between reward (fruits) *versus* predation was observed, especially in short intervals of high fruit production. Pollen predators (*Apis cerana indica*, *Halictus taprabonae*, *Braunsapis cupulifera*, *Trigona iridipennis*), fruit predators, ranging from arthropods to mammals, *viz*., millipede (*Spinotarsus colosseus)*, slug (*Mariaella dussumieri*), snails (*Cryptozona bistrialis*, *Macrochlamys* sp.), borers (*Achroia grisella*, *Blattella germanica*), mammals (monkeys *Macaca radiata*, rats *Rattus rattus*, porcupine *Hystrix indica*, wild boar *Sus scrofa*, palm civet *Paradoxurus hermaphroditus*), seedling predators (rabbit *Lepus nigricollis*, deer *Axis axis*), and insect/pest predators (ants *Crematogaster biroi*, *Oecophylla smaragdina*, mantis *Euchomenella indica*) were identified. Fruit predation is linked to its age and chemistry. Apart from new insights on flowering phenology, breeding system, pollination and fruiting dynamics, this study demonstrates the vibrant interaction between *M*. *baccifera* flowers/fruits and visitors/predators, and provides significant leads towards elucidating the cause of rat multiplication and other events associated with its gregarious flowering.

## Introduction

*Melocanna baccifera* (Roxb.) Kurz (family Poaceae) is a unique bamboo characterized by open diffuse clumps (due to sympodial rhizomes with c. one m long neck) and baccate fleshy fruits (largest in the grass family). It is natively distributed in the Indian subcontinent to Myanmar and naturalised in Jamaica, Colombia, Ecuador and Southeast Brazil [1]. This monocarpic species flowers gregariously and its flowering cycle ranges from 40 to 50 years [2–5]. However, a recent study has set it precisely at 48 years [6]. In northeast India *M*. *baccifera* is locally known as ‘*Muli*’, and it carries huge economic impact in every sector of life such as food, traditional medicine, house construction and paper industries. The local hill tribes relish the sweet fluid in its young fruits and use its white embryo as medicine [2]. During gregarious flowering, *Muli* produces enormous fruits which entice various visitors to them [7–11]. Of the visitors, rats heavily predate on the fruits and, due to their short reproductive cycles, multiply at a faster rate. Subsequently, when the fruit production dwindles rats attack every other standing crop ensuing an ecological havoc. Such episodes culminated in famines of 1881 (c. 15,000 people died), 1912 and 1959 (10,000–15,000 people died) [12, 13]. Gregarious flowering also results in colossal loss of biomass. *M*. *baccifera* flowering during 2004–2009 in northeast India, spread over 1.76 million hectares, culminated in the death of about 26 million tonnes of bamboo culms [11, 12]. The Indian state of Mizoram has a high density of *Muli*, and is considered as the geographic center of this natural phenomenon [14, 15]. The combination of incidents associated with *Muli* flowering is locally known as ‘*Mautam*’ or ‘*bamboo death*’ [2, 7–11, 16, 17]. These ecological events have even led to political uprisings in the past [8, 18].

Reports (popular views) often highlighted the ‘high-protein fruits/seeds’ as the reason for elevated levels of predation on this bamboo and subsequent rat multiplication [2, 7, 11, 15, 19, 20]. *Muli* fruits are also considered as an aphrodisiac [2, 15, 20]. The high protein content and aphrodisiac credentials remained as untested views until our group reported its fruit chemistry and nutritional status, wherein we found only very little protein [9, 21]. We hypothesize that the breeding system in *M*. *baccifera* has evolved to produce a glut of fruits with chemical constituents capable of enticing various predators. We present an ecological (Bambusetum-laboratory) study on the (i) flowering phenology and fruiting dynamics of *M*. *baccifera*, (ii) its visitors and predators, (iii) interactions between its flowers, fruits and visitors/predators and (iv) chemical preferences driving these plant-animal interactions.

## Materials and methods

### Study area, *M*. *baccifera* clumps

We conducted this study in the Bambusetum of Jawaharlal Nehru Tropical Botanic Garden and Research Institute (JNTBGRI) (N 08˚ 45.262–430’ E 77˚ 01.429–583’) wherein *M*. *baccifera* from various locations in north east and north west India was introduced from 1988 to 1996. JNTBGRI Bambusetum holds 11 *M*. *baccifera* clumps bearing accession numbers 58, 359, 365, 366, 392, 393, 394, 395, 403, 404 and 405. This study of 13 years 7 months (from February 2009 to August 2022) was centered on eight flowering clumps (58, 359, 365, 394, 395, 403, 404, 405) which are located between 8° 45.295’ and 8° 45.351’ north latitudes and 77° 01.491’ and 77° 01.580’ east longitudes [22, 23]. The propagules of referred clumps were collected from Uttarakhand and Manipur states, India through field explorations (S1 Table in S1 File), and their passport data are well documented [22, 23]. The altitudes of their original habitats vary from 435 m (clump 58, collected from Dehra Dun, Uttarakhand) to 1061 m (clump 365, Saikul Hills, Manipur) above MSL. In comparison, the altitude of JNTBGRI Bambusetum is in the range 79–186.6 m above MSL [22, 23].

### Flowering phenology, visitors

The selected clumps were observed regularly from the sign of initiation of flowering to death. A large number of young spikelets, at a very early stage of development, were spotted with marker pen and morphological changes of florets were recorded daily till maturity of fruits. The flowering culms and florets were selected by random sampling. Florets on main culm nodes, borne on branches of lower half and upper half of the culms, were noted separately, classified and counted. The time of opening of florets, duration of stigma exposure, exsertion and maturation of stamens to dehiscence were noted. For recording the duration of male and female phases, 10 to 15 about-to-open florets were observed daily; their stigma exposure, curling and drying times were documented. Observations on bee visitation were carried out from 6.00 am to 6.00 pm, noting ambient temperatures and relative humidities concurrently.

### Fruit age studies

Observations were carried out in bisexual florets. As they are protogynous (carpels mature prior to stamens) the full exposure of stigma was considered as the starting point of fruit development. Each healthy pistil was dotted with a permanent marker, dates were marked on aluminium labels and tagged to the branch. Several pistils thus marked in eight clumps were observed, and their progressive development till maturity was recorded at weekly intervals. Fruits at the age of 7, 14, 21, 28, 35 and 42 days were plucked and their length-weight measurements were taken (S1 Fig in S1 File). They were cut open, volume and specific gravity of liquid contents in young fruits and seed-weight in mature ones were recorded.

### Fruit dynamics, fruit fall, predators

Fruits shed by eight clumps were gathered daily; the mature ones without any animal bites or other injuries were scored as *Good fruits* (GF), underdeveloped ones as *Immature fruits* (IF) and animal bitten ones as *Predated fruits* (PF). Daily fruit counts were added to get the month’s total (S3-S7 Tables in S1 File). To study the nature and prevalence of borer attack on fruits, a survey was conducted on clump 58 from January to December 2011. Fruits at eye level (c.150 cm from ground) and below were observed daily and those with signs of borer bout (i.e., borer holes, excreta deposited over the fruit surface) were scored. The presence of borers was confirmed by dissecting the fruits. Similarly, snails and slugs on young and mature fruits borne at and below eye levels on clumps 58 and 359 were observed daily from August 2010 to August 2011 and May 2009 to December 2011, respectively. Fruits with slugs or snails predating on them and those fruits with characteristic depressions and cavities were recorded. To record mammal predation, clump 359 was studied in detail from May 2009 to December 2011. We searched for hoof marks and peculiar soil rooting characteristics beneath fruiting clumps to confirm visits of wild boars [24] and quills of porcupines to corroborate their visits and predation. Rats were captured live using Sherman’s traps and identified. In late evenings, burrows in the area were examined with the help of search lights for palm civet. Monkey visits were also observed. Bitten fruits and left over remains were gathered and organisms predating on them were noted. All insects, borer larvae and slug/snails were collected and preserved in 70% ethyl alcohol and identified later. All visitors/predators and predation events were photographed (S2-S18 Figs in S1 File); voucher specimens, spirit collections and photographs were deposited at TBGT (S2 Table in S1 File).

### Fruit chemistry, rat feeding preferences

Secondary metabolites of *M*. *baccifera* fruits and leaves, sugar-amino acid-protein profiles and other nutritional parameters were elucidated during the span of this study [9, 21]. Rat feeding preferences of fruit pericarp, fruit liquid, seed and sugars (Glucose (Glu), Fructose (Fru), Sucrose (Suc)) were determined. Serum hematological and biochemical parameters of rats subjected to *M*. *baccifera* fruit+normal food, fruit alone and normal food feeding experiments were also analyzed [9]. An earlier study has confirmed that bamboo flowers have no influence on the reproductive activity in mice [25].

## Results

### Flowering phenology

Of the eight *M*. *baccifera* clumps, four (58, 359, 394, 395) started flowering in 2009, one (403) in 2011, two (365, 404) in 2012 and one (405) in 2013 (S4 Table in S1 File). Seven clumps began flowering during January to April, [January (365), February (359, 394, 405), March (395, 403), April (58)], prior to the southwest monsoon which generally begins in early June and weakens by September. One clump (404) initiated flowering in September (S4 Table in S1 File). Duration of flowering (initiation to cessation) ranged between 1 year 8 months (395) and 10+ years (404, as on August 2022, flowering continues). Clumps in open areas and steep hilly slopes (395, 394) displayed shorter (20–21 months) flowering durations, while those under deep shade showed longer (404: 120 months) flowering periods (S1, S4 Tables in S1 File). Watering during summer months enhanced the longevity of the clumps. The propagules of clumps 403 and 404 were collected from the same location, but they exhibited marked variation in their flowering periods (403: 4 years 4 months, 404: 10+ years, live) (S1, S4 Tables in S1 File).

**Fig 1:**
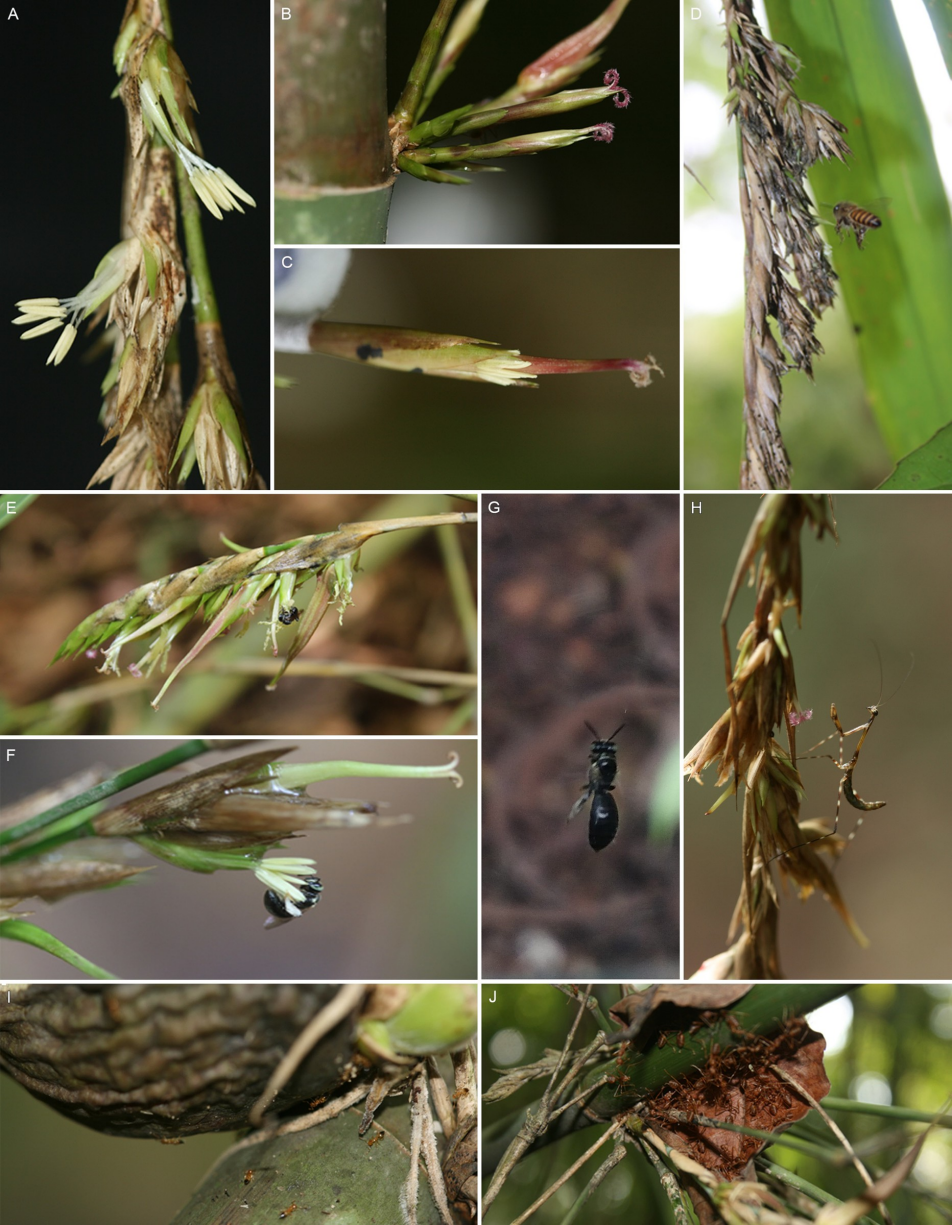
*M*. *baccifera* florets, visitors. A-C. Three types of florets, A. Male florets, B. Female florets, C. Bisexual florets showing protogyny (displaying drying stigma and emerging anthers); D-H Insect visits, D. *A*. *cerana indica*, E. *T*. *iridipennis*, F. *H*. *taprabonae*, G. *B*. *cupulifera*, H. Mantis *E*. *indica*; I-J ants, I. *C*. *biroi*, J. *O*. *smaragdina*.

Each spikelet contains 3–8 florets of which only one or two are fertile (Fig 1). Many of the marked florets were lost at various growth stages due to drying, infections, stunted growth and premature falling. There are three types of florets: staminate, pistilate and bisexual (Fig 1). In branches on the upper half of culms, approximately 70% florets were males, 5% males with rudimentary pistil and 25% bisexual florets. In lower half branches, approximately 85% florets were bisexual, 6% females with rudimentary stamens, 3% females (with pistil only) and 6% males (without pistil). The culm nodes had the same composition of florets as the lower branches (85: 6: 3: 6). Time taken for full emergence of stigma (S8 Table in S1 File) is 151.89 ± 22.99 min [2 h 32 min, n = 37; lowest 112 min (1 h 52 min), highest 194 min (3 h 14 min)] and duration of active stigma (receptivity) is 1790.54 ± 657.75 min [29 h 51 min, n = 37; lowest 1316 min (21 h 56 min), highest 4298 min (71 h 38 min)]. Thus, the stigma is active for c. 22 to 72 h. Duration of male stage (S8 Table in S1 File) is 261.81 ± 58.58 min [4 h 22 min, n = 37, lowest 122 min (2 h 2 min), highest 355 min (5 h 55 min)]. The male stage is active only for 2 to 6 h. The female duration is approximately 7 times that of the male stage. The duration between female and male stage (S8 Table in S1 File) is 3304.32 ± 1012.02 min (55.07 h ±16.87 min), n = 37, lowest 1400 min (23 h 20 min), highest 5800 min (96 h 40 min)]. The gap is generally of two to three days and rarely one or four days.

### Visitors, predators

#### Floral visitors, predators, inhabitants: Bees, mantis, ants

**Fig 2:**
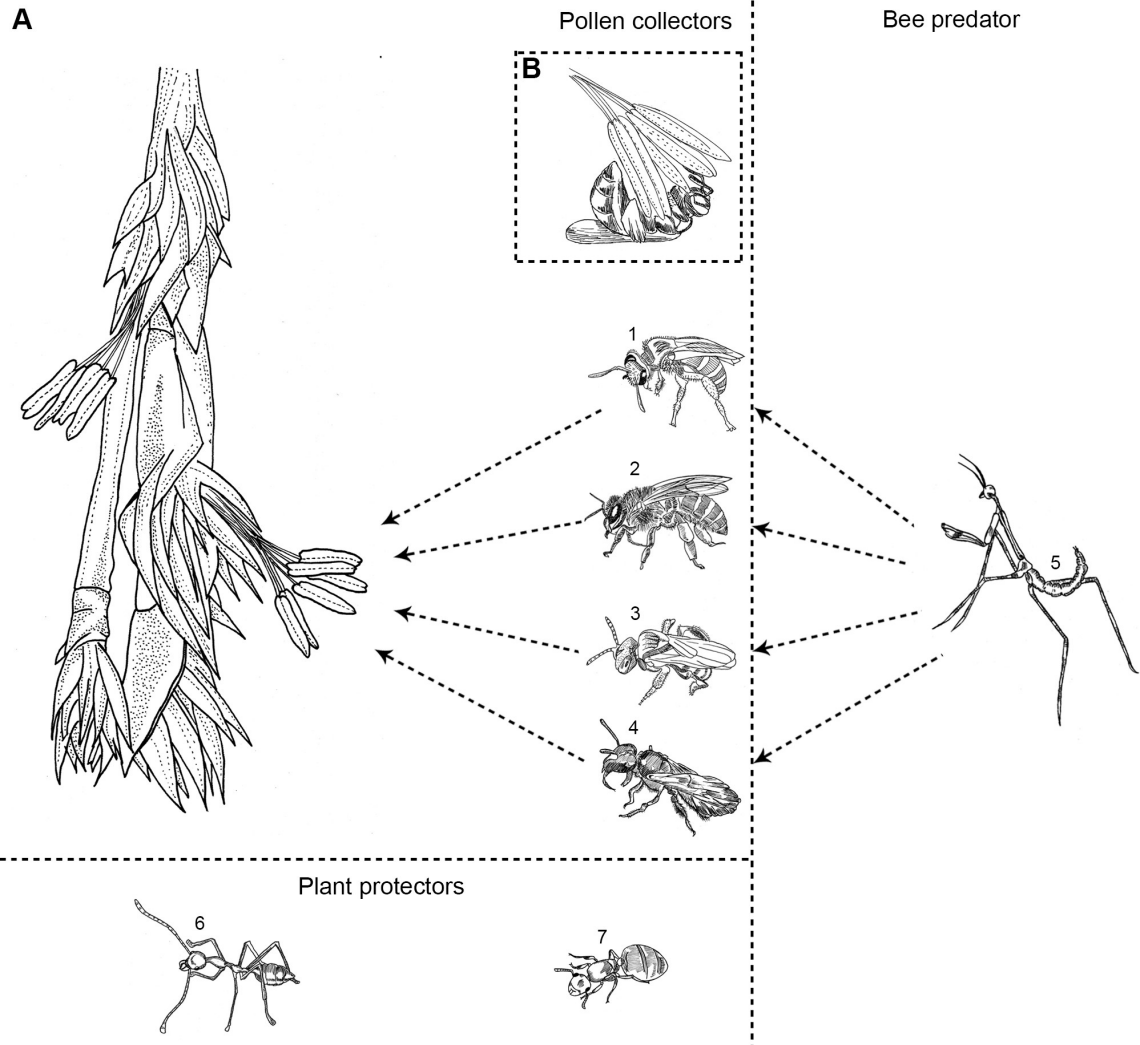
Diagrammatic sketch of pollen collectors, bee predators and plant protectors in *M*. *baccifera*. A. Part of inflorescence showing stamens, B. Bee visitation on stamens of a floret, 1–4 Pollen collectors, 1. *A*. *cerana indica*, 2. *H*. *taprabonae*, 3. *B*. *cupulifera*, 4. *T*. *iridipennis*; 5. Bee predator, Mantis *E*. *indica*; 6–7. Plant protectors, Ants, 6. *O*. *smaragdina*, 7. *C*. *biroi*.

*Bees*. Bee species *viz*., *Apis cerana indica*, *Trigona iridipennis* (both honey bees), *Halictus taprabonae* and *Braunsapis cupulifera*, visited the florets during the male stage (Figs 1 and 2 and S2(A), S2(B), S3, S4(A), S4(B) Figs in S1 File and Table 1). They landed on the anthers and broke the anther lobes by their mouth appendages and front legs to collect pollen grains. While collecting pollens, bees’ hind metasomal part throb the anther lobes and this beating accompanied by the fluttering of their wings produced a humming sound, audible even at a distance of 12–15 m from the clump, especially during *A*. *cerana* visitation. After visiting one floret, the bees visited other florets. Bee activity was never observed during the female stage and on the stigma. Bee visitation started by 6.30 am, continued usually up to 12 noon and sometimes up to 4.30 pm (temperature 23–29°C, relative humidity 47–68%). Intense bee activity was observed between 8 am and 12 noon (temperature 25–28°C, RH 50–65%).

**Table 1:** Floral visitors, fruit and seedling predators in *M*. *baccifera*.

**A. Floral visitors**															
Stage		Visiting locations			Collecting materials			Collectors/predators							
Male		Stamens/anthers			Pollen grains			**Bees** • *A*. *cerana indica* (honey bee) • *T*. *iridipennis* (honey bee) • *H*. *taprabonae* • *B*. *cupulifera*							
Male, female		Inflorescences			Bees			**Mantis** • *E*. *indica*							
Male, female and fruiting		Inflorescences, fruiting branches, branch complements, stem (inside & outside)			Insects and pests			**Ants** • *C*. *biroi* • *O*. *smaragdina*							
**B. Fruit-seedling predators**															
Fruit age (days) (weeks)	Fruit stage			Fruit liquid			Fruit pericarp			Predation on fruits attached to plant			Predation on fallen fruits		Major groups
				Glu-Fru-Suc (%)			Glu-Fru-Suc (%)			Common	Occasional		Common	Occasional	
**(1–7)** **(1)**	Liquid endosperm			No fruit liquid			(0.52%)-(0.51%)-(0.13%)			**Slug** (*M*. *dussumieri*) **Snail 02** (*C*. *bistrialis*)	**Snail 01** (*Macrochlamys* sp.)		-	**Slug** (*M*. *dussumieri*) **Snail 02** (*C*. *bistrialis*)	**Slugs/** **snails**
**(8–14) (2)**	Liquid endosperm			(0.16%)-(0.26%)-(0.00%)			(0.65%)-(0.83%)-(0.14%)			**Slug** (*M*. *dussumieri*) **Snail 02** (*C*. *bistrialis*) **Borer 02** (*A*. *grisella*)	**Snail 01** (*Macrochlamys* sp.) **Borer 01** (*B*. *germanica*)		**Slug** (*M*. *dussumieri*) **Snail 02** (*C*. *bistrialis*)	-	**Slugs/** **snails**
**(15–21) (3)**	Liquid to semi solid endosperm			(0.07%)-(0.10%)-(0.06%)			(0.35%)-(0.50%)-(0.08%)			**Snail 02** (*C*. *bistrialis*) **Borer 02** (*A*. *grisella*) **Monkeys** (*M*. *radiata*)	**Borer 01** (*B*. *germanica*)		**Wild boar**^**Ψ**^ (*S*. *scrofa*) **Slug** (*M*. *dussumieri*) **Snail 02** (*C*. *bistrialis*)	**Rabbits**^**¥**^ *L*. *nigricollis*	**Monkeys**
**(22–28) (4)**	Semi solid endosperm			(0.09%)-(0.09%)-(0.42%)			(0.51%)-(0.68%)-(0.14%)			**Borer 02** (*A*. *grisella*) **Monkeys** (*M*. *radiata*)	**Borer 01** (*B*. *germanica*)		**Wild boar**^**Ψ**^ (*S*. *scrofa*)	**Rabbits**^**¥**^ *L*. *nigricollis*	**Monkeys**
**(29–35) (5)**	Near mature			(0.08%)-(0.09%)-(0.48%)			(0.42%)-(0.60%)-(0.22%)			**Monkeys** (*M*. *radiata*) **Rats** (*R*. *rattus*) **Borer 02** (*A*. *grisella*)	**Borer** (*A*. *grisella*) **Borer 01** (*B*. *germanica*)		**Wild boar**^**Ψ**^ (*S*. *scrofa*) **Rats** (*R*. *rattus*)	**Porcupine** (*H*. *indica*) **Rabbits**^¥^ *L*. *nigricollis*	**Monkeys/** **rodents**
**(36–42) (6)**	Mature			(0.30%)-(0.42%)-(0.30%)			(0.10%)-(0.15%)-(0.31%)			**Monkeys** (*M*. *radiata*) **Rats** (*R*. *rattus*) **Borer 02** (*A*. *grisella*)	**Palm civet** (*P*. *hermaphroditus*) **Borer 01** (*B*. *germanica*)		**Wild boar**^**Ψ**^ (*S*. *scrofa*) **Rats** (*R*. *rattus*)	**Porcupine** (*H*. *indica*)	**Monkeys/** **rodents**
**(43–49) (7)**	Fully mature (initiation of germination)			-			-			**Rats** (*R*. *rattus*)	-		**Wild boar**^**Ψ**^ (*S*. *scrofa*) **Rats** (*R*. *rattus*)	**Porcupine** (*H*. *indica*)	**Rodents**
**C. Seedling predators**															
**(50-onwards)**			Seedlings						**Rabbits**^**¥**^ *L*. *nigricollis* **Deer**^**¥**^ *A*. *axis* **Wild boar**^**Ψ**^ (*S*. *scrofa*)						

^**¥**^Rabbits (*L*. *nigricollis*) and deers (*A*. *axis*) are primarily seedling predators, but occasionally they predate on fruits.

^**Ψ**^Wild boars (*S*. *scrofa*) predate mainly on fruits, but also consume seedlings (S14 Fig in S1 File).

*Mantis*, *ants*. Praying mantis, *Euchomenella indica* [26, 27], was found visiting inflorescences and predating on bees (Figs 1 and 2 and S5(A), S5(B) Fig in S1 File and Table 1). Among the two ant species observed, *Crematogaster biroi* colonized inside the hollow spaces of mature culms and nodes congested with mature or viviparous fruits, while *Oecophylla smaragdina* (weaver ant, green ant, green tree ant, orange gaster) nested on dried culm sheaths and terminal leaf twigs (Figs 1 and 2 and S6(A), S6(B), S7(A), S7(B) Figs in S1 File and Table 1). *Ochrophora montana* (cinnamon bug, brown bug, seed bug), a serious pest and a high value delicacy in Mizoram (locally ‘Thangnang’), associated with *M*. *baccifera* gregarious flowering [2, 28], was not observed in the study clumps.

#### Fruit predators

**Fig 3:**
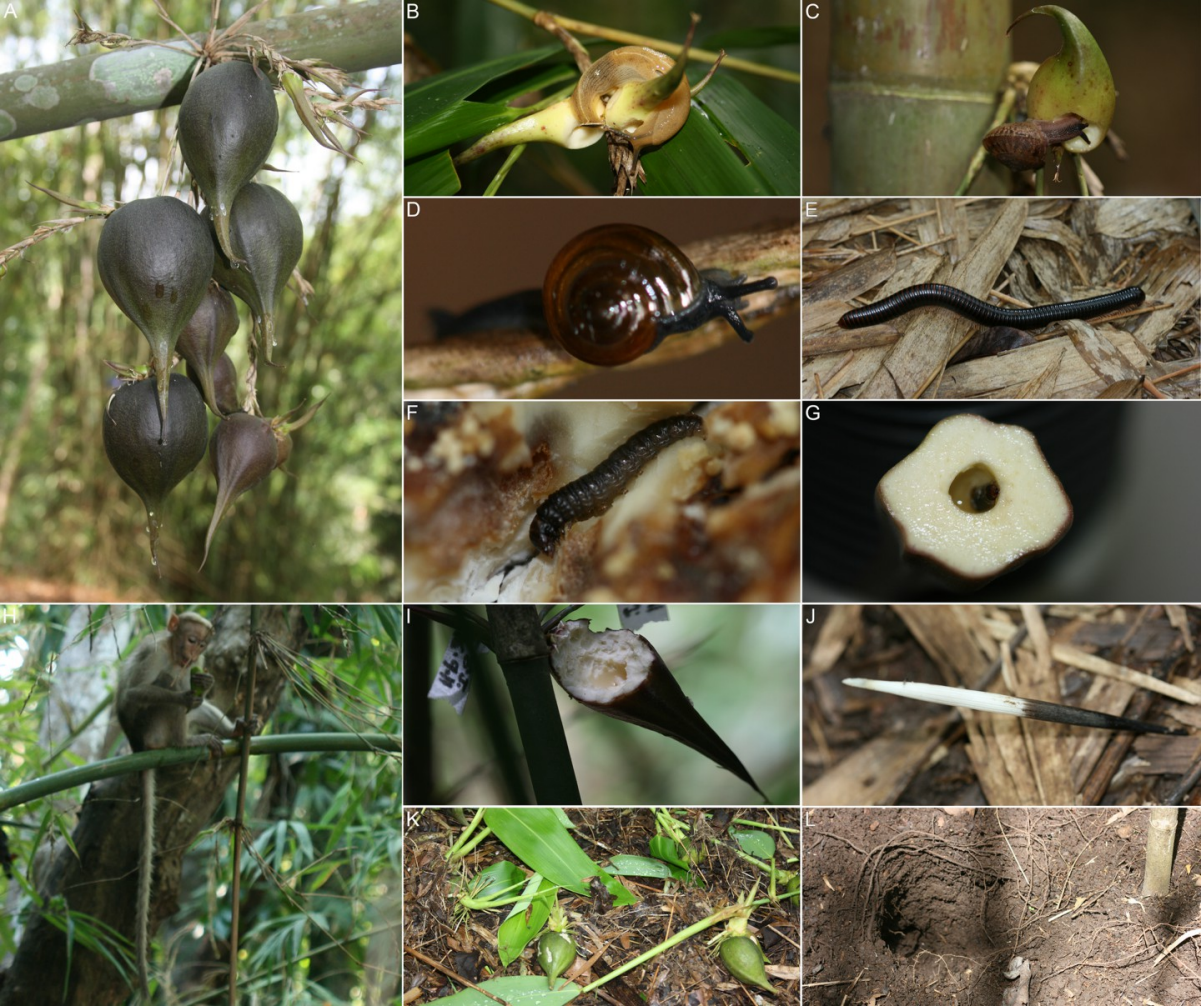
*M*. *baccifera* fruits, predators. A. Fruits, B-L fruit predators, B-D. Slugs and snails, B. *M*. *dussumieri*, C. *C*. *bistrialis*, D. *Macrochlamys* sp., E. Millipede *S*. *colosseus*, F-G Fruit borers, F. Larvae of *A*. *grisella*, G. Larvae of *B*. *germanica*, H-L Mammals, H. Bonnet macaque, *M*. *radiata*, I. Fruit bitten by rats *R*. *rattus*, J. Quill of porcupine *H*. *indica*, K. Seedlings spoilage by wild boars *S*. *scrofa*, L. Soil rooting by *S*. *scrofa*.

**Fig 4:**
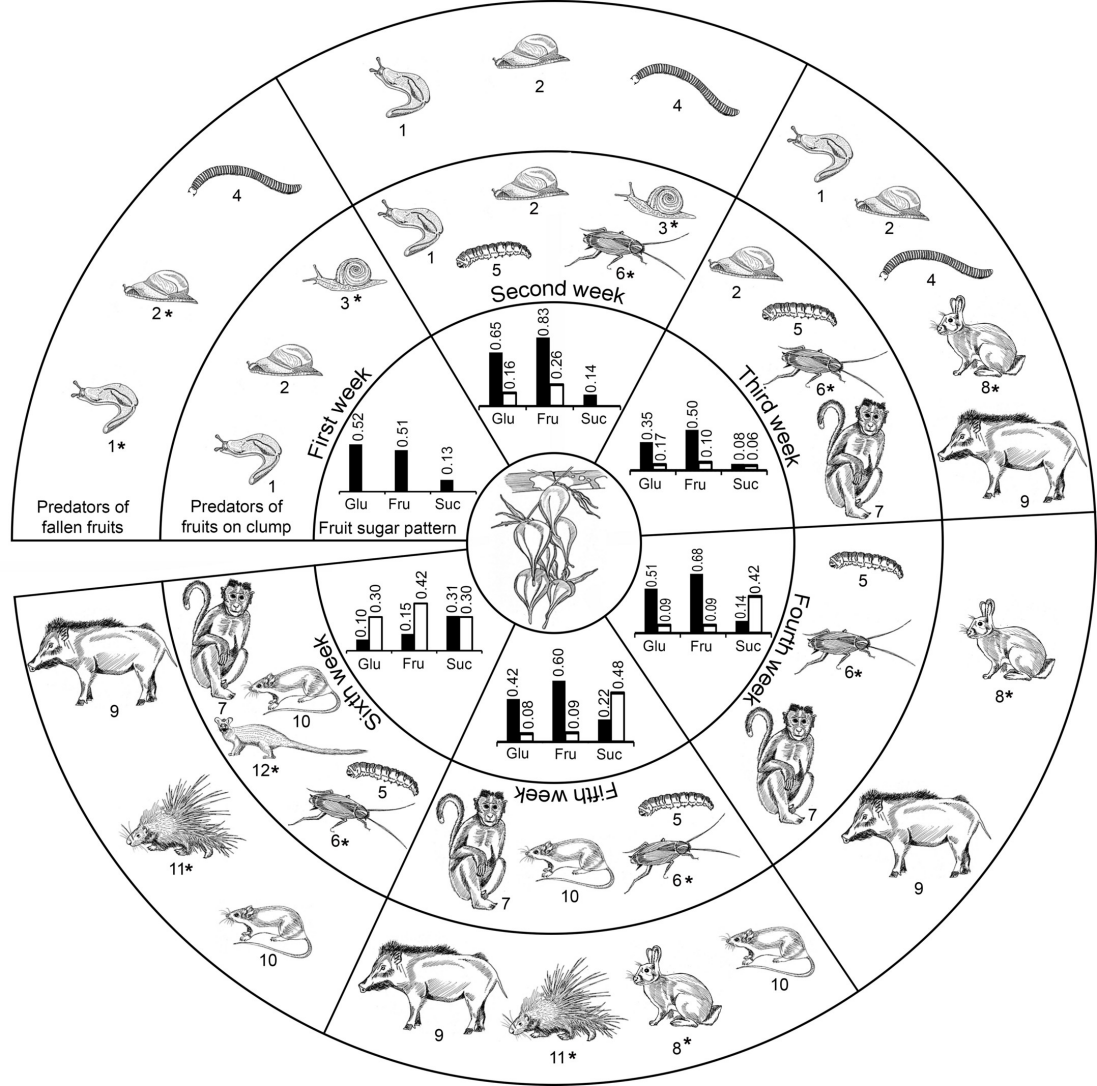
Diagrammatic sketch displaying predation on *M*. *baccifera* fruits and their sugar profiles. ***Predators*:** 1. Slug, *M*. *dussumieri*; 2–3. Snails, 2. *C*. *bistrialis*, 3. *Macrochlamys* sp.; 4. Millipede, *S*. *colosseus;* 5–6. Borer larvae, 5. *A*. *grisella*, 6. *B*. *germanica*; 7–12 Mammals, 7. Bonnet macaque, *M*. *radiata*, 8. Indian hare, *L*. *nigricollis*, 9. Wild boar, *S*. *scrofa*, 10. Rats, *R*. *rattus*, 11. Porcupine, *H*. *indica*, 12. Palm civet, *P*. *hermaphroditus*. Rabbits (*L*. *nigricollis*) and deers (*A*. *axis*) (not in figure) are primarily seedling predators and occasionally frugivorous (Table 1 and S10 Table in S1 File). *Infrequent visitors. ***Sugar pattern*, *predators*:** 1^st^ week: fruit pericarp Glu 0.52%, Fru 0.51%, Suc 0.13%, (predators 1–4), (no fruit liquid in first week); 2^nd^ week: fruit pericarp Glu 0.65%, Fru 0.83%, Suc 0.14%, fruit liquid Glu 0.16%, Fru 0.26%, Suc 0.00%, (predators 1–6); 3^rd^ week: fruit pericarp Glu 0.35%, Fru 0.50%, Suc 0.08%, fruit liquid Glu 0.17%, Fru 0.10%, Suc 0.06% (predators 1–2, 4–9), 4^th^ week: fruit pericarp Glu 0.51%, Fru 0.68%, Suc 0.14%, fruit liquid Glu 0.09%, Fru 0.09%, Suc 0.42% (predators 5–9), 5^th^ week: fruit pericarp Glu 0.42%, Fru 0.60%, Suc 0.22%, fruit liquid Glu 0.08%, Fru 0.09%, Suc 0.48% (predators 5–11), 6^th^ week: fruit pericarp Glu 0.10%, Fru 0.15%, Suc 0.31%, fruit liquid Glu 0.30%, Fru 0.42%, Suc 0.30% (predators 5–7, 9–12) (details of fruit sugar pattern in Govindan et al [9]).

**Fig 5:**
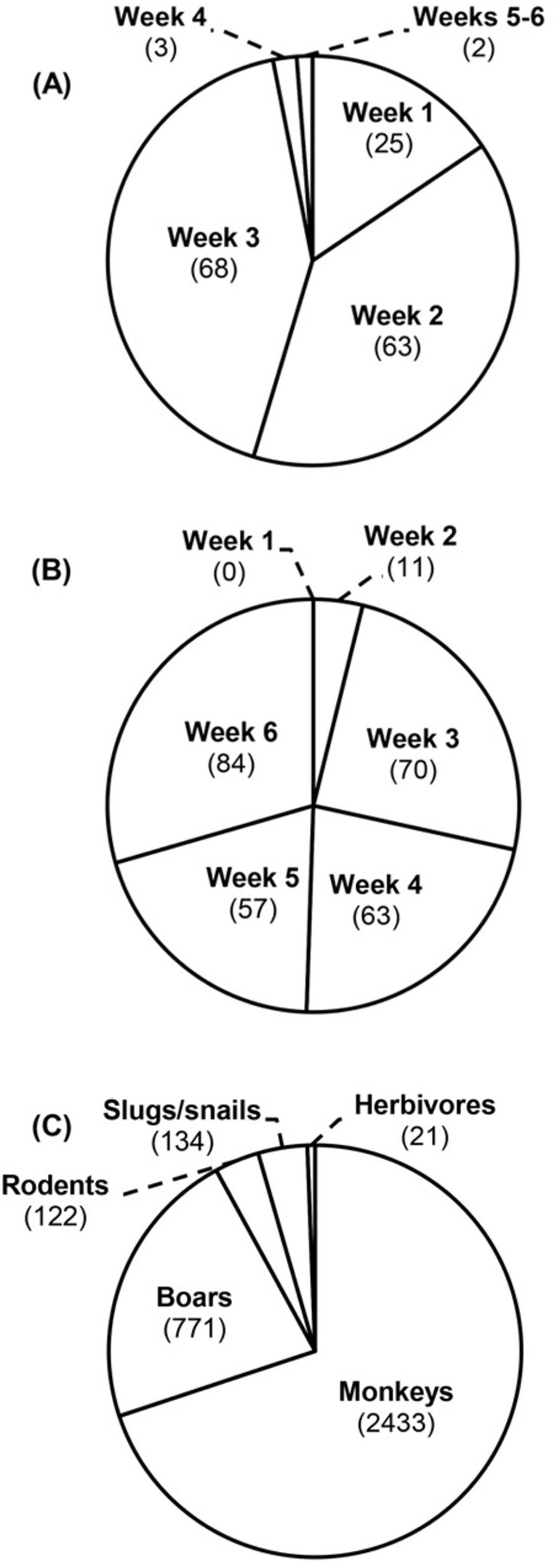
Slug/snail, borer and mammal predation patterns. A. Slug/snail attack in clump 58 (161 hits) (details in S9 Table in S1 File), B. Borer attack in clump 58 (285 hits) (details in S11 Table in S1 File), C. Predator attack in clump 359 (3481 hits) (details in S10 Table in S1 File).

*Slugs*, *snails*. One slug species (*Mariaella dussumieri*) and two snail species (*Cryptozona bistrialis*, *Macrochlamys* sp.) were found attacking young fruits. It was observed that the swollen soft pericarps of immature fruits were pierced or chewed by slugs and snails during rainy and/or wet days, and they drank the liquid inside the fruit. Fruits attacked by slug/snail left characteristic depressions or cavities on fruit pericarps which were easily recognizable (Figs 3 and 4 and S8(A)-S8(F), S9(A)-S9(C), S10(A)-S10(D) Figs in S1 File and Table 1). Of the total 2590 fruits surveyed in clump 58, from August 2010 to August 2011, 161 fruits (6.22%) were predated by slugs and snails (S9 Table in S1 File). Fruits at the age of 1–7 days (1^st^ week) registered 4.88% (25 out of 512 fruits), 8–14 days (2^nd^ week) 10.73% (63/587) and 15–21 days (3^rd^ week) 11.91% (68/571) hits. Slug/snail attack was negligible (0.54%; 5/920) in 4, 5 and 6 week old fruits (Fig 5A and S9 Table in S1 File). Maximum slug/snail attack was observed in the rainy months of July 2011 (13.57%; 38/280), June 2011 (10.43%; 34/326) and August 2010 (8.84%; 42/475). Slug/snail predation was also observed in clump 359 from May 2009 to December 2011. Of the total 3481 predated fruits, slugs and snails attacked 134 (3.85%) fruits. June-July registered highest hits, 18 out of 134 (13.43%) in 2009, 38 (28.36%) in 2010 and 34 (25.37%) fruits in 2011. This is followed by August with 3.73% (5 fruits) in 2009, 2.24% (3) in 2010 and 7.46% (10) in 2011 (S10 Table in S1 File). Thus, slug/snail predation is mostly confined to 1 to 3 week fruits and wet/rainy seasons (Figs 4 and 5A and Table 1).

*Borers*. The borer larvae attacked 7.87% fruits (285/3623) in clump 58, from January to December 2011 (S11 Table in S1 File). The attack started mildly (3.86%,11/285) during 8–14 days of fruit growth, gradually increased during 3–5 weeks (15–35 days) and peaked (29.47%, 84/285) during 6^th^ week (Figs 3, 4 and 5B and Table 1 and S11 Table in S1 File). These borer larvae created holes in growing fruits. They tunnel into the fleshy pericarp making irregular cavities, live inside and transform into adults. The larvae of *Achroia grisella* made borer holes on fruits leaving their excreta on the pericarp, whereas *Blattella germanica* (larvae) made holes without leaving excreta (Figs 3 and 4 and S11(A)-S11(D), S12(A), S12(B) Figs in S1 File and Table 1). Of these two borers, *A*. *grisella* caused more damage to fruits. Majority (85.26%, 243/285) attacks were during pre-monsoon period (April-May), which gradually decreased and ended by August (S11 Table in S1 File). No borer attack was observed during rest of the year. It is also noted that borer attack was not common to all clumps and not reported in all fruiting years.

**Fig 6:**
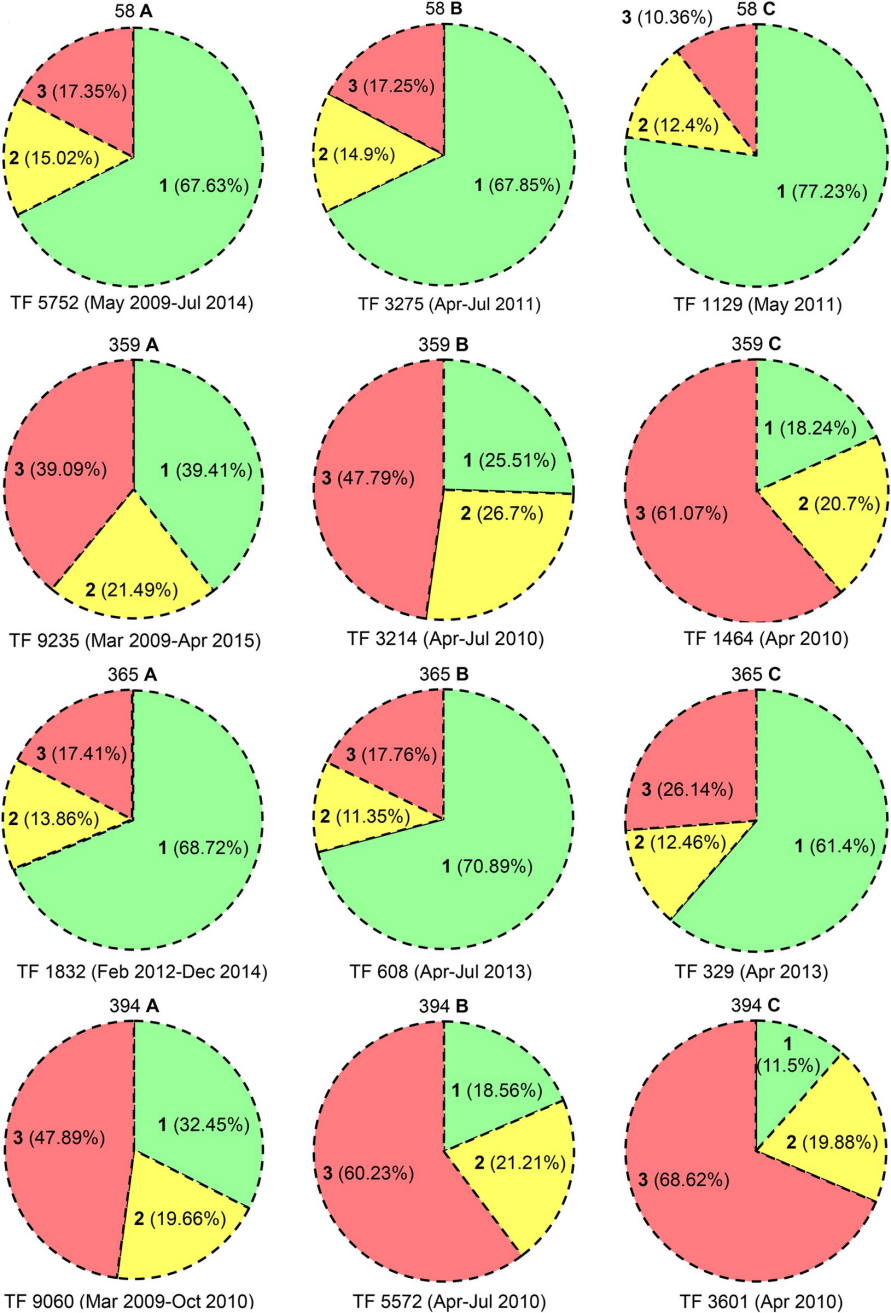
High reward, high predation. *M*. *baccifera* clumps: 58, 359, 365, 394; A. TF—*Total fruits*; **1**, % GF (*Good fruits*), **2**, % IF (*Immature fruits*), **3**, % PF (*Predated fruits*) in the full flowering period of the clump; B. TF—*Total fruits*; **1**, % GF, **2**, % IF, **3**, % PF in April-July of the year in which the clump recorded highest fruit production in a single month; C. TF—*Total fruits*; **1**, % GF, **2**, % IF, **3**, % PF in a single month of highest fruit production.

**Fig 7:**
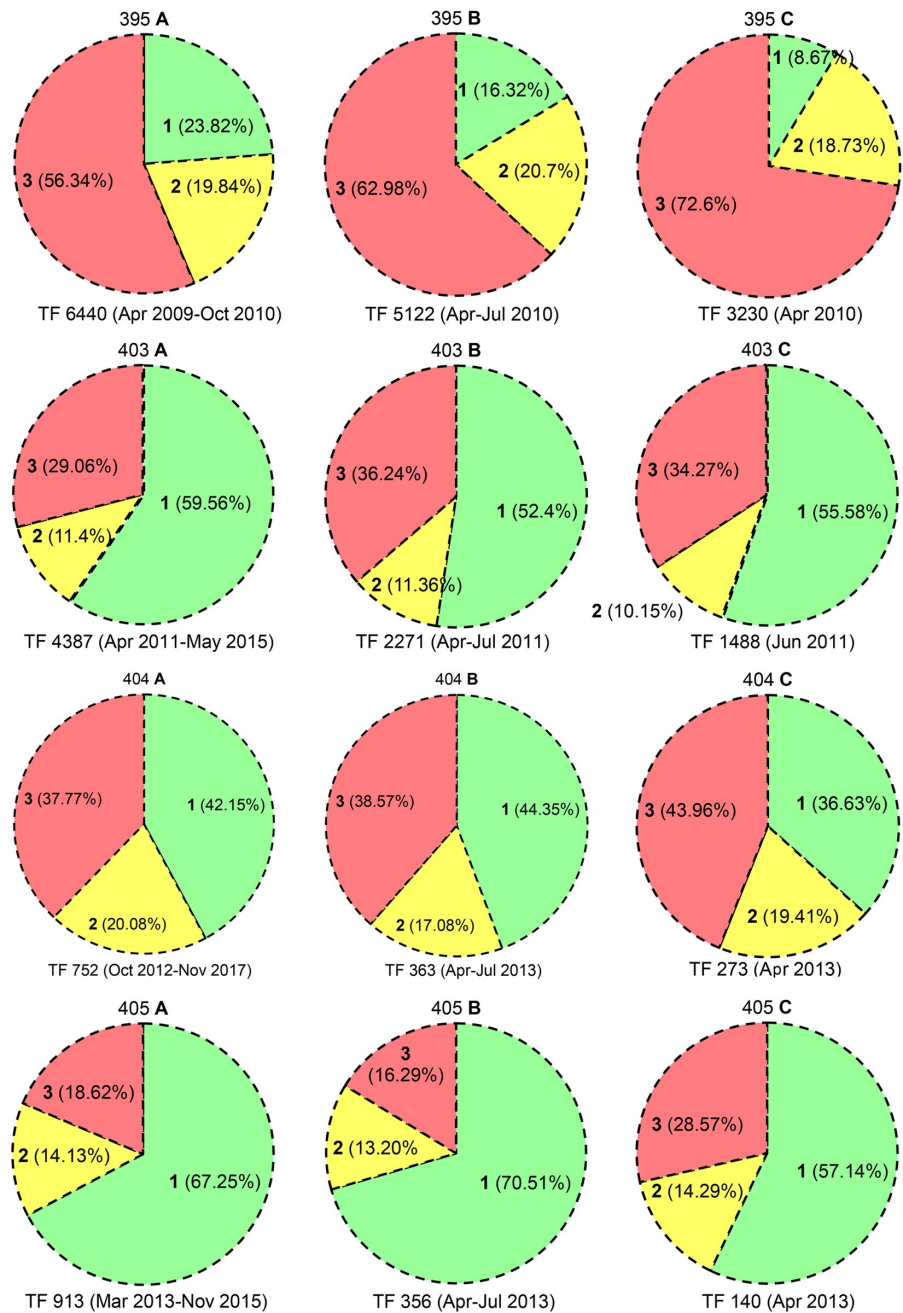
High reward, high predation. *M*. *baccifera* clumps: 395, 403, 404, 405; A. TF—*Total fruits*; **1**, % GF (*Good fruits*), **2**, % IF (*Immature fruits*), **3**, % PF (*Predated fruits*) in the full flowering period of the clump; B. TF—*Total fruits*; **1**, % GF, **2**, % IF, **3**, % PF in April-July of the year in which the clump recorded highest fruit production in a single month; C. TF—*Total fruits*; **1**, % GF, **2**, % IF, **3**, % PF in a single month of highest fruit production.

*Mammals*. Mammal visits correspond to the growth stage (age) of fruits (Figs 3 and 4 and S13(A), S13(B)-S17(A), S17(B) Figs in S1 File and Table 1), and their predation data in clump 359 are listed in S10 Table in S1 File. In 2009, the first year of fruiting, highest monthly predation (2009, 59.34%, 235/396 fruits) was observed in August, that of 2^nd^ year (2010, 61.07%, 894/1464 fruits) was recorded in April and 3^rd^ year (2011, 45.19%, 498/1102 fruits) in June. June to September accounted for high predation in 2009, April to July in 2010 and May to September in 2011. Considering the number of attacked fruits, monkeys were the major predators (2433/3481, 69.89%) and their highest monthly predation was in August 2009 (160 fruits), April 2010 (745 fruits) and June 2011 (376 fruits) (Fig 5C and S17 Fig in S1 File). Highest monthly predation by boars, the 2^nd^ highest consumer, was observed in August 2009 (63 fruits), April 2010 (135 fruits) and June 2011 (108 fruits) (S14(A), S14(B), S15(A), S15(B) Figs in S1 File). In rodents (rats, porcupines), the 3^rd^ important predators, highest predation was noticed in September 2009 (8 fruits), May 2010 (14 fruits) and June 2011 (12 fruits) (S13(A), S13(B) Fig in S1 File). The single month of highest predation (S10 Table in S1 File) is April 2010, *viz*., GF 18.24%, IF 20.70%, PF 61.07% (894/1464). Of these 894 fruits predated in April 2010, 745 (83.33%) were hit by monkeys and 135 (15.10%) by boars. Briefly, in short time frames of highest fruit production, the predation rates (S10 Table in S1 File; Apr 2010, PF%: 61.07%; May 2009-Dec 2011, PF%: 42.34%) are very high. All predators, particularly mammals, are seeking ‘high rewards’ (fruits) (Figs 6 and 7).

We observed frequent visits of troops of bonnet macaque monkeys, *Macaca radiata*, to fruiting clumps. They caused heavy loss of fruits on clumps as evidenced by the sizable amount of fruit fragments left with characteristic bites (Figs 3H and 4 and S17(A), S17(B) Fig in S1 File). Hoof marks and peculiar soil rooting characteristics of wild boar, *Sus scrofa* (Figs 3K, 3L and 4 and S14(A), S14(B), S15(A), S15(B) Figs in S1 File), were found around clumps particularly in wet soil conditions. Their presence was confirmed by direct sightings during late evenings. Boars also swallowed entire fruits, leaving no remnants, and they could not be counted. Seedling predation by *S*. *scrofa* was also observed by us.

Rats and other rodents, which are night foragers, left characteristic gnawing bites in fruit remains (Figs 3I and 4 and S13(A), S13(B) Fig in S1 File). *M*. *baccifera* fruiting resulted in a hike in the number of rats in the Bambusetum field area. Rats were identified as *Rattus rattus*. Quills (made of keratin) of the large rodent, the Indian crested porcupine, *Hystrix indica*, were found below clumps confirming their visits and predation (Figs 3J and 4 and S16(A), S16(B) Fig in S1 File). Occasionally in some clumps, we observed almost complete disappearance of fallen fruits and fruits on lower culm nodes during night times. We sighted the common palm civet or toddy cat, *Paradoxurus hermaphroditus*, frugivorous and active after sunset, in the 6^th^ week (36–42 days) of fruit growth (Fig 4). Predation on seedlings by two herbivores, the common rabbit species *Lepus nigricollis* (Indian hare or the black-naped hare) and the deer, *Axis axis* (chital deer or spotted deer or axis deer), both native to the Indian subcontinent, was also observed. They browsed self-grown seedlings from fortuitously left over fruits or viviparous fruits (5–10 fruits yearly) (Table 1 and S10 Table in S1 File). We could not make detailed quantitative accounts of fruit predation by the palm civet and seeds/seedling predation by wild boar, rabbit and deer.

*Millipede*. The fruit remains were consumed by the colossal slender spined millipede, *Spinotarsus colosseus* (Fig 3E and S18(A), S18(B) Fig in S1 File).

#### Fruit production dynamics

Mature fruit (Fig 3A) is an ovoid to globose baccate caryopsis, pear-shaped with a tapering curved beak, glabrous and smooth; pericarp is fleshy and thick; fruit colour ranges from brown (S1A Fig in S1 File), brownish-green (S1B-S1C Fig in S1 File) to pure green (S1D Fig in S1 File). Length, breadth and weight of mature fruits (42 days) in earlier study [9] were 9.93 ± 1.51 cm, 5.49 ± 0.62 cm, 104.39 ± 26.96 g; seed weight: 6.09 ± 1.92 g (5.83% of fruit wt.) (n = 30). Average weight of mature fruits in the present study is 49.45 ± 25.75 g (n = 500).

We could not observe any specific pattern in fruit production among the clumps. Clump 58 produced 5752 fruits (284.44 Kg; weight 49.45 ± 25.75 g, n = 500) in 6 years; 68 in 1^st^ year (1.18%), 258 in 2^nd^ year (4.49%), 4133 in 3^rd^ year (71.85%), 1177 in 4^th^ year (20.46%), 105 in 5^th^ year (1.83%) and 11 in 6^th^ year (0.19%) (S3 Table in S1 File). Year-wise highest production in clump 58 was registered in May-August 2009 (67), July-October 2010 (163), May-August 2011 (3456), April-July 2012 (939), April-July 2013 (96), April-July 2014 (10), accounting for a total 4731 fruits (82.25% of total fruit production), whereas the first (January-March) and last (October-December) quarters recorded low fruit production. Fruit production in clumps 359 (456.67 Kg), 365 (90.59 Kg), 394 (448.02 Kg), 395 (318.46 Kg), 403 (216.94 Kg), 404 (37.19 Kg; clump alive, fruiting continues) and 405 (45.15 Kg) are listed in S3 Table in S1 File. Eight clumps together produced 38371 fruits weighing 1897.45 Kg. Average life time production per clump is 4796.38 (± 3420.26, n = 8) fruits, weighing 237.18 (± 169.13 Kg, n = 8) (S3, S5 Tables in S1 File).

Of the eight clumps studied, three clumps produced maximum fruits in 1^st^ (calendar) year *viz*., 365 (1046, 57.10%), 403 (2606, 59.40%), 405 (400, 43.81%), four clumps in 2^nd^ year *viz*., 359 (3381, 36.61%), 394 (5675, 62.64%), 395 (5216, 80.99%), 404 (395, 52.53%), and one clump in 3rd year (58, 4133, 71.85%) (S3 Table in S1 File). Fruit production, high in the first three years, continued further with low output up to seventh year in clump 359 and is still continuing (10 years in August 2022) in 404 (S4 Table in S1 File). Low fruit production occurred generally during drier months January and February [58 (88, 1.53%), 359 (89, 0.96%), 365 (19, 1.04%), 394 (13, 0.14%), 395 (3, 0.05%), 403 (50, 1.14%), 404 (4, 0.53%), 405 (31, 3.40%)] and October to December (example, 58: 2009, 2013, 2014, 0–1 fruit) (S3 Table in S1 File).

Flowering initiated in six clumps during January-March, others in April (58) and September (404) (S3, S4 Tables in S1 File). This means, the flowers responsible for highest fruiting in April (2010, 2013) were originated during the preceding summer months (February and March). After initiation, the pre-monsoon showers (April-May) and southwest Monsoon (June to August) exert a direct stimulus on fruit development as April, May, June, July months registered highest production *viz*., 58 (4453, 77.42%), 359 (7333, 79.40%), 365 (1654, 90.28%), 394 (7658, 84.53%), 395 (5765, 89.52%), 403 (3647, 83.13%), 404 (611, 81.25%), 405 (770, 84.34%). More than 80% of total fruit production occurred during April-July. In few clumps, high fruit production extended to August and November (S3 Table in S1 File).

In six clumps highest single month fruit production was observed in April (359: Apr 2010, 1464 nos; 365: Apr 2013, 329; 394: Apr 2010, 3601; 395: Apr 2010, 3230; 404: Apr 2013, 273; 405: Apr 2013, 140); in clumps 58 and 403, highest single month production was observed in May 2011 (1129 fruits) and June 2011 (1488 fruits), respectively (Figs 6 and 7 and S3, S6 Tables in S1 File). It is interesting that, clump 395 produced 3230 fruits (50.16% of its total production) in one month (April 2010) of its 19 month fruiting period. Clumps 394 (3601 fruits, 39.75%), 403 (1488, 33.92%) and 404 (273, 36.30%) also produced over 30% fruits in one month of their fruiting periods (S3-S7 Tables in S1 File). Total fruit production in the one month of peak fruit output in 8 clumps is 11654, which is 30.37% of their total life time fruit production (S7 Table in S1 File). Strikingly, the total fruit predation in this one month of highest fruit production is 6583, which is 45.02% of their life time predation (14623) (S3-S5, S7 Tables in S1 File and Figs 6 and 7).

*Good*, *immature and predated fruits*. Three clumps recorded above 60% good fruits (GF), *viz*., 67.63% (58), 68.72% (365) and 67.25% (405). GF in clump 403 was 59.56%, clump 404 (42.15%), 359 and 394 were above 30% (39.42%, 32.45%) and clump 395 recorded the lowest 23.82%. GFs of all 8 clumps were 43.80% (16807/38371) (Figs 6 and 7 and S3, S5 Tables in S1 File). Fall of immature fruits (IF) ranged above 20% in 2 clumps (359: 21.49%, 404: 20.08%), 15–20% in three clumps (clump 58: 15.02%, 394: 19.66%, 395: 19.84%) and less than 15% in three clumps (405: 14.13%, 365: 13.86%, 403: 11.40%). IFs from 8 clumps were 6942, which is 18.09% of total fruit production (38371) (Figs 6 and 7 and S3, S5 Tables in S1 File). Severe predation (c. 50%) was observed in two clumps 395 (56.34%) and 394 (47.89%), moderate predation (29–40%) in three clumps, 359 (39.09%), 404 (37.77%) and 403 (29.06%) and mild predation (< 20%) in three clumps, 405 (18.62%), 365 (17.41%) and 58 (17.35%). Of the total 38371 fruits produced 14623 (38.11%) were predated. GF/PF ratios in clumps were (58: 67.63%:17.35%), (359: 39.42%:39.09%), (365: 68.72%:17.41%), (394: 32.45%:47.89%), (395: 23.82%:56.34%), (403: 59.56%:29.06%), (404: 42.15%:37.77%) and (405: 67.25%:18.62%) (Figs 6 and 7 and S3, S5 Tables in S1 File).

*Clump death*. Clumps die progressively; flowering starts initially in a few culms (aerial stems), continues for one to three years, ceases slowly and then the culms die. More culms follow this pattern in successive years, and ultimately all culms in a clump die (clump death). Clump 395 died in the same month of flowering cessation, four clumps (359, 365, 394, 405) died after one month, 403 after two, 58 after five months and clump 404 is still alive (as on August 2022) (S3, S4 Tables in S1 File).

#### Fruit chemistry, rat feeding preferences, hematological/biochemical parameters

Secondary metabolites (fruits, leaves), nutritional parameters (fruits) and rat feeding preferences (fruit, fruit liquid, seed; Glu, Fru, Suc) were analyzed [9, 21] and the fruit-visitor/predator interactions were interpreted accordingly.

## Discussion

### Flowering phenology

Most clumps flowered in January to April (prior to southwest monsoon), and flowering durations varied significantly between them. Three types of florets (staminate, pistilate, bisexual) were found in *M*. *baccifera*, and our observations on types of florets are in general agreement with Ramanayake and Weerawardene [29]. The flowering pattern is comparable with *Ochlandra scriptoria* (fleshy fruited reed bamboo) reported from the same study area. *O*. *scriptoria* initiated flowering in December, produced highest number of pseudospikelets in March-April and maximum fruits in May-June. During rainy season (June-July), *O*. *scriptoria* culms ceased flowering, remained vegetative and new shoots were produced in the flowering clumps [30]. Gadgil and Prasad [31] reported similar phenology in *Bambusa arundinacea* (= *B*. *bambos*) and *Dendrocalamus strictus*. It is interesting that most bamboos, irrespective of their taxonomic status and nature (size, fleshy or dry fruits), follow similar flowering/fruiting phenology.

Our observations on the extended flowering durations ranging from 1 year 8 months to 10 years is very significant as an earlier report specified that *Muli* flowers only for one year and rarely exceeds this period [2]. This highlights the need for prolonged field observations in bamboo reproductive biology. In *B*. *bambos* and *D*. *strictus* 70 percent of clumps fruited for one or two years, and only c. 8% lasted for 4–5 years [31]. These observations indicate that clumps of the same cohort with different flowering durations can co-occur in native areas. As flowering cycles are determined from the period of two successive flowerings in a region, variations in flowering durations among clumps could have resulted in determining different flowering cycles in *M*. *baccifera* [2–5]. In this study, the striking variations in flowering periods occurred in a small area demonstrate the influence of habitat. Two clumps of common origin displayed marked variations in flowering periods (403: 4 years 4 months, 404: 10+ years, live), illustrating the influence of microclimate in flowering durations [32, 33].

The breeding system in *M*. *baccifera* is striking. The temporal separation of female and male stages by two to four days (3304.32 ± 1012.02 min, n = 37) clearly testifies the dichogamous nature of bisexual florets and the appearance of female stage (exposure of stigma) first, prior to stamen exposure, affirms protogyny. Dichogamy and protogyny are very common in bamboo genera, examples: *Ochlandra* [30, 34], *Phyllostachys* [35], *Pseudoxytenanthera* [36] and *Dendrocalamus* [37, 38]. Moreover, the temporal separation of pistil and stamens (3304.32 ± 1012.02 min, n = 37) and fertilization and development of ovary before anthesis prevent selfing or inbreeding [34, 37–40]. These are also contrivances for outcrossing creating genetic variability in the species. Again, the female stage duration (1790.54 ± 657.75 min, n = 37) is c. 7 times or more than that of male stage (261.81 ± 58.58 min, n = 37) means a pistil can accept pollen grains from several successive male flowerings (geitonogamy). The positioning of male and female florets, i.e. spatial separation, is also very significant. The florets borne on upper branches are numerous and predominantly males. The female and bisexual florets are in lesser numbers and held at lower levels. So a pistil has greater opportunity for receiving pollens from multiple male florets, which surely is an adaptation towards effective pollination and production of more fruits.

### Flowering/fruiting intervals, ‘biological clock’

Mast flowering of bamboos is an intriguing phenomenon. It happens wherever large extents of natural bamboo exist and all individuals of a cohort flower synchronously and die. Janzen [4] speculated that flowering in bamboos is governed by an ‘internal physiological calendar’, and thereafter several authors supported this hypothesis [29, 41, 42]. All clumps in our study, propagated vegetatively, flowered in synchrony (2009–2013) with their native populations, after 13–21 years of their introduction (1988–96) from NE India (S1, S4 Tables in S1 File).

### Fruit production dynamics

Janzen [4] and Alam [43] reported fruit weights in *M*. *baccifera* as 100–350 g and 47–180 g (sample size unknown in both cases), respectively. Banik [2] provided fruit length, diameter and weight as 3.5–11.0 cm, 2.2–6.0 cm and 7–151 g (in one case 300 g) (sample size 10,000), respectively. Again, he specified that fruits produced in May-June are bigger (length 6.9 ± 0.3 cm, diameter 4.1 ± 0.2 cm) and heavier (55.3 ± 5.4 g). Average fruit weight in our earlier assessment [9] was 104.39 ± 26.96 g (mature fruits, sample size 30; initial years of fruiting). In the current study with a larger sample size (mature fruits, n = 500, 8 clumps; later years of fruiting) average fruit weight was only 49.45 ± 25.75 g. These data indicate that fruits produced in initial years are bigger and those of later years are smaller. Thus, our data are in general agreement with earlier reports [2, 4, 43]. The variations in fruits (length, weight, shape, colour) among clumps can be attributed to environmental and genetic (different ploidy levels, 6x and 8x, x = 12) factors. For example, clump 58, which produced brown, globose (brownish-green, green and ovoid in others) fruits, is an octoploid (2n = 96) [44]. The fruit variations could also be an adaptation to cater the variety of mammal predators.

The average fruit production per clump is 4796.38 (± 3420.26, n = 8) fruits, weighing 237.18 (± 169.13 Kg, n = 8), against the previous report of 25 to 40 Kg [2]. The clumps showed much higher output (37.19 to 456.67 Kg) with regard to both per clump and per season fruit productions. The primary reason for this unmatched greater fruit production is that the clumps in our study are in a protected area (Bambusetum) and fruits were collected on a daily basis. In contrast, seed production in species with dry caryopsis ranges from 92 to 103.5 Kg (*B*. *bambos*) and 145.15 Kg (40 square yard clump, *D*. *strictus*) [4, 31]. We report the highest single clump production of 456.67 Kg fruits (clump 359) in *M*. *baccifera*, a record among bamboos. Again, high fruit production was during first three years, and very low fruit output was observed for rest of the period (four to ten years). Significantly, one month of highest fruit production (11654, eight clumps; S5, S6 Tables in S1 File) accounted for 30.37% of total life time production (38371), and corresponding predation (6583) was a high 45.02% of the life time predation (14623) (S5, S6 Tables in S1 File). These data clearly infer that the glut of these fleshy fruits generated in a short span (of one month) entices the predators. Otherwise, predators are seeking a ‘high reward’ (of fruits).

Again, in six clumps, the florets responsible for highest fruiting in April were initiated during February and March (summer months). In other two clumps (58, 403: highest fruiting: May, June) florets were initiated in March-April and April-May. This indicates that high temperature, humidity, sun light and intermittent summer rainfall have significant influences in triggering the ‘coordinated genetic activity’ (releasing the molecular resources), leading to flowering and fruiting [45–48, our field observations]. Highest fruit production during April-July and very low fruit production during summer months point to the influence of monsoon showers. The continued high fruit production in some clumps/years could be due to extended rains and other microclimatic/genetic/ecological variations of respective clumps [46, 48]. This is in general agreement with the observations of Banik [2], Gadgil and Prasad [31] and Koshy and Harikumar [30]. These findings are valuable for foresters indulged in the regeneration or conservation of this species in the native areas.

### Total, good, immature and predated fruits

The grading of fruits (GF, IF, PF) reflects their respective proportions (43.80%, 18.09%, 38.11%) in a typical mast seeding scenario. It is noteworthy that c. 20% of fruits fall prematurely and c. 40% are vulnerable to predation. Despite the huge fruit output, the survival of good fruits is only < 50% (fruits were picked up on a daily basis) (Figs 6 and 7 and S3, S5 Tables in S1 File). Strangely enough, even in such conditions, few fruits tend to escape from the collector’s and predator’s sights and develop into seedlings; this observation strengthens the predator satiation hypothesis [4, 49].

Clumps 359 and 394 produced 9235 and 9060 (> 9000) fruits in their total fruiting period, 6 years 2 months and 1 year 8 months, respectively (S7 Table in S1 File). The TF, GF%, IF% and PF% in these clumps are (359: 9235, 39.42%, 21.49%, 39.09%) and (394: 9060, 32.45%, 19.66%, 47.89%), respectively. The PF% in 359, which produces highest number of fruits (9235 in 74 months), is relatively low (39.09%) compared to 394 (47.89%) with nearly same fruit output in a shorter period (9060 in 20 months). Moreover, in these 2 clumps, the TF, GF%, IF%, PF% in April-July 2010 (year in which single month highest fruit production was observed) are 359: 3214, 25.51%, 26.70%, 47.79% and 394: 5572, 18.56%, 21.21%, 60.23%. The single month of highest fruit production in these two clumps is April 2010; and the TF, GF%, IF%, PF% in this month are 359: 1464, 18.24%, 20.70%, 61.07% and 394: 3601, 11.50%, 19.88%, 68.62%. PF% in 359 and 394 jumped 21.98% and 20.73% in the single month of highest fruit production (April 2010) compared to their lifetime PF% (S3, S5-S7 Tables in S1 File and Figs 6 and 7). These clumps with the highest fruit production displayed the highest PF% (among the 8 clumps), and these PF% are showing considerable increments in the highest fruit producing season (April-July 2010) and in the single month of highest fruit production (April 2010). The third highest fruit production (total flowering period) was observed in clump 395, and its TF, GF%, IF%, PF% are 6440, 23.82%, 19.84%, 56.34%. PF% in 395 went up to 62.98% and 72.60% during April-July 2010 and in April 2010, the month of its highest fruit production. In clumps 359, 394 and 395, the PF% is enhanced in short periods of high fruit production, and there is a proportional decrease in GF% (359: 39.42 to 18.24% (April 2010); 394: 32.45 to 11.50% (April 2010); 395: 23.82 to 8.67% (April 2010)) (S3, S5-S7 Tables in S1 File and Figs 6 and 7).

Clumps 58 and 403, which are more exposed to disturbances (garden visitors, vehicles), did not show high levels of fruit predation (PF%) in their entire flowering periods (58: 17.35%; 403: 29.06%), whereas clumps 359, 394 and 395 are well within the surrounding wild flora. Clumps 365, 404 and 405 exhibited comparatively low fruit production rates, and displayed low PF% of 17.41%, 37.77% and 18.62% in their entire flowering periods (S3, S5-S7 Tables in S1 File). Thus, low fruit output and high disturbances result in low predation. Curiously, IF% was about 20% in these eight clumps in their total flowering period(s), season(s) and month(s) of highest fruit production (S3, S5-S7 Tables in S1 File and Figs 6 and 7). These fruit dynamics data again prove high predation in shorter windows of higher fruit production; or otherwise, predators are driven by high rewards (fruits).

### Clump death

Most clumps died quickly after fruiting ended (0–5 months) (S4 Table in S1 File) as in *B*. *bambos* and *D*. *strictus* [31]. Clump death following flowering, common in woody bamboos [3–5, 31], is reflected here also.

### Floral visitors, predators, inhabitants

#### Bees

We recorded four bee species, *viz*., *A*. *cerana indica*, *T*. *iridipennis* (honey bees), *H*. *taprabonae* and *B*. *cupulifera*, visiting *M*. *baccifera* florets. An earlier study [38] on bee visitations in six woody bamboos in the same Bambusetum documented 10 bee species of five genera (predominantly of order Hymenoptera) *viz*., *Apis* (2 spp.), *Halictus* (4), *Trigona* (1), *Braunsapis* (2), *Ceratina* (1). The reed bamboo *Ochlandra travancorica* attracted maximum number of bee species (6), and *A*. *cerana* was the common bee visiting almost all species [30, 40, 50]. Though bees never visited the stigma effecting pollen transfer, their throbbing of anthers help release of pollen grains into the air which possibly fall upon exposed stigmas facilitating indirect pollination [30, 35, 40, 50–52]. Bamboo florets lack nectar, scent and bright colour, and they own pollen grains as the only reward to offer. At this point, an explanation to how bees and other visitors are attracted [4] towards the flowering clumps situated amidst the wild flora in the Bambusetum can be found in the study by Baby and co-workers [53]. They reported UV induced blue fluorescence emission patterns from grass reproductive structures including bamboos which may function as visual cues attracting pollen and seed predators towards grass flowers and grains.

The role of bees in bamboo pollination is an argued issue [51, 54] and most authors are proponents of wind and insect-assisted pollination [35, 37, 40, 51, 52], while a few support anemophily alone [55, 56]. We grouped *M*. *baccifera* under wind and insect-assisted pollination category. All these reports pose an interesting question, whether the transition between wind and animal pollination [57] is evolving in bamboos?

#### Mantis

Mantis (*E*. *indica*) has strong preference to honey bees [58] and it catches live prey by a careful and swift grasping action [59]. By doing so, to certain extent they control the destructive foraging [30] of bees, saving some amount of pollen. Also, their rapid, jerky movements help release pollen grains, and thereby aiding pollination.

#### Ants

Among the two ant species found inhabiting *M*. *baccifera* clumps, *O*. *smaragdina*, distributed widely in Asia and Australia, is arboreal and makes nests out of leaves sewed together using the silk produced by their larvae [60]. *C*. *biroi* native to India and China, nests in soil and are fond of sugars [61], and it is not so far reported from any other bamboo [62]. Ants, being insect/pest predators, function as biological control agents [60, 63] and protect clumps from pest attack.

### Fruit predators

#### Slugs, snails

Slug and snails attack only young fruits (1–3 weeks) and display their affinity to fruit liquid; their predation is so far not reported in other bamboos.

#### Borers

The two borers (*A*. *grisella*, *B*. *germanica*) attack the fruits mildly (11/285; 3.86%) in 2^nd^ week (8–14 days), their attack surges in 3–5 weeks (15–35 days; 24.56%, 22.11%, 20.00%) and peaks (84/285; 29.47%) during the 6^th^ week (S11 Table in S1 File). They pierce into the fruit when the pericarp is soft and fleshy and live inside till the fruit attains maturity. This indicates that they relish the fruit liquid and gradually grab the developing fruit tissue. *A*. *grisella* is a known seed pest of *Ochlandra ebracteata* [64], a native bamboo having baccoid fruits, damaging 10–15% fruits [45]. Curiously, the same pest which attacked native bamboo attacked the introduced *M*. *baccifera* too. Insects alone damaged 5% seeds in *B*. *bambos* [31]. Thus, bamboo seed damage by insects is a serious issue, but poorly studied and in many cases insects remain unidentified [4].

#### Mammals

We reported five mammalian fruit predators, monkeys (*M*. *radiata*), rats (*R*. *rattus*), porcupine (*H*. *indica*), wild boar (*S*. *scrofa*), palm civet (*P*. *hermaphroditus*), and two mammalian seedling/fruit consumers, rabbit (*L*. *nigricollis*) and deer (*A*. *axis*). Earlier workers reported that *M*. *baccifera* fleshy fruits attract bison, deer, rats, wild boars, porcupines, Burmese rhinoceros and other animals, in addition to humans [2, 4, 65]. *M*. *radiata* population is on the decline all over India, but the forest areas bordering our Bambusetum situated at the southern end of Western Ghats maintain their population strength [66]. Fruits are the major components in their diet [67]. In this case, the limited number of *Muli* clumps at the edge of the forest lands of Western Ghats formed good foraging points for these monkey troops [68]. Wild boar (*S*. *scrofa*) has one of the widest geographic distributions, particularly in wet soil conditions [24]. During *O*. *travancorica* seeding in the same area, *S*. *scrofa* population increased and they heavily consumed its fruits [45]. Rodent (rats, mice, porcupines) predation alone caused 5 to 10% offspring loss in *Ochlandra* sp. [45], and they form major seed predators in *B*. *bambos* in India [31]. Previous studies also highlighted bamboo mast fruiting associated population explosions of rodents (examples, *R*. *rattus* and *R*. *norvegicus* flooding in Chile, *Rattus* population enhancement to 40–60 million in Madagascar) [4]. Rodents responsible for famine during bamboo flowering in NE India are *R*. *rattus* followed by *R*. *nitidus*, *R*. *niviventer* and *R*. *r*. *brunneusculus* [69]. We found *R*. *rattus* as the main rodent species feeding on *Muli* fruits.

#### Millipede

Millipedes (*S*. *colosseus)* that consumed leftover fruit remains are known primary decomposers, consuming 20 to 100% of plant debris and returning 60 to 90% organic matter in the form of faecal pellets, enriching the soil organic matter and nutrients [70]. Thus, they help increase soil fertility in the Bambusetum.

### Fruit dispersal, shift in predation pattern

The mature *M*. *baccifera* fruits disseminate by rolling down the hill slopes and through rain water currents [2], as observed in case of *O*. *scriptoria* [30]. The baccoid fruit shape is an advantageous factor facilitating this form of dispersal. We could not find any other agents, barring humans, involved in fruit dispersal. Janzen [4] considered humans as major predators of bamboo seeds; but Gadgil and Prasad [31] specified that humans collect only less than 1% of the total production, and hence man is not a significant predator. However, they reported huge collections by humans in 1865 and 1966–1967 when famines coincided with bamboo mast seeding [31]. *Muli* fruits have become a commercial produce and, in Tripura, during the mast seeding of 2002–2007, fruits were procured from local people (at INR 0.50 to 1.00/fruit), and were sold to various organizations in India and other countries for raising plantations [2]. We distributed 831.08 Kg fruits to forest departments, government agencies, NGOs and the public for cultivation. Such commodification of bamboo fruits results in increased collection by humans leaving only lesser quantities in the wild, thereby reducing predation by animals. Here, we perceive a gradual shift in predation, from animals to humans.

### Visitors, fruit chemistry, preferences

*M*. *baccifera* floral/fruit visitors ranged from arthropods to mammals (Table 1 and Figs 1–4). Govindan and co-workers [9] reported sugars and amino acids as the two major groups of fruit metabolites, along with proteins (low), phenolics, fatty acids, minerals and vitamins; and disproved the popular myth of ‘high protein’ fruits/seeds in *M*. *baccifera*. They also isolated 27 secondary metabolites from its fruits and leaves, including verbacine (polyamine) and 4-oxabicyclo[3.2.2]nona-1(7),5,8-triene [21]. In the present study, we observed a correlation between visitor (predator) hits and the metabolite (sugar) profiles of *Muli* fruits. Sugars displayed an interesting glucose (Glu)-fructose (Fru)-sucrose (Suc) pattern with the growth stage of the fruits. In *M*. *baccifera* fruit pericarps on the 7^th^ day of maturity, Glu-Fru-Suc contents were 0.52, 0.51 and 0.13%, respectively, whereas in fully matured fruit pericarps (42 days), their contents were 0.10% (Glu), 0.15% (Fru) and 0.31% (Suc) (Fig 4). Fruit liquid was not formed on the 7^th^ day of fruit growth, but developed by 14^th^ day and gradually transformed to the seed (by 35^th^ day). In fruit liquids, Glu-Fru-Suc contents were 0.16, 0.26 and 0.00% (14^th^ day), 0.08, 0.09 and 0.48% (35^th^ day, seed) and 0.30, 0.42 and 0.30% (42^nd^ day, seed), respectively (Fig 4) [9]. Glu and Fru showed gradual decrease (or fluctuation) in their levels with progressive development of fruits, whereas Suc content (fruit pericarp 0.13 to 0.31%; fruit liquid/seed 0 to 0.30%) showed an increase with fruit growth (Fig 4). The low level of Suc till 21 days could be attributed to Suc metabolism into Glu and Fru, and its high content (after 21 days) could be associated with Suc accumulation. The sweetness and quality of fruits are determined by sugars.

Slug (*M*. *dussumieri*) and snails (*C*. *bistrialis*, *Macrochlamys* sp.) were observed in very young fruits up to 21 days (1–3 weeks), and their attacks were minimal in near mature/mature (4–6 weeks) fruits (Table 1 and Figs 3, 4 and 5A). The limited literature on the sugar preferences of slugs and snails emphasizes sugar concentration (especially Glu and Fru) as a major factor in their food choices [71, 72]. In *M*. *baccifera*, fruits are fleshy and they offer preferential sugars, Glu and Fru, in the early 3 weeks. Larvae of moth (*A*. *grisella* or lesser wax moth) and cockroach (*B*. *germanica* or German cockroach) attacked the fruits with low incidences from 8–14 days (2^nd^ week), and their hits continued till the 6^th^ week (36–42 days) (Table 1 and Figs 3, 4 and 5B). Borer attacks were observed from April to August (and not in the rest of the year) (S11 Table in S1 File). Sugars (Glu, Fru, Suc) act as phagostimulants in cockroaches. Studies have shown that cockroaches develop optimally on diets with more than 50% carbohydrates, and ideal diets for the German cockroach included high levels of Glu or related sugars [73]. The time and frequency of attack of these two borers are clear indications of their sugar (Glu, Fru, Suc) preferences.

Most frugivorous mammals rely on soluble carbohydrates as a primary source of metabolic energy; and their availability and associated macronutrient composition are preference factors [74]. Mammals *viz*., monkeys (*M*. *radiata*), wild boar (*S*. *scrofa*) and rabbits (*L*. *nigricollis*) predate *M*. *baccifera* fruits from 15–42 days (3–6 weeks), 15–42 days (3–6 weeks) and 15–35 days (3–5 weeks) of growth, respectively (Table 1 and Figs 4 and 5C). Porcupines (*H*. *indica*) hit the fruits from 29–42 days (5–6 weeks) and palm civet (*P*. *hermaphrodites*) from 36–42 days (6^th^ week) (Fig 4). Rat (*R*. *rattus*) predation was observed in near mature and mature fruits (5–6 weeks, 29–42 days) (Table 1 and Figs 4 and 5C). Wild boars, porcupines and rats were found to attack (mature) germinating fruits (7^th^ week old) (Table 1); rats also attacked stored fruits. Monkey predation was observed only on fruits on the clumps, whereas rat predation was found both on fruits on the clumps and fallen fruits (Table 1 and Figs 3 and 4). Other mammals (wild boar, rabbits, porcupines) predated on fruits fallen on the ground (Table 1 and Fig 4). During the growth periods, the fruit liquid/seed and pericarp are sources of a unique sugar mix (Fru-Glu-Suc). Suc levels are high in mature (5–6 weeks) and germinating fruits (7^th^ week) [9], prompting rat predation (Table 1 and Figs 3 and 4).

Fruit liquid-water preference experiments in rats [9] demonstrated significantly higher (3.08 times) average fruit liquid intake (7.86 ± 1.73 mL) compared to water. In fruit liquid-Suc/Glu/Fru-water preference experiments, rats showed a higher affinity to Suc than fruit liquid, but their preference to fruit liquid was significantly higher compared to Glu and Fru. The average liquid intakes per animal (6 h) in fruit liquid-Suc-water, fruit liquid-Glu-water and fruit liquid-Fru-water combinations were 10.74, 10.40 and 11.36 mL, respectively. The overall intake of liquids went up when their combinations were provided compared to water alone. In another set of preference experiments, rats showed higher preference to seed compared to fruit pericarp and normal food. Water intake was high for rats provided with the fibrous pericarp compared to seeds. These feeding experiments established a clear relative affinity of rats towards ‘Suc-rich’ fruit liquid, seed and Suc itself [9]. *M*. *baccifera* fruits (sugary) along with other field supplements drive these rats into a reproductive fury. Rats and other mammals also have the capacity to attack the relatively harder pericarp and reach the seeds in mature fruits. Govindan and co-workers [9] demonstrated that fruit alone is not a complete food for the maintenance of normal growth and physiology in animals. On the contrary, the fruit supplemented with normal food showed maintenance of body weight, normal serum biochemical and hematological parameters and reduction in serum total cholesterol levels [9]. This is mimicking the bamboo field scenario where rodents consume *M*. *baccifera* fruits along with other grains and food items.

Laska described the preferences of squirrel monkeys and pigtail macaques for individual sugars as Suc > Fru > Glu > maltose > lactose and maltose *>* Suc *>* Fru *>* lactose *>* Glu, respectively [75, 76]. Numerous studies demonstrated that rats are attracted to the taste of sugars, and their order of sugar preferences at low and high molar concentrations are maltose > Suc > Glu = Fru and Suc > maltose > Glu = Fru, respectively [74, 77]. Sugars in the fruit liquid/seed and fibrous pericarp of *M*. *baccifera* allure rats, they overeat and multiply relatively quickly in short reproductive cycles. Hungry rats that binge on sugars, provoke a surge of dopamine, release dopamine and opioids into their brain, and are expected to develop an addictive potential leading to sugar (fruit) dependency in them [78–80]. Extra ordinary growth (number and size, as big as wild cats) of rats in *Muli* flowering seasons was reported from Mizoram and other NE Indian locations [15]. In NE India, rats were reported as the dominant predators. Rat population enhances on fruit feeding, and over time their numbers subside due to ‘unknown consequences’. But, we found monkeys as the major group of mammal predators (Fig 5C).

Studies found that wild boars (*S*. *scrofa*) prefer carbohydrates over water. The gustatory preferences of carbohydrates in pigs for Suc and Fru are identical, with the molar order of their effectiveness as Suc > Fru > maltose = lactose > Glu > galactose [81, 82]. *S*. *scrofa* attack on *M*. *baccifera* fruits was observed from 3^rd^-6^th^ week (Table 1 and Figs 3, 4 and 5C), when their Fru levels are moderate/high and Suc gradually elevated to the highest. Porcupines also show preference to palatable Suc solutions [83, 84], and not to Glu and Fru. Porcupine (*H*. *indica*) attacks were recorded on near mature and mature (5-6^th^ week) fruits, with relatively high contents of Suc (Table 1 and Figs 3, 4 and 5C). Palm civet (*P*. *hermaphrodites*), which occasionally visits (predates) fruits in the 6^th^ week (Table 1 and Fig 4), also has a preference to sweet (sugary) taste [85]. Debussche and Isenmann [86], in an extensive study, illustrated that mammals forsake protein rich fleshy fruits. The low protein content, high sugars (energy), amino acids, lipids, minerals, vitamins, flavonoids (antioxidants), phenolics (antioxidants) [9, 21], along with the morpho/visual features, cumulatively act as factors favoring mammal predation on *M*. *baccifera* fruits. The millipede (*S*. *colosseus*) consumes fruit fragments, acts as a primary decomposer and its taste (sugar) preferences are so far unknown.

Briefly, slugs, snails (molluscs) show preference to monosaccharides (Glu, Fru), and their attacks are in the early stages (1-3^rd^ weeks) of fruit development (Table 1 and Figs 3, 4 and 5C). Borers (arthropods) display an affinity to the Glu-Fru-Suc and their attack prolong from the second to 6^th^ (mature fruits) week (Table 1 and Figs 3, 4 and 5C). But, frugivorous mammals (rats, monkeys, porcupines, wild boars, palm civets) predating on near mature/mature fruits display Suc preference, and this trend is also established by other studies [84, 87]. Overall, sugars are making *M*. *baccifera* fruits an energy-rich diet for predators and they act as one of the factors invigorating rat multiplication. Polyamines play major roles in floral initiation, floral and fruit development, fruit ripening, senescence and several other growth processes in plants [88]. Therefore, detection of verbacine (a rare polyamine) in *M*. *baccifera* fruits is significant as they display parthenocarpy [29, our observations], and such fruits are known to express polyamine biosynthetic genes [89]. The specific roles of secondary metabolites (leaves, fruits: terpenoids, steroids, fatty acids, fatty acid derivatives, nor-isoprenoids, aldehydes, flavonoids and phenolic acids [21]) in defense, predation and fruit/seed dispersal are to be further explored. The nutritional components of fruits (amino acids, sugars, phenolics, fatty acids, minerals, vitamins, protein (low)) demonstrate that they are not complete food. Our conjecture is, *M*. *baccifera* fruit amino acids and sugars are the major factors promoting rat multiplication on fruit feeding. Sugars, other nutritional components and secondary metabolites promote a dynamic interaction between *Muli* fruits, which appear once in 48 years, and its predators.

## Conclusions

The study on *M*. *baccifera* flowering clumps, from February 2009 to August 2022, provided new insights into its reproductive biology and animal interactions. It underlines a common pattern in flowering, pollination, fruiting and death in all bamboos. The temporal and spatial separation of male and female stages demonstrated in this study are adaptations, especially in semelparous bamboos, for effective pollination and oversupply of fruits. Visitation pattern of bees, *A*. *cerana indica*, *T*. *iridipennis*, *H*. *taprabonae* and *B*. *cupulifera*, is similar to other studied bamboos in the region. Our observations suggest ‘wind and insect assisted pollination’ in *M*. *baccifera*; and compel us to ponder whether transition between wind and animal pollination [55] has occurred in bamboos? We record the highest ever fruit production in a bamboo clump as 456.67 Kg. *Good fruits*, *Immature fruits* and *Predated fruits* in the total fallen fruits (38371) of eight clumps were 43.80%, 18.09% and 38.11%, respectively. The predation rates are very high in short periods of highest fruit production, indicating the thrust for ‘high rewards’. Fruit predators include three mollusks (slug: *M*. *dussumieri*, snails: *C*. *bistrialis*, *Macrochlamys* sp.), three arthropods (borers: *A*. *grisella*, *B*. *germanica*; millipede: *S*. *colosseus*), five frugivorous mammals (monkeys: *M*. *radiata*, rats: *R*. *rattus*, porcupine: *H*. *indica*, wild boar: *S*. *scrofa*, palm civet: *P*. *hermaphrodites*). Two other mammals, rabbits (*L*. *nigricollis*) and deers (*A*. *axis*), are primarily seedling predators; but low incidences of fruit predation by them were also observed. In addition, seedling predation by *S*. *scrofa* was noted by us. While the millipede decomposed fruit remnants enriching soil around the clumps, the two ant species (*C*. *biroi*, *O*. *smaragdina*) and one mantis (*E*. *indica*) are protective in function. In NE India, rats were reported as the dominant predators, whereas in JNTBGRI Bambusetum monkeys were the major group of predators. All interactions help the plant establishing successfully in an introduced region. We also observed a paradigm shift in the pattern of predation, from animals to humans.

The fruit chemistry has a very significant role in predation, as fruits were consumed by predators based on their taste preferences to fruit sugars at various growth stages. Fruit liquid and pericarp are rich in Glu and Fru and low in Suc in early weeks (up to 21 days), and Suc levels enhance in late growth stages (4-6^th^ week). Mollusks (arthropods) are fond of monosaccharides (Glu, Fru) and they attack young fruits when the Glu and Fru levels are high. Borers (arthropods) attacked young (2^nd^ week) to mature fruits (6^th^ week), with mixed Glu-Fru-Suc profiles. Frugivorous mammals (rats, monkeys, pigs, wild boars, civets) preferred high Suc. Sugars, and not proteins which are popularly believed as causing enhanced predation and rat multiplication, but low in content [9], are making *M*. *baccifera* fruits an energy-rich diet which possibly vitalize the rat explosion induced by other metabolites. This study, thus, details the interactions of *M*. *baccifera*, native to NE India-Myanmar region, with the fauna of the southern Indian region aiding its successful establishment at the foothills of the Western Ghats. Our studies on nutritional properties, secondary metabolic profiles and data on fruit dynamics and floral/fruit-visitor/predator interactions will definitely augment our ongoing efforts towards unveiling the enigma associated with the mast flowering of *M*. *baccifera*.

## Supporting information

S1 File **S1 Table.** Life details of eight flowered *M*. *baccifera* clumps. **S2 Table.** Details of voucher specimens deposited at TBGT. **S3 Table.** Fruit production dynamics, predation in eight *M*. *baccifera* clumps. **S4 Table.** Flowering and fruiting durations of eight *M*. *baccifera* clumps. **S5 Table.** Summary of total fruits, good fruits, immature fruits, predated fruits in *M*. *baccifera*. **S6 Table.** One month of highest fruit production, predation pattern in *M*. *baccifera*. **S7 Table.** Fruit dynamics, predation pattern in *M*. *baccifera*. **S8 Table.** Duration of female, male stages and female-male interval in *M*. *baccifera*. **S9 Table.** Slug/snail predation in clump 58. **S10 Table.** Fruit predation breakup in clump 359 of *M*. *baccifera*. **S11 Table.** Borer larvae attack in clump 58 of *M*. *baccifera*. **S1 (A-D) Fig.** *M*. *baccifera* fruits at JNTBGRI Bambusetum. **S2 (A-B) Fig.** *A*. *cerana indica* on *M*. *baccifera* inflorescence. **S3 Fig.** *M*. *baccifera* male flower with bee, *H*. *taprabonae*. **S4. (A-B) Fig.** *M*. *baccifera*, bee activity, *T*. *iridipennis*. **S5. (A-B). Fig** Mantis, *E*. *indica* on *M*. *baccifera* inflorescence. **S6. (A-B) Fig.** Ants, *C*. *biroi* on fruits and internodes. **S7. (A-B) Fig.** Ants, *O*. *smaragdina* on *M*. *baccifera*. **S8. (A-F) Fig.** *M*. *baccifera*, slug attack, *M*. *dussumieri* on young fruits. **S9. (A-C) Fig.** *M*. *baccifera*, snail *C*. *bistrialis* attacking young fruits. **S10. (A-D) Fig.** *M*. *baccifera*, snail attack, *Macrochlamys* sp. on young fruits. **S11. (A-D) Fig.** Larvae of *A*. *grisella* (lesser wax moth) on *M*. *baccifera* fruits. **S12. (A-B) Fig.** *B*. *germanica* (German cockroach) larva inside *M*. *baccifera* fruit. **S13 (A-B) Fig.** *R*. *rattus* predation, *M*. *baccifera* fruits. **S14. (A-B) Fig.** *M*. *baccifera* seedling predation, damage by *S*. *scrofa*. **S15. (A-B) Fig.** *S*. *scrofa*, hoof marks and soil rooting characteristics. **S16 (A-B) Fig.** Quills of porcupine, *H*. *indica*, below *M*. *baccifera* clump. **S17 (A-B) Fig.** Bonnet macaque, *M*. *radiata*, eating *M*. *baccifera* fruits. **S18 (A-B) Fig.** Millipede, *S*. *colosseus*.
(DOCX)
