# Correction: Flower-fruit dynamics, visitor-predator patterns and chemical preferences in the tropical bamboo, *Melocanna baccifera*

**DOI:** 10.1371/journal.pone.0306685

**Published:** 2024-07-05

**Authors:** The PLOS One Staff

The fourth author’s initials appear incorrectly in the citation. The correct citation is: Koshy KC, Gopakumar B, Sebastian A, Nair S A, Johnson AJ, Govindan B, et al. (2022) Flower-fruit dynamics, visitor-predator patterns and chemical preferences in the tropical bamboo, Melocanna baccifera. PLoS ONE 17(11): e0277341. https://doi.org/10.1371/journal.pone.0277341.

The publisher apologizes for the error.
