# Supplementary material for: Flower-fruit dynamics, visitor-predator patterns and chemical preferences in the tropical bamboo, Melocanna baccifera
Source: PLoS One. 2022 Nov 16;17(11):e0277341. doi: 10.1371/journal.pone.0277341 (PMC9668177; doi:10.1371/journal.pone.0277341)
Supplement: S1 File — S1 Table. Life details of eight flowered M. baccifera clumps. S2 Table. Details of voucher specimens deposited at TBGT. S3 Table. Fruit production dynamics, predation in eight M. baccifera clumps. S4 Table. Flowering and fruiting durations of eight M. baccifera clumps. S5 Table. Summary of total fruits, good fruits, immature fruits, predated fruits in M. baccifera. S6 Table. One month of highest fruit production, predation pattern in M. baccifera. S7 Table. Fruit dynamics, predation pattern in M. baccifera. S8 Table. Duration of female, male stages and female-male interval in M. baccifera. S9 Table. Slug/snail predation in clump 58. S10 Table. Fruit predation breakup in clump 359 of M. baccifera. S11 Table. Borer larvae attack in clump 58 of M. baccifera. S1 (A-D) Fig. M. baccifera fruits at JNTBGRI Bambusetum. S2 (A-B) Fig. A. cerana indica on M. baccifera inflorescence. S3 Fig. M. baccifera male flower with bee, H. taprabonae. S4. (A-B) Fig. M. baccifera, bee activity, T. iridipennis. S5. (A-B). Fig Mantis, E. indica on M. baccifera inflorescence. S6. (A-B) Fig. Ants, C. biroi on fruits and internodes. S7. (A-B) Fig. Ants, O. smaragdina on M. baccifera. S8. (A-F) Fig. M. baccifera, slug attack, M. dussumieri on young fruits. S9. (A-C) Fig. M. baccifera, snail C. bistrialis attacking young fruits. S10. (A-D) Fig. M. baccifera, snail attack, Macrochlamys sp. on young fruits. S11. (A-D) Fig. Larvae of A. grisella (lesser wax moth) on M. baccifera fruits. S12. (A-B) Fig. B. germanica (German cockroach) larva inside M. baccifera fruit. S13 (A-B) Fig. R. rattus predation, M. baccifera fruits. S14. (A-B) Fig. M. baccifera seedling predation, damage by S. scrofa. S15. (A-B) Fig. S. scrofa, hoof marks and soil rooting characteristics. S16 (A-B) Fig. Quills of porcupine, H. indica, below M. baccifera clump. S17 (A-B) Fig. Bonnet macaque, M. radiata, eating M. baccifera fruits. S18 (A-B) Fig. Millipede, S. colosseus. (DOCX) [file pone.0277341.s001.docx]

**Supporting information**

**Full title: Flower-fruit dynamics, visitor-predator patterns and chemical preferences in the tropical bamboo, *Melocanna baccifera***

**Short title: Fruit dynamics, predator patterns in *Melocanna baccifera***

**Konnath Chacko Koshy^1,^*, Bhaskaran Gopakumar^1^, Antony Sebastian^1^, Ajikumaran Nair S^2^, Anil John Johnson^2^, Balaji Govindan^2^, Sabulal Baby^2^***

^1^Plant Genetic Resources Division, Jawaharlal Nehru Tropical Botanic Garden and Research Institute, Pacha-Palode, Thiruvananthapuram 695 562 Kerala, India

^2^Phytochemistry and Phytopharmacology Division, Jawaharlal Nehru Tropical Botanic Garden and Research Institute, Pacha-Palode, Thiruvananthapuram 695 562 Kerala, India

*Corresponding author

E-mail: sabulal@jntbgri.res.in, sabulal@gmail.com; **Orcid:** 0000-0002-4337-9431 (SB)

E-mail: koshykc1@gmail.com, **Orcid:** 0000-0002-2530-0996 (KCK)

**Abstract**

Mast seeding and associated events in *Melocanna baccifera*, the largest fruit producing bamboo, is an enigma. So far there are no comprehensive accounts on its flowering phenology, fruiting dynamics and animal interactions. In this study, spanning over 13 years (2009 to 2022), we observed eight *M. baccifera* clumps in JNTBGRI Bambusetum from flowering initiation, fruiting to eventual death. Flowering phenology, floral characteristics, floret types, breeding system, bee visitation, pollination, fruit production and predators were recorded; predation patterns were correlated with fruit chemistry. Flowering duration of clumps ranged from 20 (March 2009 - October 2010) to 120 (September 2012 - August 2022) months. Bisexual florets are dichogamous and protogynous; and female duration (22-72 h) is many times higher than male duration (2-6 h). The highest ever fruit production for an individual bamboo clump (456.67 Kg) was recorded. Of the total fallen fruits (38371), 38.11% were predated, 43.80% good fruits (no predator hits) and 18.09% immature fruits. A positive correlation between reward (fruits) *versus* predation was observed, especially in short intervals of high fruit production. Pollen predators (*Apis cerana indica*, *Halictus taprabonae*, *Braunsapis cupulifera*, *Trigona iridipennis*), fruit predators, ranging from arthropods to mammals, *viz*., millipede (*Spinotarsus colosseus*), slug (*Mariaella dussumieri*), snails (*Cryptozona bistrialis*, *Macrochlamys* sp.), borers (*Achroia grisella*, *Blattella germanica*), mammals (monkeys *Macaca radiata*, rats *Rattus rattus*, porcupine *Hystrix indica*, wild boar *Sus scrofa*, palm civet *Paradoxurus hermaphroditus*), seedling predators (rabbit *Lepus nigricollis*, deer *Axis axis*), and insect/pest predators (ants *Crematogaster biroi*, *Oecophylla smaragdina*, mantis *Euchomenella indica*) were identified. Fruit predation is linked to its age and chemistry. Apart from new insights on flowering phenology, breeding system, pollination and fruiting dynamics, this study demonstrates the vibrant interaction between *M. baccifera* flowers/fruits and visitors/predators, and provides significant leads towards elucidating the cause of rat multiplication and other events associated with its gregarious flowering.

**Table S1.** Life details of eight flowered *M. baccifera* clumps.

| Clump No. | Collection number | Collection date | Original habitat | Planting date | Current habitat  JNTBGRI Bambusetum |
| --- | --- | --- | --- | --- | --- |
| 58 | 4330 | 15 Sep 1988 | FRI, Dehradun,  Uttarakhand | 20 Sep 1988 | Under partial shade, undulated plane |
| 359 | 28606 | 18 Mar 1996 | Tamenglong, Tupul, Manipur | 22 Mar 1996 | Under partial shade, slight slope |
| 365 | 28666 | 28 Mar 1996 | Senapati, Saikul Hills, Manipur | 30 Mar 1996 | Under partial shade, slight slope |
| 394 | 28607 | 17 Mar 1996 | Lukram Lerik, Imphal, Manipur | 18 Aug 1996 | Open, steep slope |
| 395 | 28607 | 17 Mar 1996 | Lukram Lerik, Imphal, Manipur | 18 Aug 1996 | Open, steep slope |
| 403 | 28648 | 26 Mar 1996 | Chandel, Way to Moreh, Manipur | 18 Aug 1996 | Under shade, steep slope |
| 404 | 28649 | 26 Mar 1996 | Chandel, Way to Moreh, Manipur | 18 Aug 1996 | Under deeper shade, steep slope |
| 405 | 28666 | 28 Mar 1996 | Senapati, Saikul, Manipur | 18 Aug 1996 | Under partial shade, slight slope |

**Table. S2.** Details of voucher specimens deposited at TBGT.

| Sl. No. | Species | Family | Collection number/Photographs | | |
| --- | --- | --- | --- | --- | --- |
|  |  |  | Herbarium specimens | Spirit collections | Photographs |
| 1 | *Melocanna baccifera* (Roxb.) Kurz | Poaceae (subfamily Bambusoideae) | India, Kerala, Bambusetum, JNTBGRI, N 08˚45.262-430’ E 077˚ 01.429-583’  Clump **58**: 23.10.2009, *B. Gopakumar 66813*; 18.5.2011 *B. Gopakumar 66895.*  Clump **359**: 13. 7. 2007, *B. Gopakumar 47940*; 18. 11. 2008, *B. Gopakumar 47974*.  Clump **365**: 25. 11. 2009, *A. Sebastian 66645.*  Clump **366**: 25. 11. 2009, *A. Sebastian 66646*.  Clump **395**: 18. 11. 2009, *B. Gopakumar 66636.*  Clump **403**: 2. 11. 2009, *A. Sebastian 66621*. Sikkim**:** 22.2.2011, *K. C. Koshy 66689*. | Clump **359:** 13. 7.2007, *B. Gopakumar 47940*; 18. 11. 2008, *B. Gopakumar 47974*; 22. 4. 2009, *B. Gopakumar 48000*; *B. Gopakumar s.n.*  Clump **394**: 27. 6. 2006, *K. C. Koshy 54179*. | ✓ |
| 2 | *Apis cerana indica* Smith | Bee species Insecta: Hymenoptera: Apidae | - | 26.04.2011 *B. Gopakumar,* 70503; 20.05.2011 *B. Gopakumar* 70507 | ✓ |
| 3 | *Braunsapis cupulifera* Vachal | Apidae | - | 27.04.2011,  *B. Gopakumar,* 70504b | ✓ |
| 4 | *Halictus taprabonae* Cameron | Halictidae | - | 27.04.2011, *B. Gopakumar,* 70504a. | ✓ |
| 5 | *Trigona iridipennis* Smith | Apidae | - | 27.04.2011,  *B. Gopakumar* 70505 | ✓ |
| 6 | *Achroia grisella* Fb. | Arthropods  Lepidoptera: Galleridae | - | 20.04.2011,  *B. Gopakumar*  66888;  25.04.2011, *B. Gopakumar* 66890 | ✓ |
| 7 | *Blattella germanica* L. | Ectobiidae | - | 13.09.2010*, B. Gopakumar*  66855 | ✓ |
| 8 | *Euchomenella indica* Ghate & Mukherjee | Mantis  Mantidae | - | *17.09.2010, B. Gopakumar*, 66857 | ✓ |
| 9 | *Cryptozona bistrialis* H.H. Beck | Snail  Ariophantidae | - | *08.09.2010, B. Gopakumar,*  *66852; 28.06.2011, B. Gopakumar, 66900* | ✓ |
| 10 | *Mariaella dussumieri* Gray | Slug  Ariophantidae | - | *08.09.2010 B. Gopakumar 66853; 28.06.2011 B. Gopakumar 66899; 03.06.2011 B. Gopakumar, 70511* |  |
| 11 | *Macrochlamys* sp. | Snail  Ariophantidae | - | *08.09.2010, B. Gopakumar, 66854* | ✓ |
| 12 | *Crematogaster biroi* Mayr. | Ant  Formicidae | - | 03.08.2011, *B. Gopakumar* 64201 | ✓ |
| 13 | *Oecophylla smaragdina* Fab. | Ant  Formicidae | - | *B. Gopakumar* | ✓ |
| 14 | *Hystrix indica* Kerr | Porcupines  Hystricidae | - | - | ✓ |
| 15 | *Macaca radiata* É. Geoffroy | Monkeys (Bonnet macaque)  Cercopithecidae | - | - | ✓ |
| 16 | *Rattus rattus* L. | Rats  Muridae | - | - | ✓ |
| 17 | *Sus scrofa* L. | Wild boar  Suidae | - | - | ✓ |
| 18 | *Paradoxurus hermaphroditus* Pallas | Asian Palm civet  Viverridae | - | - | ✓ |
| 19 | *Axis axis* Erxleben | Spotted deer  Cervidae | - | - | ✓ |
| 20 | *Lepus nigricollis* F. Cuvier | Indian hare  Leporidae | - | - | ✓ |
| 21 | *Spinotarsus colosseus* Attems | Millipede  Odontopygidae | - | - | ✓ |

**Table S3.** Fruit production dynamics, predation in eight *M. baccifera* clumps.

**Monthly fruit production & predation**

*Procedure***:** All fruits shed from eight clumps (58, 359, 365, 394, 395, 403, 404, 405) were gathered daily. From each of these, the mature fruits, devoid of animal bites, were numbered and recorded as ‘*Good fruits*’ (GF). Counts of underdeveloped were recorded as ‘*Immature fruits*’ (IF). Animal eaten remains were recorded as *‘Predated fruits’* (PF). All daily counts of one month were added to get the month’s total.

| **[A] Clump 58** | | | | | |
| --- | --- | --- | --- | --- | --- |
| Sl. No. | Month/Year | Total fruits (TF) | Good fruits (GF) | Immature fruits (IF) | Predated fruits (PF) |
|  | April 2009 | 0 | 0 | 0 | 0 |
|  | May 2009 | 4 | 4 | 0 | 0 |
|  | June 2009 | 18 | 16 | 1 | 1 |
|  | July 2009 | 37 | 22 | 8 | 7 |
|  | August 2009 | 8 | 1 | 4 | 3 |
|  | September 2009 | 0 | 0 | 0 | 0 |
|  | October 2009 | 1 | 0 | 1 | 0 |
|  | November 2009 | 0 | 0 | 0 | 0 |
|  | December 2009 | 0 **(68) (9 months)^¥^** | 0 **(43)** | 0 **(14)** | 0 **(11)** |
|  | January 2010 | 2 | 0 | 1 | 1 |
|  | February 2010 | 1 | 0 | 1 | 0 |
|  | March 2010 | 7 | 0 | 2 | 5 |
|  | April 2010 | 13 | 8 | 2 | 3 |
|  | May 2010 | 20 | 14 | 1 | 5 |
|  | June 2010 | 20 | 13 | 0 | 7 |
|  | July 2010 | 21 | 9 | 8 | 4 |
|  | August 2010 | 33 | 28 | 2 | 3 |
|  | September 2010 | 46 | 38 | 3 | 5 |
|  | October 2010 | 63 | 55 | 4 | 4 |
|  | November 2010 | 21 | 16 | 3 | 2 |
|  | December 2010 | 11 **(258) (12 months)** | 10 **(191)** | 1 **(28)** | 0 **(39)** |
|  | January 2011 | 7 | 7 | 0 | 0 |
|  | February 2011 | 8 | 8 | 0 | 0 |
|  | March 2011 | 20 | 10 | 8 | 2 |
|  | April 2011 | 250 | 186 | 22 | 42 |
|  | May 2011 | 1129 | 872 | 140 | 117 |
|  | June 2011 | 1118 | 708 | 199 | 211 |
|  | July 2011 | 778 | 456 | 127 | 195 |
|  | August 2011 | 431 | 298 | 52 | 81 |
|  | September 2011 | 294 | 204 | 43 | 47 |
|  | October 2011 | 59 | 44 | 3 | 12 |
|  | November 2011 | 24 | 11 | 0 | 13 |
|  | December 2011 | 15 **(4133) (12 months)** | 8 **(2812)** | 3 **(597)** | 4 **(724)** |
|  | January 2012 | 34 | 14 | 5 | 15 |
|  | February 2012 | 36 | 14 | 4 | 18 |
|  | March 2012 | 59 | 39 | 9 | 11 |
|  | April 2012 | 292 | 217 | 40 | 35 |
|  | May 2012 | 327 | 196 | 63 | 68 |
|  | June 2012 | 222 | 110 | 62 | 50 |
|  | July 2012 | 98 | 84 | 9 | 5 |
|  | August 2012 | 74 | 59 | 9 | 6 |
|  | September 2012 | 14 | 14 | 0 | 0 |
|  | October 2012 | 17 | 9 | 0 | 8 |
|  | November 2012 | 4 | 1 | 1 | 2 |
|  | December 2012 | 0 **(1177) (12 months)** | 0 **(757)** | 0 **(202)** | 0 **(218)** |
|  | January 2013 | 0 | 0 | 0 | 0 |
|  | February 2013 | 0 | 0 | 0 | 0 |
|  | March 2013 | 5 | 2 | 3 | 0 |
|  | April 2013 | 49 | 45 | 4 | 0 |
|  | May 2013 | 24 | 20 | 4 | 0 |
|  | June 2013 | 12 | 7 | 2 | 3 |
|  | July 2013 | 11 | 6 | 2 | 3 |
|  | August 2013 | 2 | 0 | 2 | 0 |
|  | September 2013 | 1 | 0 | 1 | 0 |
|  | October 2013 | 0 | 0 | 0 | 0 |
|  | November 2013 | 0 | 0 | 0 | 0 |
|  | December 2013 | 1 **(105) (12 months)** | 1 **(81)** | 0 **(18)** | 0 **(6)** |
|  | January 2014 | 0 | 0 | 0 | 0 |
|  | February 2014 | 0 | 0 | 0 | 0 |
|  | March 2014 | 1 | 0 | 1 | 0 |
|  | April 2014 | 1 | 0 | 1 | 0 |
|  | May 2014 | 6 | 4 | 2 | 0 |
|  | June 2014 | 2 | 2 | 0 | 0 |
|  | July 2014 | 1 | 0 | 1 | 0 |
|  | August 2014 | 0 | 0 | 0 | 0 |
|  | September 2014 | 0 | 0 | 0 | 0 |
|  | October 2014 | 0 | 0 | 0 | 0 |
|  | November 2014 | 0 | 0 | 0 | 0 |
|  | December 2014 | 0 **(11) (12 months)** | 0 **(6)** | 0 **(5)** | 0 **(0)** |
|  | January 2015  (Clump death) | 0 | 0 | 0 | 0 |
| **Total fruits (TF)** | | **5752** | **3890** | **864** | **998** |
| Total fruits (TF) produced by accession 58: **284.44 Kg** (average weight of fruit 49.45 ± 25.75 g, n = 500). | | | | | |

| **[B] Clump 359** | | | | | |
| --- | --- | --- | --- | --- | --- |
| Sl. No. | Month/Year | Total fruits (TF) | Good fruits (GF) | Immature fruits (IF) | Predated fruits (PF) |
|  | February 2009 | 0 | 0 | 0 | 0 |
|  | March 2009 | 2 | 2 | 0 | 0 |
|  | April 2009 | 10 | 10 | 0 | 0 |
|  | May 2009 | 200 | 187 | 13 | 0 |
|  | June 2009 | 301 | 198 | 27 | 76 |
|  | July 2009 | 414 | 270 | 82 | 62 |
|  | August 2009 | 396 | 102 | 59 | 235 |
|  | September 2009 | 252 | 34 | 77 | 141 |
|  | October 2009 | 11 | 5 | 5 | 1 |
|  | November 2009 | 34 | 13 | 13 | 8 |
|  | December 2009 | 81 **(1701) (11 months)^¥^** | 13 **(834)** | 38 **(314)** | 30 **(553)** |
|  | January 2010 | 27 | 1 | 16 | 10 |
|  | February 2010 | 12 | 0 | 12 | 0 |
|  | March 2010 | 38 | 20 | 6 | 12 |
|  | April 2010 | 1464 | 267 | 303 | 894 |
|  | May 2010 | 778 | 290 | 234 | 254 |
|  | June 2010 | 698 | 202 | 233 | 263 |
|  | July 2010 | 274 | 61 | 88 | 125 |
|  | August 2010 | 70 | 23 | 28 | 19 |
|  | September 2010 | 3 | 0 | 2 | 1 |
|  | October 2010 | 11 | 3 | 3 | 5 |
|  | November 2010 | 2 | 0 | 2 | 0 |
|  | December 2010 | 4 **(3381) (12 months)** | 0 **(867)** | 4 **(931)** | 0 **(1583)** |
|  | January 2011 | 3 | 0 | 3 | 0 |
|  | February 2011 | 3 | 0 | 3 | 0 |
|  | March 2011 | 2 | 0 | 2 | 0 |
|  | April 2011 | 7 | 6 | 1 | 0 |
|  | May 2011 | 819 | 236 | 154 | 429 |
|  | June 2011 | 1102 | 383 | 209 | 510 |
|  | July 2011 | 468 | 214 | 82 | 172 |
|  | August 2011 | 382 | 211 | 73 | 98 |
|  | September 2011 | 247 | 83 | 52 | 112 |
|  | October 2011 | 97 | 60 | 23 | 14 |
|  | November 2011 | 10 | 3 | 1 | 6 |
|  | December 2011 | 12 **(3152) (12 months)** | 4 **(1200)** | 4 **(607)** | 4 **(1345)** |
|  | January 2012 | 28 | 10 | 4 | 14 |
|  | February 2012 | 6 | 3 | 0 | 3 |
|  | March 2012 | 7 | 4 | 1 | 2 |
|  | April 2012 | 281 | 209 | 54 | 18 |
|  | May 2012 | 168 | 130 | 18 | 20 |
|  | June 2012 | 100 | 75 | 10 | 15 |
|  | July 2012 | 38 | 31 | 3 | 4 |
|  | August 2012 | 22 | 16 | 2 | 4 |
|  | September 2012 | 2 | 2 | 0 | 0 |
|  | October 2012 | 18 | 12 | 2 | 4 |
|  | November 2012 | 3 | 1 | 1 | 1 |
|  | December 2012 | 0 **(673) (12 months)** | 0 **(493)** | 0 **(95)** | 0 **(85)** |
|  | January 2013 | 4 | 3 | 1 | 0 |
|  | February 2013 | 1 | 0 | 1 | 0 |
|  | March 2013 | 31 | 29 | 2 | 0 |
|  | April 2013 | 90 | 73 | 6 | 11 |
|  | May 2013 | 11 | 10 | 1 | 0 |
|  | June 2013 | 2 | 1 | 0 | 1 |
|  | July 2013 | 7 | 7 | 0 | 0 |
|  | August 2013 | 6 | 5 | 0 | 1 |
|  | September 2013 | 0 | 0 | 0 | 0 |
|  | October 2013 | 5 | 3 | 2 | 0 |
|  | November 2013 | 30 | 20 | 4 | 6 |
|  | December 2013 | 19 **(206) (12 months)** | 16 **(167)** | 2 **(19)** | 1 **(20)** |
|  | January 2014 | 3 | 1 | 2 | 0 |
|  | February 2014 | 0 | 0 | 0 | 0 |
|  | March 2014 | 4 | 3 | 1 | 0 |
|  | April 2014 | 2 | 0 | 2 | 0 |
|  | May 2014 | 6 | 0 | 2 | 4 |
|  | June 2014 | 79 | 59 | 5 | 15 |
|  | July 2014 | 12 | 9 | 1 | 2 |
|  | August 2014 | 1 | 0 | 1 | 0 |
|  | September 2014 | 3 | 0 | 1 | 2 |
|  | October 2014 | 2 | 0 | 1 | 1 |
|  | November 2014 | 4 | 3 | 1 | 0 |
|  | December 2014 | 0 **(116) (12 months)** | 0 **(75)** | 0 **(17)** | 0 **(24)** |
|  | January 2015 | 2 | 1 | 1 | 0 |
|  | February 2015 | 0 | 0 | 0 | 0 |
|  | March 2015 | 2 | 1 | 1 | 0 |
|  | April 2015 | 2 **(6) (4 months)** | 2 **(4)** | 0 **(2)** | 0 **(0)** |
|  | May 2015  (Clump death) | 0 | 0 | 0 | 0 |
| **Total fruits (TF)** | | **9235** | **3640** | **1985** | **3610** |
| Total fruits (TF) produced by accession 359: **456.67 Kg** (average weight of fruit 49.45 ± 25.75 g, n = 500). | | | | | |

| **[C] Clump 365** | | | | | |
| --- | --- | --- | --- | --- | --- |
| Sl. No. | Month/year | Total fruits (TF) | Good fruits (GF) | Immature fruits (IF) | Predated fruits (PF) |
|  | January 2012 | 0 | 0 | 0 | 0 |
|  | February 2012 | 5 | 3 | 0 | 2 |
|  | March 2012 | 65 | 5 | 10 | 50 |
|  | April 2012 | 305 | 207 | 52 | 46 |
|  | May 2012 | 316 | 242 | 40 | 34 |
|  | June 2012 | 208 | 154 | 14 | 40 |
|  | July 2012 | 127 | 112 | 8 | 7 |
|  | August 2012 | 13 | 8 | 2 | 3 |
|  | September 2012 | 1 | 0 | 1 | 0 |
|  | October 2012 | 3 | 3 | 0 | 0 |
|  | November 2012 | 3 | 1 | 1 | 1 |
|  | December 2012 | 0 **(1046) (12 months)^¥^** | 0 **(735)** | 0 **(128)** | 0 **(183)** |
|  | January 2013 | 0 | 0 | 0 | 0 |
|  | February 2013 | 0 | 0 | 0 | 0 |
|  | March 2013 | 2 | 0 | 2 | 0 |
|  | April 2013 | 329 | 202 | 41 | 86 |
|  | May 2013 | 193 | 170 | 18 | 5 |
|  | June 2013 | 8 | 6 | 0 | 2 |
|  | July 2013 | 78 | 53 | 10 | 15 |
|  | August 2013 | 14 | 10 | 3 | 1 |
|  | September 2013 | 2 | 0 | 1 | 1 |
|  | October 2013 | 7 | 5 | 2 | 0 |
|  | November 2013 | 4 | 2 | 2 | 0 |
|  | December 2013 | 5 **(642) (12 months)** | 1 **(449)** | 2 **(81)** | 2 **(112)** |
|  | January 2014 | 5 | 3 | 2 | 0 |
|  | February 2014 | 9 | 3 | 4 | 2 |
|  | March 2014 | 15 | 5 | 10 | 0 |
|  | April 2014 | 33 | 25 | 3 | 5 |
|  | May 2014 | 30 | 20 | 4 | 6 |
|  | June 2014 | 17 | 8 | 5 | 4 |
|  | July 2014 | 10 | 4 | 4 | 2 |
|  | August 2014 | 8 | 3 | 4 | 1 |
|  | September 2014 | 9 | 3 | 2 | 4 |
|  | October 2014 | 5 | 1 | 4 | 0 |
|  | November 2014 | 2 | 0 | 2 | 0 |
|  | December 2014 | 1 **(144) (12 months)** | 0 **(75)** | 1 **(45)** | 0 **(24)** |
|  | January 2015 | 0 | 0 | 0 | 0 |
|  | February 2015  (Clump death) | 0 | 0 | 0 | 0 |
| **Total fruits (TF)** | | **1832** | **1259** | **254** | **319** |
| Total fruits (TF) produced by accession 365: **90.59 Kg** (average weight of fruit 49.45 ± 25.75 g, n = 500). | | | | | |

| **[D] Clump 394** | | | | | |
| --- | --- | --- | --- | --- | --- |
| Sl. No. | Month/year | Total fruits (TF) | Good fruits (GF) | Immature fruits (IF) | Predated fruits (PF) |
|  | February 2009 | 0 | 0 | 0 | 0 |
|  | March 2009 | 2 | 2 | 0 | 0 |
|  | April 2009 | 75 | 58 | 5 | 12 |
|  | May 2009 | 420 | 360 | 15 | 45 |
|  | June 2009 | 864 | 735 | 40 | 89 |
|  | July 2009 | 727 | 452 | 170 | 105 |
|  | August 2009 | 833 | 195 | 174 | 464 |
|  | September 2009 | 419 | 53 | 151 | 215 |
|  | October 2009 | 4 | 0 | 4 | 0 |
|  | November 2009 | 14 | 6 | 5 | 3 |
|  | December 2009 | 27 **(3385) (10 months)^¥^** | 6 **(1867)** | 11 **(575)** | 10 **(943)** |
|  | January 2010 | 9 | 3 | 1 | 5 |
|  | February 2010 | 4 | 2 | 2 | 0 |
|  | March 2010 | 43 | 26 | 4 | 13 |
|  | April 2010 | 3601 | 414 | 716 | 2471 |
|  | May 2010 | 1275 | 376 | 279 | 620 |
|  | June 2010 | 533 | 189 | 140 | 204 |
|  | July 2010 | 163 | 55 | 47 | 61 |
|  | August 2010 | 39 | 5 | 13 | 21 |
|  | September 2010 | 3 | 1 | 2 | 0 |
|  | October 2010 | 5 **(5675) (10 months)** | 2 **(1073)** | 2 **(1206)** | 1 **(3396)** |
|  | November 2010  (Clump death) | 0 | 0 | 0 | 0 |
| **Total fruits (TF)** | | **9060** | **2940** | **1781** | **4339** |
| Total fruits (TF) produced by accession 394: **448.02 Kg** (average weight of fruit 49.45 ± 25.75 g, n = 500). | | | | | |

| **[E] Clump 395** | | | | | |
| --- | --- | --- | --- | --- | --- |
| Sl. No. | Month/year | Total fruits (TF) | Good fruits (GF) | Immature fruits (IF) | Predated fruits (PF) |
|  | March 2009 | 0 | 0 | 0 | 0 |
|  | April 2009 | 2 | 1 | 1 | 0 |
|  | May 2009 | 35 | 25 | 5 | 5 |
|  | June 2009 | 138 | 106 | 14 | 18 |
|  | July 2009 | 468 | 408 | 28 | 32 |
|  | August 2009 | 398 | 95 | 65 | 238 |
|  | September 2009 | 131 | 17 | 54 | 60 |
|  | October 2009 | 38 | 10 | 12 | 16 |
|  | November 2009 | 12 | 8 | 2 | 2 |
|  | December 2009 | 2 **(1224) (9 months)^¥^** | 2 **(672)** | 0 **(181)** | 0 **(371)** |
|  | January 2010 | 3 | 0 | 2 | 1 |
|  | February 2010 | 0 | 0 | 0 | 0 |
|  | March 2010 | 32 | 12 | 15 | 5 |
|  | April 2010 | 3230 | 280 | 605 | 2345 |
|  | May 2010 | 945 | 289 | 167 | 489 |
|  | June 2010 | 648 | 199 | 206 | 243 |
|  | July 2010 | 299 | 68 | 82 | 149 |
|  | August 2010 | 54 | 13 | 20 | 21 |
|  | September 2010 | 1 | 0 | 0 | 1 |
|  | October 2010  (Clump death) | 4 **(5216) (10 months)** | 1 **(862)** | 0 **(1097)** | 3 **(3257)** |
| **Total fruits (TF)** | | **6440** | **1534** | **1278** | **3628** |
| Total fruits (TF) produced by accession 395: **318.46 Kg** (average weight of fruit 49.45 ± 25.75 g, n = 500). | | | | | |

| **[F] Clump 403** | | | | | | |
| --- | --- | --- | --- | --- | --- | --- |
| Sl. No. | Month/year | Total fruits (TF) | Good fruits (GF) | Immature fruits (IF) | Predated fruits (PF) |  |
|  | March 2011 | 0 | 0 | 0 | 0 |  |
|  | April 2011 | 22 | 12 | 2 | 8 |  |
|  | May 2011 | 292 | 105 | 39 | 148 |  |
|  | June 2011 | 1488 | 827 | 151 | 510 |  |
|  | July 2011 | 469 | 246 | 66 | 157 |  |
|  | August 2011 | 230 | 101 | 29 | 100 |  |
|  | September 2011 | 42 | 19 | 2 | 21 |  |
|  | October 2011 | 32 | 27 | 1 | 4 |  |
|  | November 2011 | 9 | 6 | 1 | 2 |  |
|  | December 2011 | 22 **(2606) (10 months) ^¥^** | 11 **(1354)** | 3 **(294)** | 6 **(956)** |  |
|  | January 2012 | 13 | 4 | 1 | 8 |  |
|  | February 2012 | 9 | 2 | 1 | 6 |  |
|  | March 2012 | 250 | 103 | 32 | 118 |  |
|  | April 2012 | 338 | 239 | 58 | 41 |  |
|  | May 2012 | 242 | 180 | 24 | 38 |  |
|  | June 2012 | 130 | 89 | 16 | 25 |  |
|  | July 2012 | 77 | 58 | 10 | 9 |  |
|  | August 2012 | 1 | 1 | 0 | 0 |  |
|  | September 2012 | 1 | 0 | 1 | 0 |  |
|  | October 2012 | 3 | 1 | 2 | 0 |  |
|  | November 2012 | 1 | 0 | 1 | 0 |  |
|  | December 2012 | 0 **(1065) (12 months)** | 0 **(677)** | 0 **(146)** | 0 **(245)** |  |
|  | January 2013 | 0 | 0 | 0 | 0 |  |
|  | February 2013 | 0 | 0 | 0 | 0 |  |
|  | March 2013 | 3 | 0 | 3 | 0 |  |
|  | April 2013 | 132 | 105 | 11 | 16 |  |
|  | May 2013 | 32 | 25 | 4 | 3 |  |
|  | June 2013 | 20 | 13 | 2 | 5 |  |
|  | July 2013 | 38 | 31 | 1 | 6 |  |
|  | August 2013 | 10 | 8 | 0 | 2 |  |
|  | September 2013 | 3 | 1 | 2 | 0 |  |
|  | October 2013 | 3 | 2 | 1 | 0 |  |
|  | November 2013 | 1 | 0 | 1 | 0 |  |
|  | December 2013 | 2 **(244) (12 months)** | 2 **(187)** | 0 **(25)** | 0 **(32)** |  |
|  | January 2014 | 8 | 5 | 2 | 1 |  |
|  | February 2014 | 20 | 16 | 2 | 2 |  |
|  | March 2014 | 48 | 44 | 3 | 1 |  |
|  | April 2014 | 170 | 157 | 8 | 5 |  |
|  | May 2014 | 112 | 99 | 5 | 8 |  |
|  | June 2014 | 54 | 46 | 2 | 6 |  |
|  | July 2014 | 25 | 13 | 2 | 10 |  |
|  | August 2014 | 21 | 7 | 5 | 9 |  |
|  | September 2014 | 1 | 0 | 1 | 0 |  |
|  | October 2014 | 0 | 0 | 0 | 0 |  |
|  | November 2014 | 1 | 1 | 0 | 0 |  |
|  | December 2014 | 0 **(460) (12 months)** | 0 **(388)** | 0 **(30)** | 0 **(42)** |  |
|  | January 2015 | 0 | 0 | 0 | 0 |  |
|  | February 2015 | 0 | 0 | 0 | 0 |  |
|  | March 2015 | 6 | 4 | 2 | 0 |  |
|  | April 2015 | 4 | 2 | 2 | 0 |  |
|  | May 2015 | 2 **(12) (5 months)** | 1 **(7)** | 1 **(5)** | 0 **(0)** |  |
|  | June 2015 | 0 | 0 | 0 | 0 |  |
|  | July 2015 | 0 | 0 | 0 | 0 |  |
|  | August 2015  (Clump death) | 0 | 0 | 0 | 0 |  |
| **Total fruits (TF)** | | **4387** | **2613** | **500** | **1275** |  |
| Total fruits (TF) produced by accession 403: **216.94 Kg** (average weight of fruit 49.45 ± 25.75 g, n = 500). | | | | | |  |

| **[G] Clump 404** | | | | | |
| --- | --- | --- | --- | --- | --- |
| Sl. No. | Month/year | Total fruits (TF) | Good fruits (GF) | Immature fruits (IF) | Predated fruits (PF) |
|  | September 2012 | 0 | 0 | 0 | 0 |
|  | October 2012 | 2 | 2 | 0 | 0 |
|  | November 2012 | 4 | 4 | 0 | 0 |
|  | December 2012 | 0 **(6) (4 months)^¥^** | 0 **(6)** | 0 **(0)** | 0 **(0)** |
|  | January 2013 | 0 | 0 | 0 | 0 |
|  | February 2013 | 0 | 0 | 0 | 0 |
|  | March 2013 | 5 | 0 | 5 | 0 |
|  | April 2013 | 273 | 100 | 53 | 120 |
|  | May 2013 | 22 | 11 | 4 | 7 |
|  | June 2013 | 14 | 11 | 0 | 3 |
|  | July 2013 | 54 | 39 | 5 | 10 |
|  | August 2013 | 13 | 7 | 0 | 6 |
|  | September 2013 | 8 | 0 | 3 | 5 |
|  | October 2013 | 3 | 0 | 1 | 2 |
|  | November 2013 | 1 | 0 | 1 | 0 |
|  | December 2013 | 2 **(395) (12 months)** | 1 **(169)** | 1 **(73)** | 0 **(153)** |
|  | January 2014 | 0 | 0 | 0 | 0 |
|  | February 2014 | 0 | 0 | 0 | 0 |
|  | March 2014 | 3 | 2 | 1 | 0 |
|  | April 2014 | 7 | 2 | 3 | 2 |
|  | May 2014 | 17 | 10 | 3 | 4 |
|  | June 2014 | 40 | 27 | 0 | 13 |
|  | July 2014 | 4 | 2 | 0 | 2 |
|  | August 2014 | 22 | 16 | 1 | 5 |
|  | September 2014 | 3 | 0 | 2 | 1 |
|  | October 2014 | 1 | 0 | 1 | 0 |
|  | November 2014 | 0 | 0 | 0 | 0 |
|  | December 2014 | 0 **(97) (12 months)** | 0 **(59)** | 0 **(11)** | 0 **(27)** |
|  | January 2015 | 1 | 0 | 1 | 0 |
|  | February 2015 | 0 | 0 | 0 | 0 |
|  | March 2015 | 4 | 2 | 2 | 0 |
|  | April 2015 | 6 | 1 | 3 | 2 |
|  | May 2015 | 22 | 8 | 2 | 12 |
|  | June 2015 | 26 | 7 | 4 | 15 |
|  | July 2015 | 18 | 5 | 3 | 10 |
|  | August 2015 | 14 | 9 | 3 | 2 |
|  | September 2015 | 10 | 0 | 10 | 0 |
|  | October 2015 | 9 | 2 | 5 | 2 |
|  | November 2015 | 5 | 2 | 2 | 1 |
|  | December 2015 | 5 **(120) (12 months)** | 0 **(36)** | 5 **(40)** | 0 **(44)** |
|  | January 2016 | 1 | 0 | 1 | 0 |
|  | February 2016 | 2 | 0 | 2 | 0 |
|  | March 2016 | 3 | 0 | 3 | 0 |
|  | April 2016 | 15 | 3 | 4 | 8 |
|  | May 2016 | 28 | 10 | 3 | 15 |
|  | June 2016 | 30 | 12 | 2 | 16 |
|  | July 2016 | 35 | 13 | 3 | 19 |
|  | August 2016 | 14 | 5 | 7 | 2 |
|  | November 2017 | 6 **(134) (9 months)** | 4 **(47)** | 2 **(27)** | 0 **(60)** |
|  | (Still being continued, clump alive; August 2022) | - | - | - | - |
| **Total fruits (TF)** | | **752** | **317** | **151** | **284** |
| Clump 404 is still live (August 2022) with very low fruit production; its fruit output data till November 2017 is included.  Total fruits (TF) produced by accession 404: **37.19 Kg** (average weight of fruit 49.45 ± 25.75 g, n = 500). | | | | | |

| **[H] Clump 405** | | | | | |
| --- | --- | --- | --- | --- | --- |
| Sl. No | Month/year | Total fruits (TF) | Good fruits (GF) | Immature fruits (IF) | Predated fruits (PF) |
|  | February 2013 | 0 | 0 | 0 | 0 |
|  | March 2013 | 5 | 0 | 5 | 0 |
|  | April 2013 | 140 | 80 | 20 | 40 |
|  | May 2013 | 133 | 108 | 20 | 5 |
|  | June 2013 | 16 | 15 | 0 | 1 |
|  | July 2013 | 67 | 48 | 7 | 12 |
|  | August 2013 | 13 | 9 | 3 | 1 |
|  | September 2013 | 2 | 0 | 2 | 0 |
|  | October 2013 | 10 | 2 | 2 | 6 |
|  | November 2013 | 6 | 2 | 2 | 2 |
|  | December 2013 | 8 **(400) (11 months) ^¥^** | 6 **(270)** | 1 **(62)** | 1 **(68)** |
|  | January 2014 | 7 | 2 | 4 | 1 |
|  | February 2014 | 8 | 7 | 1 | 0 |
|  | March 2014 | 35 | 25 | 4 | 6 |
|  | April 2014 | 115 | 92 | 8 | 15 |
|  | May 2014 | 102 | 80 | 4 | 18 |
|  | June 2014 | 79 | 64 | 5 | 10 |
|  | July 2014 | 27 | 17 | 5 | 5 |
|  | August 2014 | 4 | 0 | 3 | 1 |
|  | September 2014 | 2 | 0 | 2 | 0 |
|  | October 2014 | 2 | 1 | 1 | 0 |
|  | November 2014 | 0 | 0 | 0 | 0 |
|  | December 2014 | 0 **(381) (12 months)** | 0 **(288)** | 0 **(37)** | 0 **(56)** |
|  | January 2015 | 12 | 10 | 2 | 0 |
|  | February 2015 | 4 | 3 | 1 | 0 |
|  | March 2015 | 4 | 3 | 1 | 0 |
|  | April 2015 | 20 | 3 | 4 | 13 |
|  | May 2015 | 24 | 4 | 5 | 15 |
|  | June 2015 | 24 | 9 | 5 | 10 |
|  | July 2015 | 23 | 16 | 3 | 4 |
|  | August 2015 | 12 | 6 | 4 | 2 |
|  | September 2015 | 6 | 2 | 2 | 2 |
|  | October 2015 | 2 | 0 | 2 | 0 |
|  | November 2015 | 1 **(132) (11 months)** | 0 **(56)** | 1 **(30)** | 0 **(46)** |
|  | December 2015  (Clump death) | 0 | 0 | 0 | 0 |
| **Total fruits (TF)** | | **913** | **614** | **129** | **170** |
| Total fruits (TF) produced by accession 405: **45.15 Kg** (average weight of fruit 49.45 ± 25.75 g, n = 500). | | | | | |
| **Table 3 [A-H]: ^¥^**(Total fruits (TF) in a month) & (number of months in a year of study) are given in brackets and bold. | | | | | |

**Table S4.** Flowering and fruiting durations of eight *M. baccifera* clumps.

| Clump No. | Flowering initiation | Flowering end | Flowering duration (months) | Fruiting initiation | Fruiting end | Fruiting duration (months) | Clump death | Time taken for death after stoppage of flowering  (months) |
| --- | --- | --- | --- | --- | --- | --- | --- | --- |
| 58 | April 2009 | August 2014 | 65 | May 2009 | July 2014 | 63 | January 2015 | 5 |
| 359 | February 2009 | April 2015 | 75 | March 2009 | April 2015 | 74 | May 2015 | 1 |
| 365 | January 2012 | January 2015 | 37 | February 2012 | December 2014 | 35 | February 2015 | 1 |
| 394 | February 2009 | October 2010 | 21 | March 2009 | October 2010 | 20 | November 2010 | 1 |
| 395 | March 2009 | October 2010 | 20 | April 2009 | October 2010 | 19 | October 2010 | 0 |
| 403 | March 2011 | June 2015 | 52 | April 2011 | May 2015 | 50 | August 2015 | 2 |
| 404 | September 2012 | Continuing* (August 2022) | 120 | October 2012 | Continuing/* (August 2022) | 119 | Continuing  - | Not dead |
| 405 | February 2013 | November 2015 | 34 | March 2013 | November 2015 | 33 | December 2015 | 1 |

**Table S5.** Summary of total fruits, good fruits, immature fruits, predated fruits in *M. baccifera*.

| Clump No. | Total fruits (TF)^ᴪ^ | Good fruits (GF) (%) | Immature fruits (IF) (%) | Predated fruits (PF) (%) |
| --- | --- | --- | --- | --- |
| 58 | 5752 | 3890 (67.63) | 864 (15.02) | 998 (17.35) |
| 359 | 9235 | 3640 (39.42) | 1985 (21.49) | 3610 (39.09) |
| 365 | 1832 | 1259 (68.72) | 254 (13.86) | 319 (17.41) |
| 394 | 9060 | 2940 (32.45) | 1781 (19.66) | 4339 (47.89) |
| 395 | 6440 | 1534 (23.82) | 1278 (19.84) | 3628 (56.34) |
| 403 | 4387 | 2613 (59.56) | 500 (11.40) | 1275 (29.06) |
| 404 | 752 | 317 (42.15) | 151 (20.08) | 284 (37.77) |
| 405 | 913 | 614 (67.25) | 129 (14.13) | 170 (18.62) |
| TF (%) | 38371 | 16807  (43.80%) | 6942  (18.09%) | 14623  (38.11%) |
| Total weight of fruits (8 clumps) 1897.45 Kg | | | | |
| Average weight of fruit = 49.45 ± 25.75 g, (average weight of 500 mature fruits representing eight clumps). | | | | |

^ᴪ‘^Total fruits’ (TF) in this study is the total of ‘Good fruits’ (GF), ‘Immature fruits’ (IF) and ‘Predated fruits’ (PF) in the total fruiting period of a clump or at specific monthly durations (as mentioned in the context).

**Table S6.** One month of highest fruit production, predation pattern in *M. baccifera*.

| Clump No. | Month of highest fruit production | Total fruits (TF)  (in the month of highest fruit production) | Good fruits (GF)  (in the month of highest fruit production) | Immature fruits (IF)  (in the month of highest fruit production) | Predated fruits (PF)  (in the month of highest fruit production) |
| --- | --- | --- | --- | --- | --- |
| 58 | May 2011 | 1129 | 872 | 140 | 117 |
| 359 | April 2010 | 1464 | 267 | 303 | 894 |
| 365 | April 2013 | 329 | 202 | 41 | 86 |
| 394 | April 2010 | 3601 | 414 | 716 | 2471 |
| 395 | April 2010 | 3230 | 280 | 605 | 2345 |
| 403 | June 2011 | 1488 | 827 | 151 | 510 |
| 404 | April 2013 | 273 | 100 | 53 | 120 |
| 405 | April 2013 | 140 | 80 | 20 | 40 |
| TF (%) | | 11654 | 3042  (26.10%) | 2029  (17.41%) | 6583  (56.49%) |

**Table S7.** Fruit dynamics, predation pattern in *M. baccifera*.

| Clump No. | Fruiting initiation | Fruiting end | Fruiting duration (months) | TF | Ave. fruit production per month | PF | Ave. fruit predation per month | % PF | Month of highest fruit production | TF  (in the month of highest fruit production) | PF  (in the month of highest fruit production) | PF% (in the month of highest fruit production) |
| --- | --- | --- | --- | --- | --- | --- | --- | --- | --- | --- | --- | --- |
| 58 | May 2009 | Jul 2014 | 63 | 5752 | 91.30 | 998 | 15.84 | 17.35 | May 2011 | 1129 | 117 | 10.36 |
| 359 | Mar 2009 | Apr 2015 | 74 | 9235 | 124.80 | 3610 | 48.78 | 39.09 | April 2010 | 1464 | 894 | 61.07 |
| 365 | Feb 2012 | Dec 2014 | 35 | 1832 | 52.34 | 319 | 9.11 | 17.41 | April 2013 | 329 | 86 | 26.14 |
| 394 | Mar 2009 | Oct 2010 | 20 | 9060 | 453.00 | 4339 | 216.95 | 47.81 | April 2010 | 3601 | 2471 | 68.62 |
| 395 | Apr 2009 | Oct 2010 | 19 | 6440 | 338.95 | 3628 | 190.95 | 56.34 | April 2010 | 3230 | 2345 | 72.60 |
| 403 | Apr 2011 | May 2015 | 50 | 4387 | 87.74 | 1275 | 25.50 | 29.06 | June 2011 | 1488 | 510 | 34.27 |
| 404 | Oct 2012 | Nov 2017 | 62 | 752 | 12.13 | 284 | 4.58 | 37.76 | April 2013 | 273 | 120 | 43.96 |
| 405 | Mar 2013 | Nov 2015 | 33 | 913 | 27.67 | 170 | 5.15 | 18.61 | April 2013 | 140 | 40 | 28.57 |

(Clump 404 is still live (August 2022) with very low fruit production; its fruit output data till November 2017 is included, Table S3).

**Table S8.** Duration of female, male stages and female-male interval in *M. baccifera*.

| **Floret No.** | . **Female Stage**  **(1-5)** | | | | | **Male Stage**  **(6-10)** | | | | | **Female- male interval**  **(6-1)**  **(Hours/Min)** |
| --- | --- | --- | --- | --- | --- | --- | --- | --- | --- | --- | --- |
|  | **1**  Stigma emergence  (Date & time) | **2**  Stigma full exposure  (Date & time) | **3**  Time for full stigma emergence (**2**-**1**)  (Hours/Min) | **4**  Stigma losing freshness  (Date & time) | **5**  Duration of stigma receptivity (**4**-**1**)  (Hours/Min) | **6**  Anthers emergence  (Date & time) | **7**  Anthers full exposure  (Date & time) | **8**  Anther dehiscence start  (Date & time) | **9**  Anther dehiscence end  Date & time) | **10**  Male stage duration  (**9**-**6**)  (Hours/Min) |  |
| 1 | 12/12/11  8.32 am | 12/12/11  10.50 am | **2/18** | 13/12/11  11.15 am | **26/43** | 14/12/11  12.15 pm | 14/12/11  2.24 pm | 14/12/11  2.30 pm | 14/12/11  4.15 pm | **4/00** | **51/43** |
| 2 | 12/12/11  8.37 am | 12/12/11  10.40 am | **2/03** | 13/12/11  11.47 am | **27/10** | 15/12/11  10.40 am | 15/12/11  12.24 pm | 15/12/11  2.05 pm | 15/12/11  3.45 pm | **5/05** | **74/03** |
| 3 | 12/12/11  8.46 am | 12/12/11  10.54 am | **2/08** | 13/12/11  2.40pm | **29/54** | 14/12/11  11.24 am | 14/12/11  1.24 pm | 14/12/11  2.28 pm | 14/12/11  4.24 pm | **5/00** | **50/38** |
| 4 | 12/12/11  9.15 am | 12/12/11  11.20 am | **2/05** | 13/12/11  11.24 am | **26/09** | 14/12/11  10.48 am | 14/12/11  12.28 pm | 14/12/11  2.54 pm | 14/12/11  4.43 pm | **5/55** | **49/33** |
| 5 | 14/12/11  7.53 am | 14/12/11  10.24 am | **2/31** | 15/12/11  10.24 am | **26/31** | 16/12/11  9.10 am | 16/12/11  11.58 am | 16/12/11  12.00 pm | 16/12/11  2.24 pm | **5/14** | **49/17** |
| 6 | 14/12/11  8.40 am | 14/12/11  10.57 am | **2/17** | 15/12/11  10.26 am | **25/46** | 16/12/11  9.12 am | 16/12/11  11.17 am | 16/12/11  11.20 pm | 16/12/11  1.48 pm | **4/36** | **48/32** |
| 7 | 14/12/11  9.07 am | 14/12/11  11.34 am | **2/27** | 15/12/11  9.12 am | **24/05** | 16/12/11  8.47 am | 16/12/11  11.10 am | 16/12/11  11.14 am | 16/12/11  2.20 pm | **5/33** | **47/40** |
| 8 | 14/12/11  9.10 am | 14/12/11  11.42 am | **2/32** | 15/12/11  9.34 am | **24/24** | 16/12/11  10.24 am | 16/12/11  12.54 pm | 16/12/11  1.00 pm | 16/12/11  3.28 pm | **5/04** | **49/14** |
| 9 | 14/12/11  9.16 am | 14/12/11  12.08 pm | **2/52** | 15/12/11  11.18 am | **26/02** | 16/12/11  9.02 am | 16/12/11  11.12 am | 16/12/11  11.15 am | 16/12/11  1.44 pm | **4/42** | **47/46** |
| 10 | 14/12/11  9.32 am | 14/12/11  11.24am | **1/52** | 15/12/11  9.25 am | **23/53** | 16/12/11  10.22 am | 16/12/11  12.54 pm | 16/12/11  12.57 pm | 16/12/11  2.58 pm | **4/36** | **48/50** |
| 11 | 14/12/11  9.40am | 14/12/11  11.54 am | **2/14** | 15/12/11  3.54 pm | **30/14** | 16/12/11  9.42 am | 16/12/11  12.10 pm | 16/12/11  12.15 pm | 16/12/11  2.40 pm | **4/58** | **48/02** |
| 12 | 14/12/11  10.07 am | 14/12/11  12.34 pm | **2/27** | 15/12/11  12.10 pm | **26/03** | 16/12/11  8.44 am | 16/12/11  10.24 am | 10/12/11  11.07 am | 16/12/11  1.58 pm | **5/14** | **46/37** |
| 13 | 19/12/11  8.50 am | 19/12/11  11.03 am | **2/13** | 20/12/11  8.35 am | **23/45** | 21/12/11  9.00 am | 21/12/11  11.47 am | 21/12/11  11.54 am | 21/12/11  2.24 pm | **5/24** | **48/10** |
| 14 | 19/12/11  9.02 am | 19/12/11  11.24 am | **2/22** | 22/12/11  8.40 am | **71/38** | 23/12/11  9.42 am | 23/12/11  11.44 am | 23/12/11  11.52 am | 23/12/11  2.57 pm | **5/15** | **96/40** |
| 15 | 19/12/11  9.04 am | 19/12/11  11.44 am | **2/40** | 20/12/11  11.54 am | **26/50** | 21/12/12  8.42 am | 21/12/11  11.25 am | 21/12/11  11.40 am | 21/12/11  2.20 pm | **5/38** | **47/38** |
| 16 | 19/12/11  9.11 am | 19/12/11  11.32 am | **2/21** | 21/12/11  12.10 pm | **50/59** | 23/12/11  9.46 am | 23/12/11  12.10 pm | 23/12/11  12.14 pm | 23/12/11  2.25 pm | **4/39** | **96/35** |
| 17 | 20/12/11  8.50 am | 20/12/11  11.22 am | **2/32** | 21/12/11  12.14 pm | **27/24** | 23/12/11  8.46 am | 23/12/11  11.04 am | 23/12/11  11.20 am | 23/12/11  2.28 pm | **5/42** | **71/56** |
| 18 | 21/12/11  8.42 am | 21/12/11  11.25 am | **2/43** | 22/12/11  9.20 am | **24/38** | 23/12/11  9.32 am | 23/12/11  11.48 am | 23/12/11  11.53 am | 23/12/11  1.40 pm | **4/08** | **48/50** |
| 19 | 21/12/11  9.02 am | 21/12/11  12.10 pm | **3/08** | 22/12/11  9.32 am | **24/30** | 23/12/11  9.14 am | 23/12/1111.10 am | 23/12/11  11.14 am | 23/12/11  1.48 pm | **4/34** | **48/12** |
| 20 | 21/12/11  9.08 am | 21/12/11  11.47 am | **2/39** | 22/12/11  9.07 am | **23/59** | 23/12/11  9.46 am | 23/12/11  12.24 pm | 23/12/11  12.42 pm | 23/12/11  2.48 pm | **5/02** | **48/38** |
| 21 | 21/12/11  9.28 am | 21/12/11  12.40 pm | **3/12** | 22/12/11  7.54 am | **22/26** | 23/12/11  8.28 am | 23/12/11  11.14 am | 23/12/11  11.20 am | 23/12/11  1.54 pm | **5/26** | **47/00** |
| 22 | 2/1/2012  7.40 am | 2/1/12  10.25 am | **2/45** | 3/1/12  7.45 am | **24/05** | 4/1/12  9.48 am | 4/1/12  11.08 am | 4/1/12  11.10 am | 4/1/12  12.48 pm | **3/0** | **50/08** |
| 23 | 2/1/2012  7.43 am | 2/1/12  10.57 am | **3/14** | 3/1/12  8.47 am | **25/04** | 5/1/12  10.20 am | 5/1/12  12.24 pm | 5/1/12  12.25 pm | 5/1/12  12.40 pm | **2/20** | **74/37** |
| 24 | 2/1/2012  8.46 am | 2/1/12  11.42 am | **2/56** | 3/1/12  11.48 am | **27/02** | 4/1/12  10.24 am | 4/1/12  12.48 pm | 4/1/12  12.50 pm | 4/1/12  1.47 pm | **3/23** | **49/38** |
| 25 | 2/1/2012  8.50 am | 2/1/12  11.32 am | **2/42** | 3/1/12  10.24 am | **25/34** | 5/1/12  9.17 am | 5/1/12  12.52 pm | 5/1/12  12.54 pm | 5/1/12  1.40 pm | **4/23** | **72/27** |
| 26 | 2/1/2012  8.50 am | 2/1/12  11.58 am | **3/08** | 3/1/12  11.28 am | **26/38** | 5/1/12  10.05 am | 5/1/12  12.28 pm | 5/1/12  12.30 pm | 5/1/12  2.20 pm | **4/15** | **73/15** |
| 27 | 2/1/2012  9.02 am | 2/1/12  12.10 am | **3/08** | 3/1/12  10.20 am | **25/18** | 5/1/12  9.31 am | 5/1/12  11.12 am | 5/1/12  11.14 am | 5/1/12  12.44 pm | **3/13** | **72/29** |
| 28 | 3/1/12  8.47 am | 3/1/12  10.54 am | **2/07** | 5/1/12  8.54 am | **48/07** | 6/1/12  10.02 am | 6/1/12  11.22 am | 6/1/12  11.24 am | 6/1/12  12.48 pm | **2/46** | **73/15** |
| 29 | 3/1/12  8.55 am | 3/1/12  11.08 am | **2/13** | 5/1/12  9.32 am | **48/37** | 6/1/12  9.15 am | 6/1/12  11.14 am | 6/1/12  11.15 am | 6/1/12  12.54 pm | **3/39** | **72/20** |
| 30 | 3/1/12  8.57 am | 3/1/12  11.07 am | **2/10** | 4/1/12  11.24 am | **26/27** | 5/1/12  8.33 am | 5/1/12  10.24 am | 5/1/12  10.24 am | 5/1/12  12.10 am | **3/37** | **47/36** |
| 31 | 3/1/12  9.08 am | 3/1/12  11.44 am | **2/36** | 4/1/12  10.24 am | **25/16** | 6/1/12  9.08 am | 6/1/12  11.18 am | 6/1/12  11.20 am | 6/1/12  1.44 pm | **4/36** | **72/00** |
| 32 | 3/1/12  10.05 am | 3/1/12  12.05 pm | **2/00** | 5/1/12  1.22 pm | **51/17** | 5/1/12  8.40 am | 5/1/12  10.08 am | 5/1/12  10.10 am | 5/1/12  12.24 pm | **3/44** | **46/35** |
| 33 | 4/1/12  9.08 am | 4/1/12  11.42 am | **2/34** | 5/1/12  8.48a m | **23/40** | 6/1/12  9.14 am | 6/1/12  11.20 am | 6/1/12  11.23 am | 6/1/12  12.48 pm | **3/34** | **48/06** |
| 34 | 4/1/12  9.10 am | 4/1/12  12.17 pm | **3/07** | 6/1/12  7.16 am | **46/06** | 6/1/12  8.41 am | 6/1/12  10.22 am | 6/1/12  10.25 am | 6/1/12  12.44 pm | **4/03** | **47/31** |
| 35 | 4/1/12  9.15 am | 4/1/12  11.28 am | **2/13** | 5/1/12  8.18 am | **23/03** | 5/1/12  9.16 am | 5/1/12  11.10 am | 5/1/12  11.15 am | 5/1/12  12.44 pm | **3/28** | **24/01** |
| 36 | 4/1/12  9.18 am | 4/1/12  11.48 am | **2/30** | 5/1/12  7.14 am | **21/56** | 5/1/12  8.38 am | 5/1/12  10.16 am | 5/1/12  10.18 am | 5/1/12  12.17 pm | **3/39** | **23/20** |
| 37 | 4/1/12  9.24 am | 4/1/12  12.24 pm | **3/00** | 5/1/12  8.21 am | **22/57** | 5/1/12  10.12 am | 5/1/12  11.20 am | 5/1/12  11.22 am | 5/1/12  12.14 pm | **2/02** | **24/48** |
| **Average** |  |  | **151.89 ± 22.99 min** |  | **1790.54 ± 657.75 min** |  |  |  |  | **261.81 ± 58.58 min** | **3304.32 ± 1012.02 min** |

**Table S9.** Slug/snail predation in clump 58.

| **Clump 58**  Month/year | 1-7 days | | 8-14 days | | 15-21 days | | 22-28 days | | 29-42 days | |
| --- | --- | --- | --- | --- | --- | --- | --- | --- | --- | --- |
|  | Fruits observed  (FO) | Fruits  attacked  (FA) | FO | FA | FO | FA | FO | FA | FO | FA |
| August 2010 | 82 | 5 | 109 | 15 | 117 | 20 | 90 | 1 | 77 | 1 |
| September 2010 | 65 | 3 | 60 | 7 | 63 | 8 | 52 | 0 | 60 | 0 |
| October 2010 | 38 | 1 | 55 | 5 | 50 | 5 | 60 | 0 | 42 | 0 |
| November 2010 | 43 | 0 | 40 | 1 | 47 | 0 | 44 | 0 | 40 | 0 |
| December 2010 | 53 | 0 | 47 | 0 | 40 | 0 | 42 | 0 | 38 | 0 |
| January 2011 | 25 | 0 | 25 | 0 | 20 | 0 | 25 | 0 | 20 | 0 |
| February 2011 | 10 | 0 | 10 | 0 | 10 | 0 | 10 | 0 | 10 | 0 |
| March 2011 | 20 | 0 | 15 | 0 | 10 | 0 | 10 | 0 | 10 | 0 |
| April 2011 | 10 | 0 | 10 | 0 | 15 | 0 | 8 | 0 | 10 | 0 |
| May 2011 | 10 | 0 | 10 | 0 | 15 | 1 | 10 | 0 | 10 | 0 |
| June 2011 | 63 | 6 | 91 | 15 | 65 | 12 | 55 | 1 | 52 | 0 |
| July 2011 | 56 | 7 | 75 | 14 | 74 | 15 | 40 | 1 | 35 | 1 |
| August 2011 | 37 | 3 | 40 | 6 | 45 | 7 | 40 | 0 | 30 | 0 |
| Total fruits under different age group | 512 | 25 | 587 | 63 | 571 | 68 | 486 | 3 | 434 | 2 |
| Predation (%) | - | (4.88%) | - | (10.73%) | - | (11.91%) | - | (0.62%) | - | (0.46%) |
| Total fruits (of all age groups)  Total fruits (of all age groups) attacked  Total predation (%) | | | | | | | | | | 2590  161  (6.22%) |

**Table S10.** Fruit predation breakup in clump 359 of *M. baccifera*.

| **Clump 359**  Month/year | Total fruits (TF) | Good fruits (GF) | Immature fruits (IF) | Predated fruits (PF) | Monkeys | Boars | Rodents**^¥^** | Slugs /snails | Herbivores |
| --- | --- | --- | --- | --- | --- | --- | --- | --- | --- |
| May 2009 | 200 | 187 | 13 | 0 | - | - | - | - | - |
| June 2009 | 301 | 198 | 27 | 76 | 40 | 26 | - | 8 | 2 |
| July 2009 | 414 | 270 | 82 | 62 | 34 | 14 | 2 | 10 | 2 |
| August 2009 | 396 | 102 | 59 | 235 | 160 | 63 | 6 | 5 | 1 |
| September 2009 | 252 | 34 | 77 | 141 | 92 | 38 | 8 | 2 | 1 |
| October 2009 | 11 | 5 | 5 | 1 | - | 1 | - | - | - |
| November 2009 | 34 | 13 | 13 | 8 | 3 | 3 | 2 | - | - |
| December 2009 | 81 | 13 | 38 | 30 | 15 | 7 | 8 | - | - |
| January 2010 | 27 | 1 | 16 | 10 | 4 | 2 | 4 | - | - |
| February 2010 | 12 | 0 | 12 | 0 | - | - | - | - | - |
| March 2010 | 38 | 20 | 6 | 12 | 8 | 4 | - | - | - |
| April 2010 | 1464 | 267 | 303 | 894 | 745 | 135 | 10 | 4 | - |
| May 2010 | 778 | 290 | 234 | 254 | 162 | 68 | 14 | 10 | - |
| June 2010 | 698 | 202 | 233 | 263 | 154 | 80 | 10 | 18 | 1 |
| July 2010 | 274 | 61 | 88 | 125 | 58 | 37 | 8 | 20 | 2 |
| August 2010 | 70 | 23 | 28 | 19 | 7 | 5 | 2 | 3 | 2 |
| September 2010 | 3 | 0 | 2 | 1 | - | 1 | - | - | - |
| October 2010 | 11 | 3 | 3 | 5 | 2 | 2 | 1 | - | - |
| November 2010 | 2 | 0 | 2 | 0 | - | - | - | - | - |
| December 2010 | 4 | 0 | 4 | 0 | - | - | - | - | - |
| January 2011 | 3 | 0 | 3 | 0 | - | - | - | - | - |
| February 2011 | 3 | 0 | 3 | 0 | - | - | - | - | - |
| March 2011 | 2 | 0 | 2 | 0 | - | - | - | - | - |
| April 2011 | 7 | 6 | 1 | 0 | - | - | - | - | - |
| May 2011 | 819 | 236 | 154 | 429 | 366 | 48 | 10 | 5 | - |
| June 2011 | 1102 | 383 | 209 | 510 | 376 | 108 | 12 | 12 | 2 |
| July 2011 | 468 | 214 | 82 | 172 | 91 | 45 | 10 | 22 | 4 |
| August 2011 | 382 | 211 | 73 | 98 | 50 | 30 | 6 | 10 | 2 |
| September 2011 | 247 | 83 | 52 | 112 | 55 | 45 | 5 | 5 | 2 |
| October 2011 | 97 | 60 | 23 | 14 | 7 | 5 | 2 | - | - |
| November 2011 | 10 | 3 | 1 | 6 | 3 | 2 | 1 | - | - |
| December 2011 | 12 | 4 | 4 | 4 | 1 | 2 | 1 | - | - |
| **Total fruits**  **(%)** | **8222** | **2889 (35.14%)** | **1852 (22.52%)** | **3481 (42.34%)** | **2433 (69.89%)** | **771 (22.15%)** | **122 (3.50%)** | **134 (3.85%)** | **21**  **(0.60%)** |

**^¥^** High rates of predation by rats were observed in stored fruits.

**Table S11.** Borer larvae attack in clump 58 of *M. baccifera*.

| **Clump 58**  Month/Year | Fruits observed | Fruits attacked | | | | | | | |
| --- | --- | --- | --- | --- | --- | --- | --- | --- | --- |
|  |  | 1-7 days | 8-14 days | 15-21 days | 22-28 days | 29-35 days | 36-42 days | Total hits | % hits |
| January 2011 | - | 0 | 0 | 0 | 0 | 0 | 0 | 0 | 0 |
| February 2011 | - | 0 | 0 | 0 | 0 | 0 | 0 | 0 | 0 |
| March 2011 | - | 0 | 0 | 0 | 0 | 0 | 0 | 0 | 0 |
| April 2011 | 878 | 0 | 2 | 23 | 30 | 22 | 49 | 126 | 14.35% |
| May 2011 | 1731 | 0 | 9 | 38 | 18 | 23 | 29 | 117 | 6.76% |
| June 2011 | 560 | 0 | 0 | 8 | 12 | 10 | 5 | 35 | 6.25% |
| July 2011 | 170 | 0 | 0 | 1 | 2 | 2 | 1 | 6 | 3.53% |
| August 2011 | 42 | 0 | 0 | 0 | 1 | 0 | 0 | 1 | 2.38% |
| September 2011 | 193 | 0 | 0 | 0 | 0 | 0 | 0 | 0 | 0 |
| October 2011 | 24 | 0 | 0 | 0 | 0 | 0 | 0 | 0 | 0 |
| November 2011 | 25 | 0 | 0 | 0 | 0 | 0 | 0 | 0 | 0 |
| December 2011 | - | 0 | 0 | 0 | 0 | 0 | 0 | 0 | 0 |
| **Total fruits**  **(% hits against total fruits)** | **3623** | **0** | **11 (0.30%)** | **70 (1.93%)** | **63 (1.74%)** | **57 (1.57%)** | **84**  **(2.32%)** | **285** | **7.87%** |

**Fig S1 to S18.** *M. baccifera* fruits, floral visitors, fruit predators.


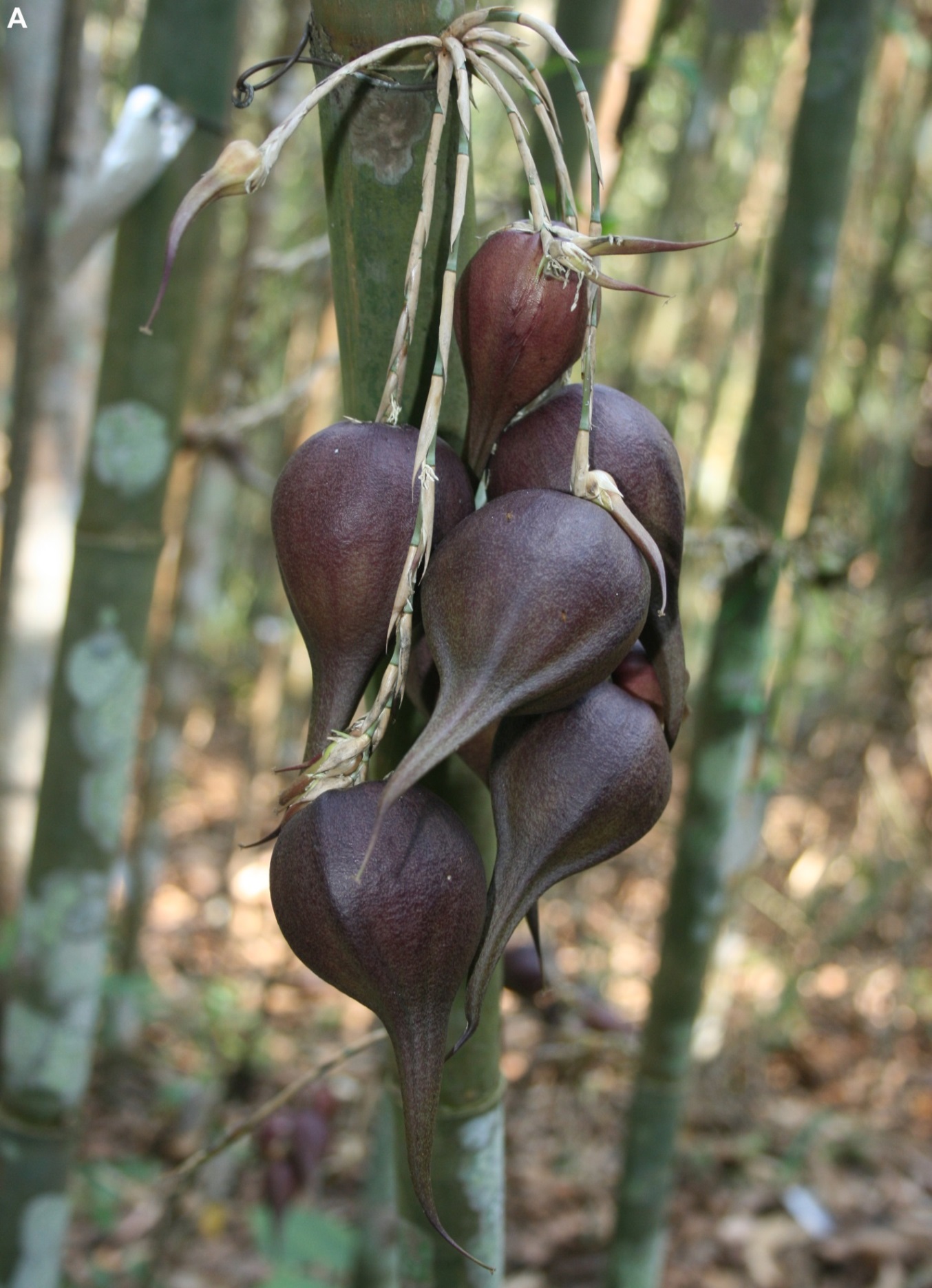


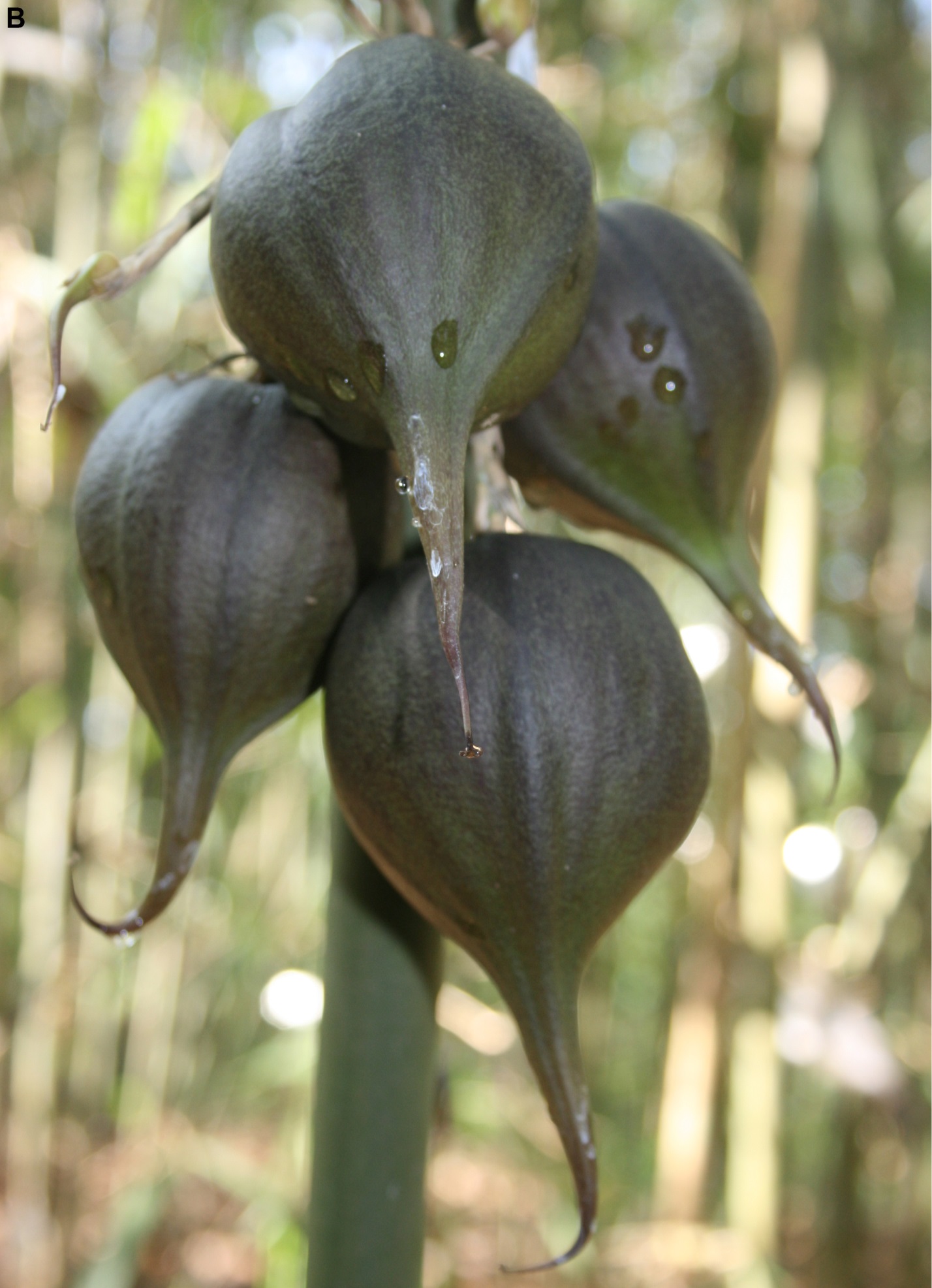

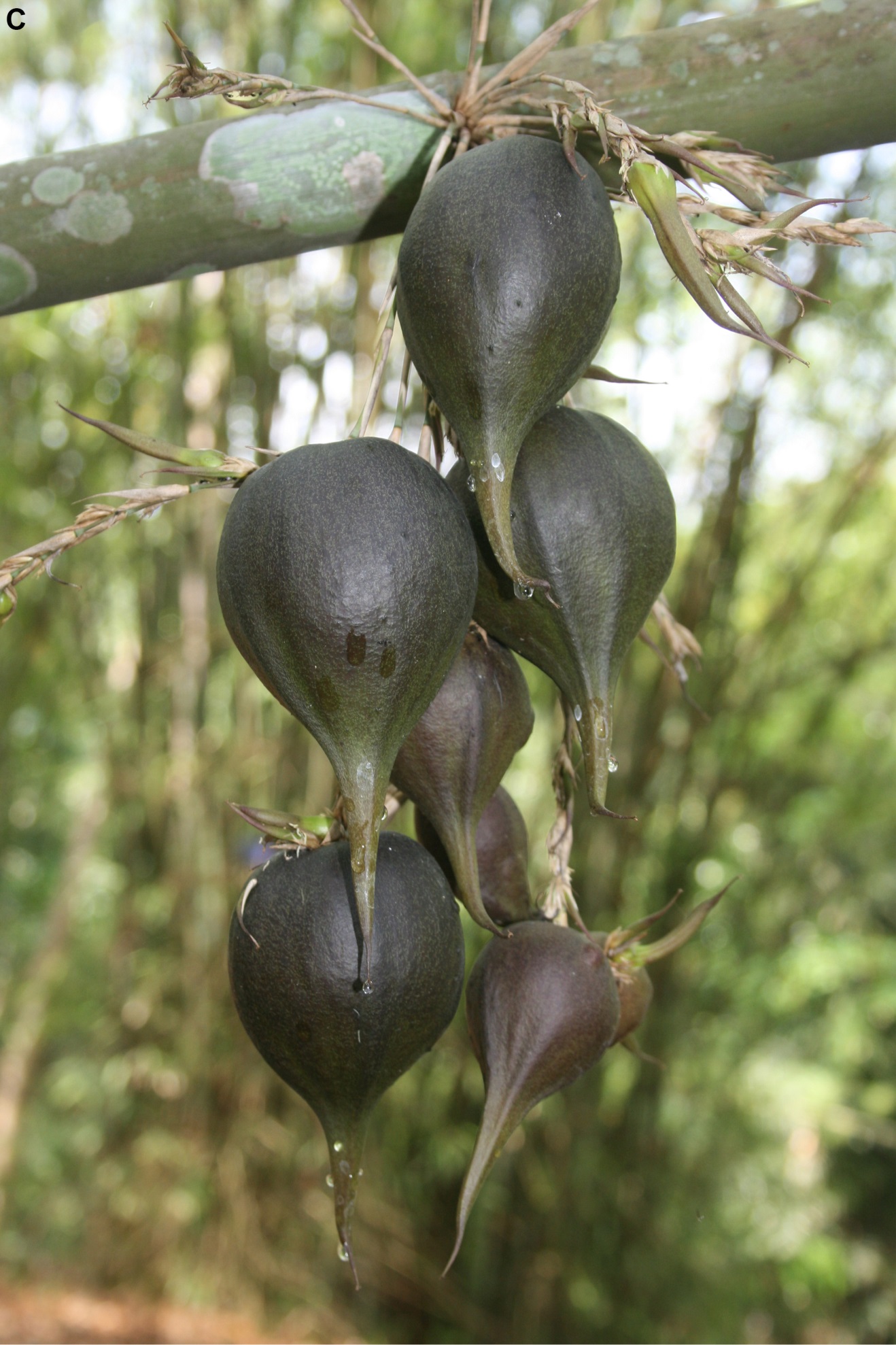

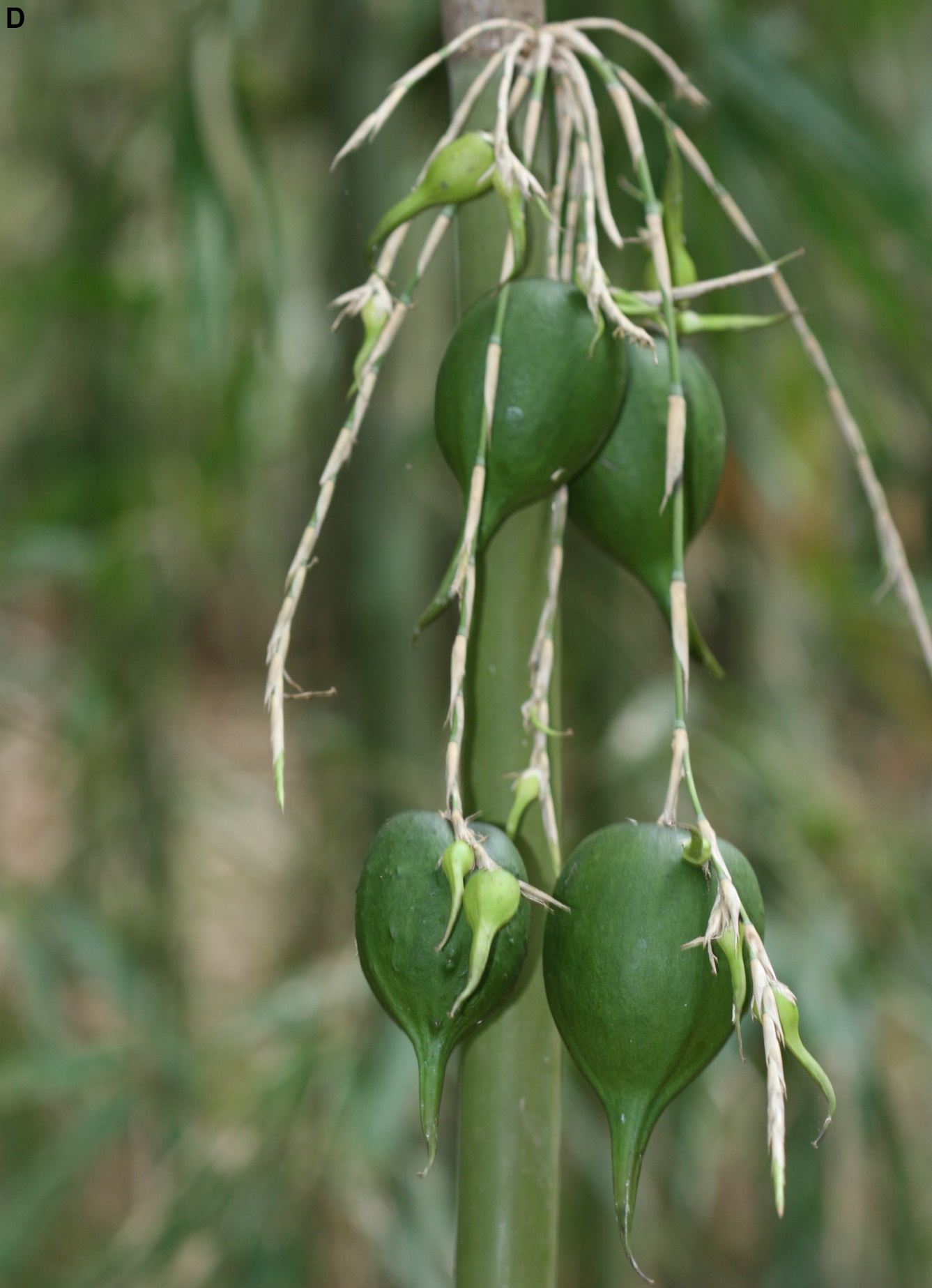


**Fig S1. (A-D).** *M. baccifera* fruits at JNTBGRI Bambusetum.


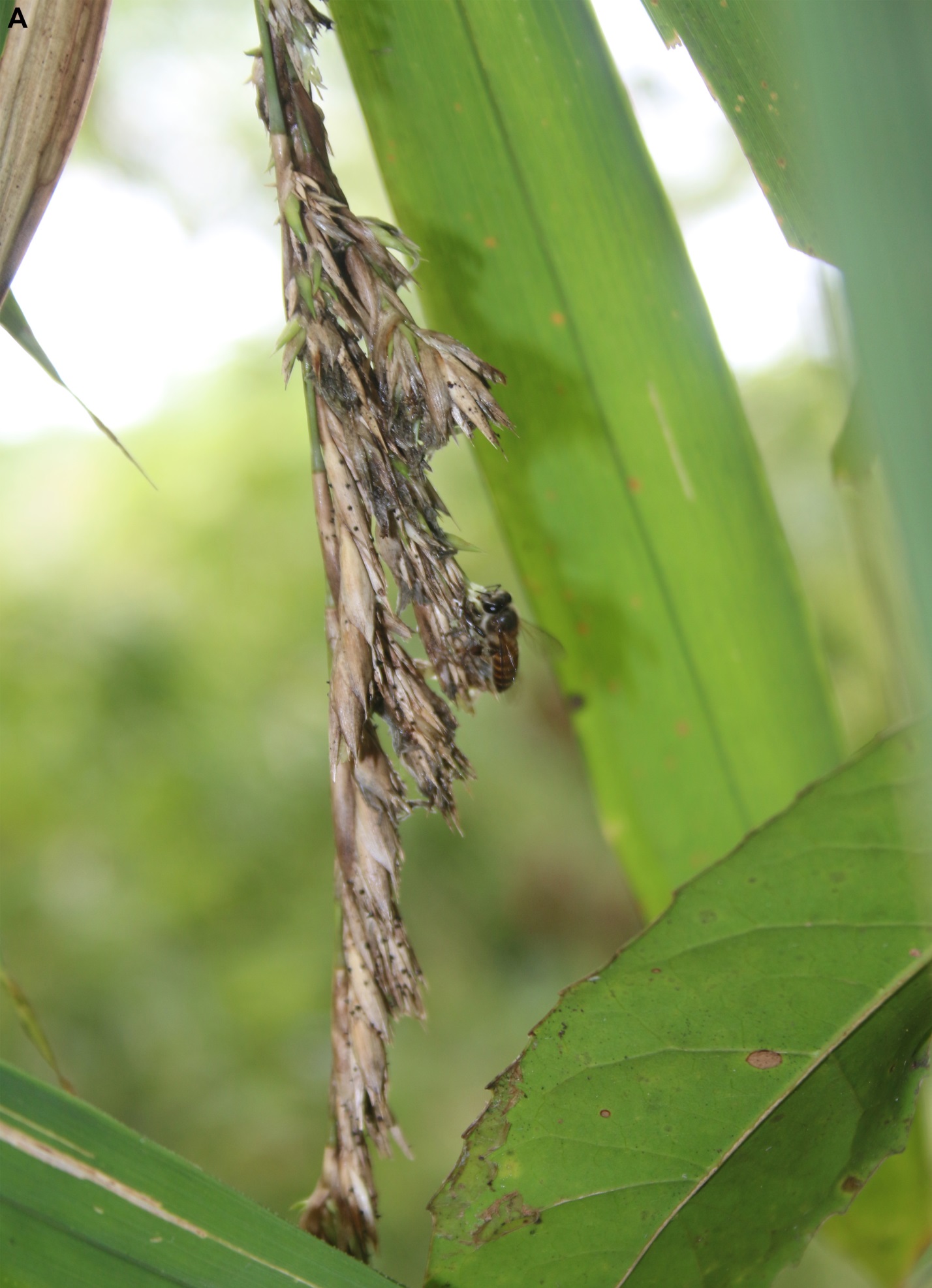

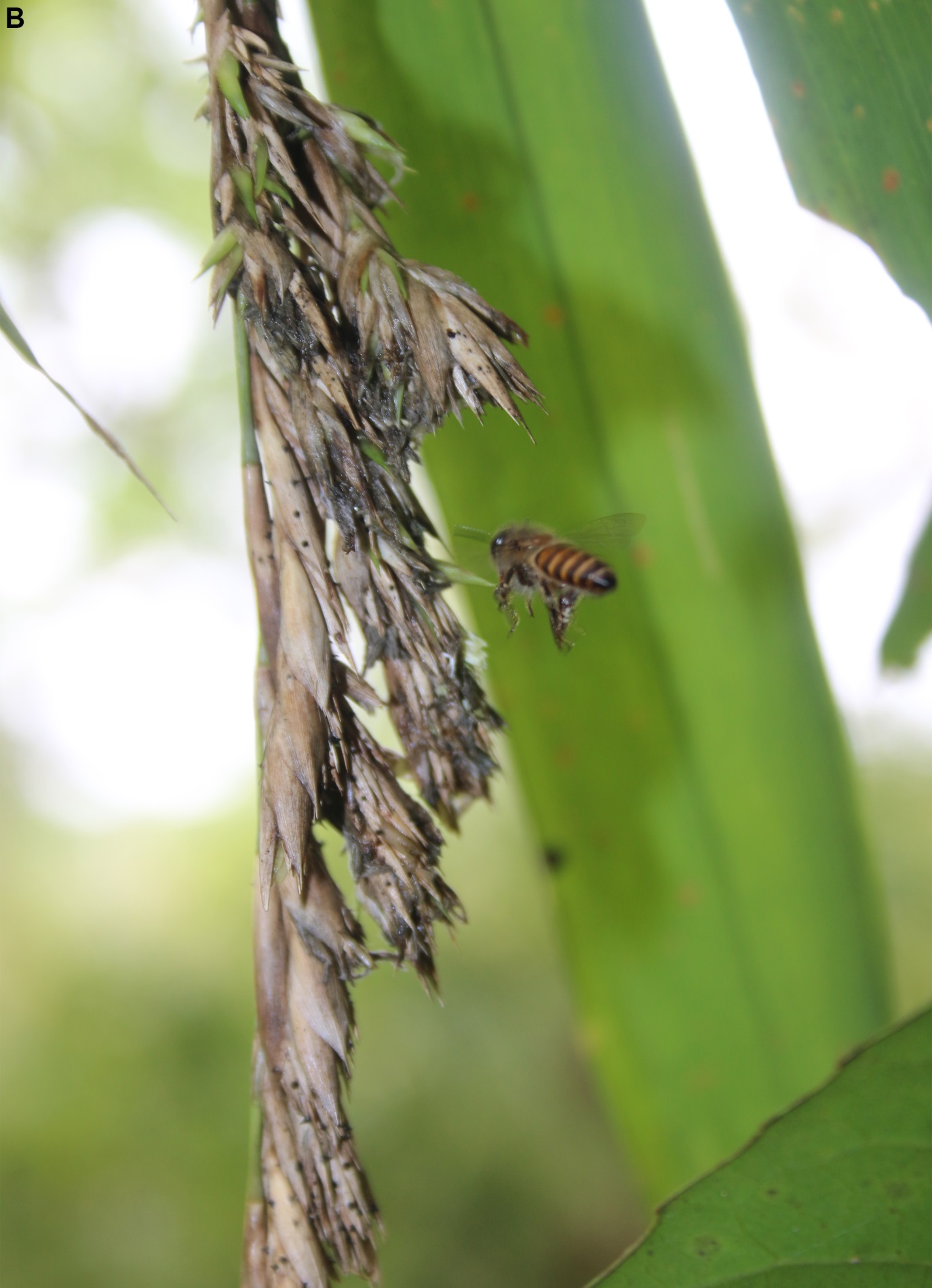


**Fig S2. (A-B).** *A. cerana indica* on *M. baccifera* inflorescence.


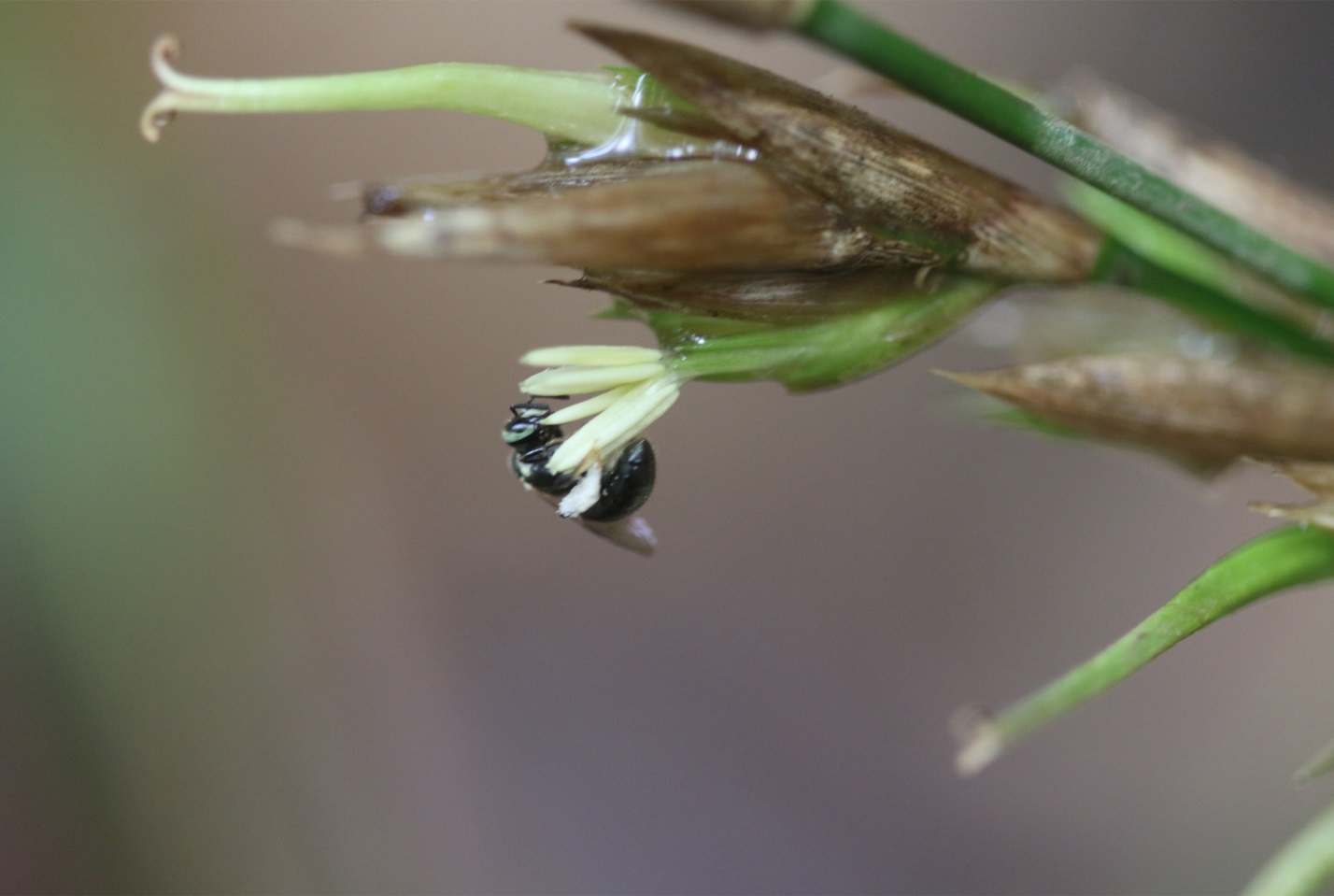


**Fig S3.** *M. baccifera* male flower with bee *H. taprabonae*.


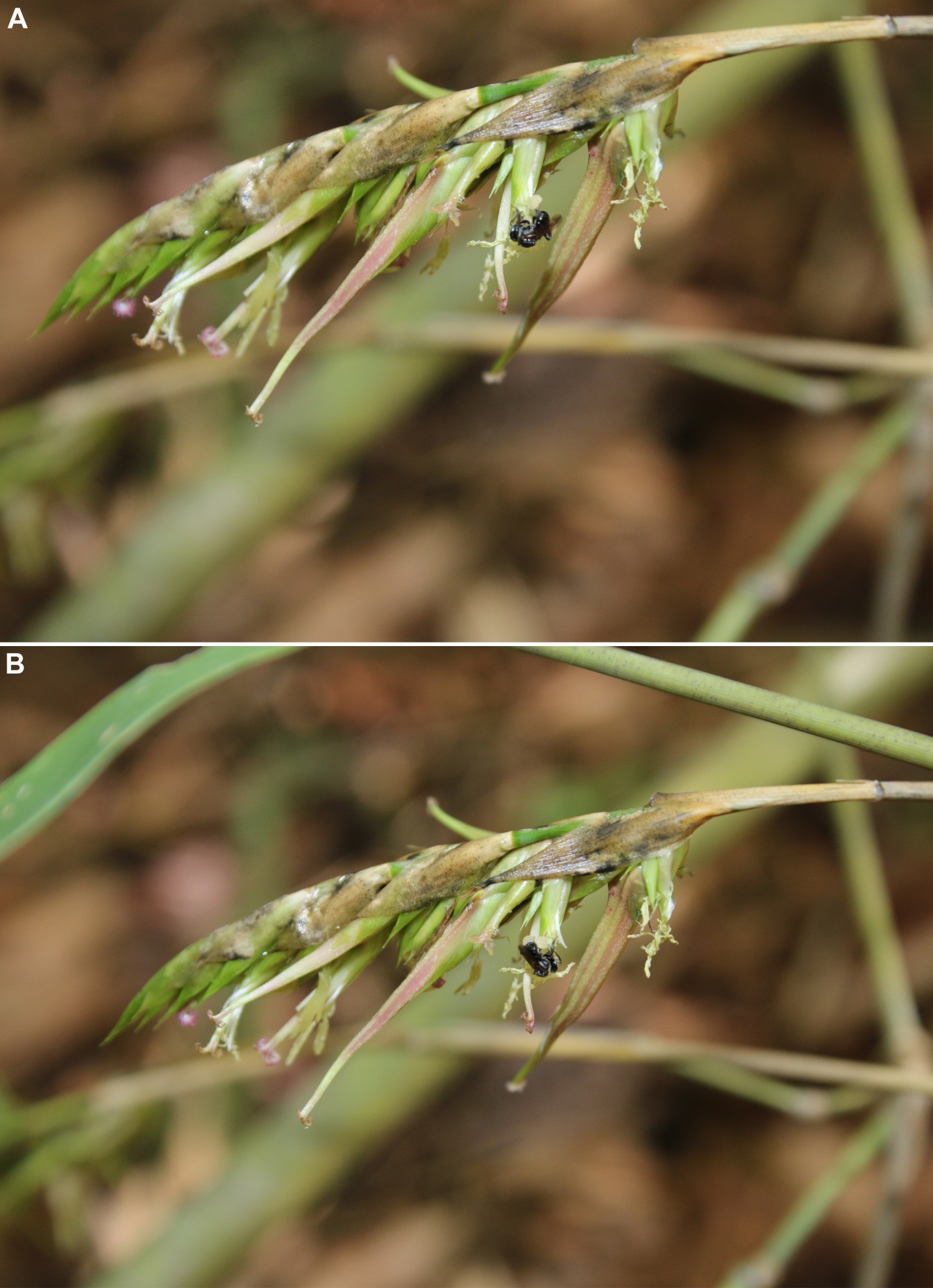


**Fig S4. (A-B).** *M. baccifera*, bee activity, *T. iridipennis*.


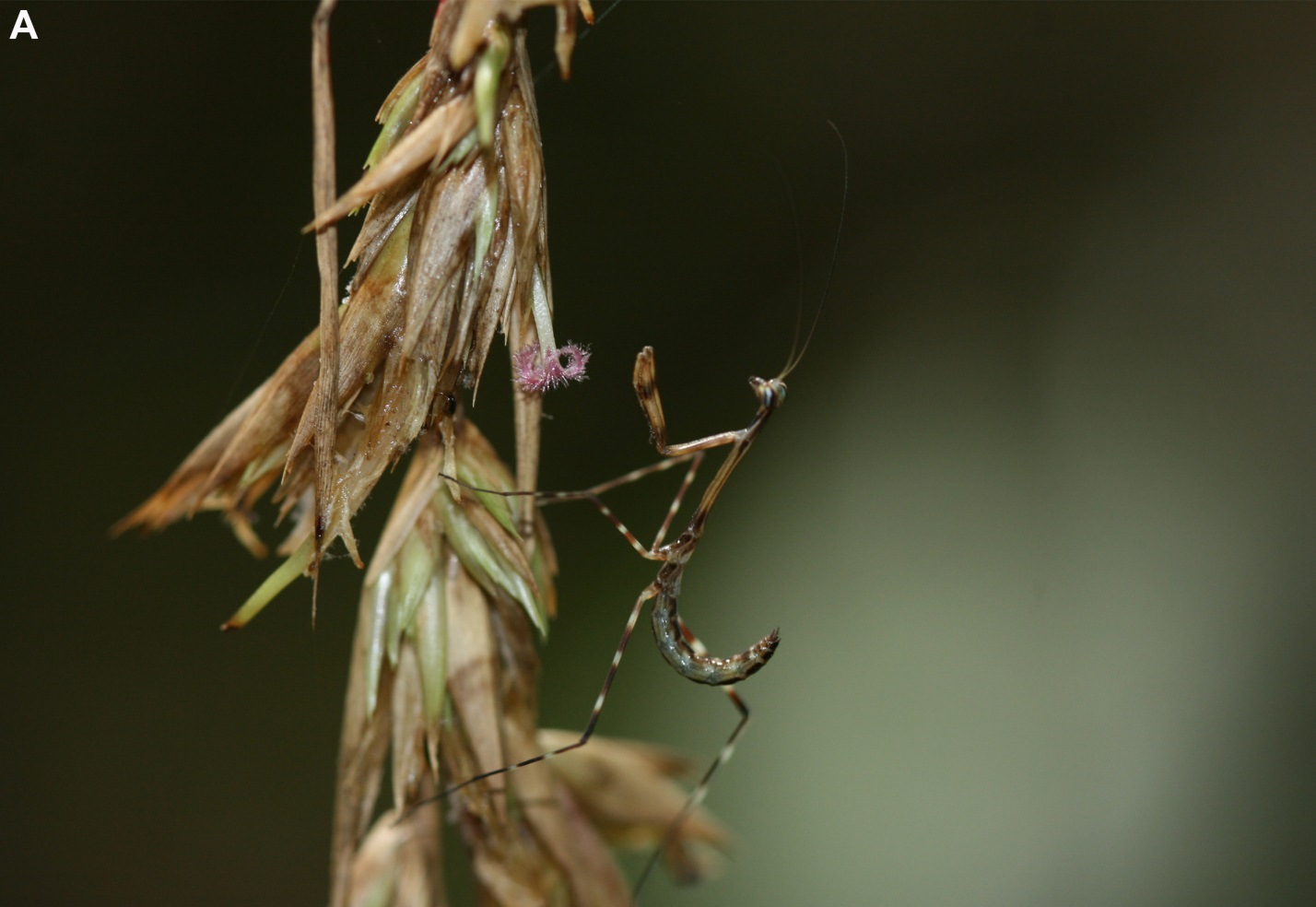

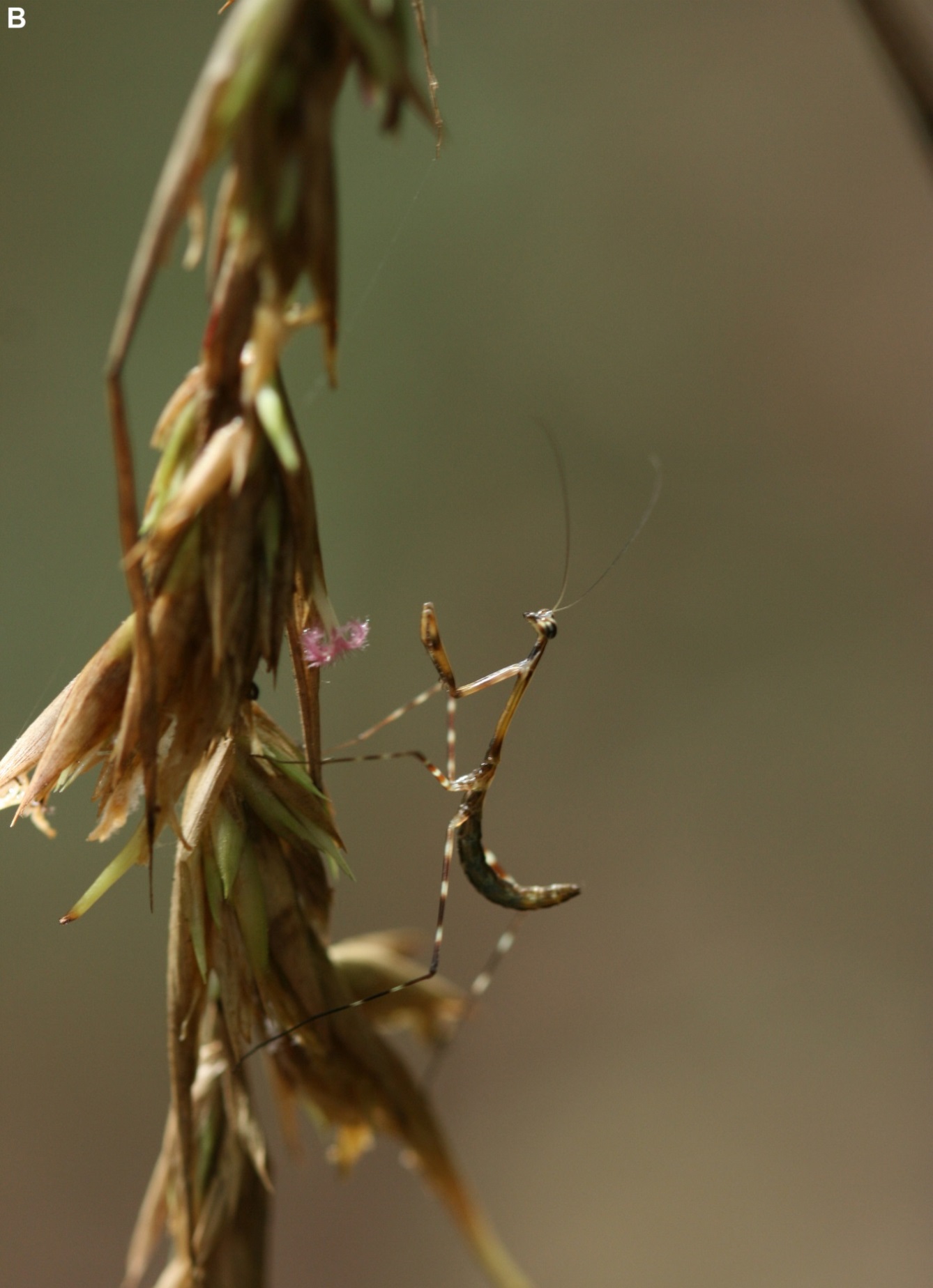


**Fig S5. (A-B).** Mantis, *E. indica* on *M. baccifera* inflorescence.

**
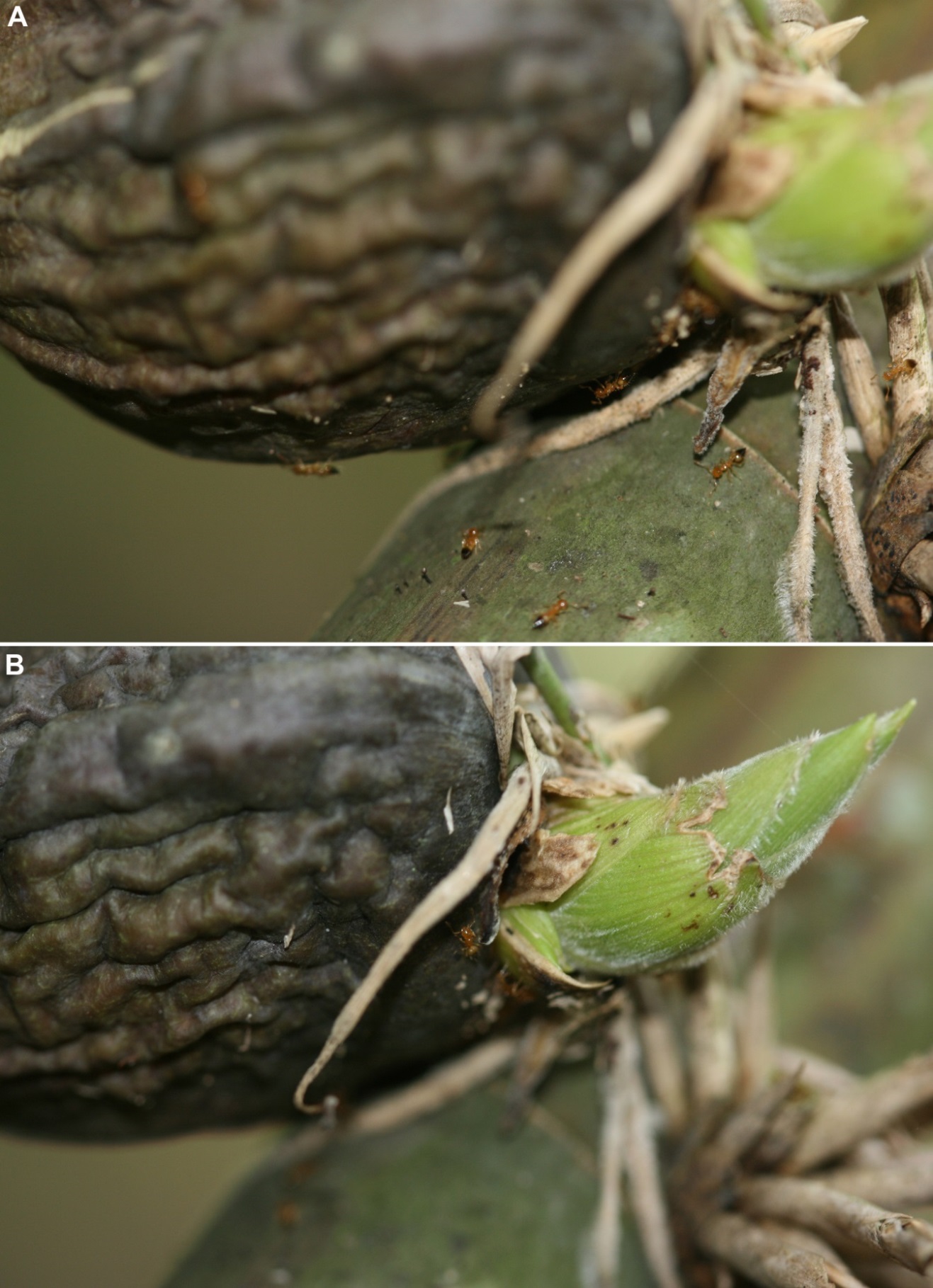
**

**Fig S6. (A-B).** Ants, *C. biroi* on fruits and internodes.


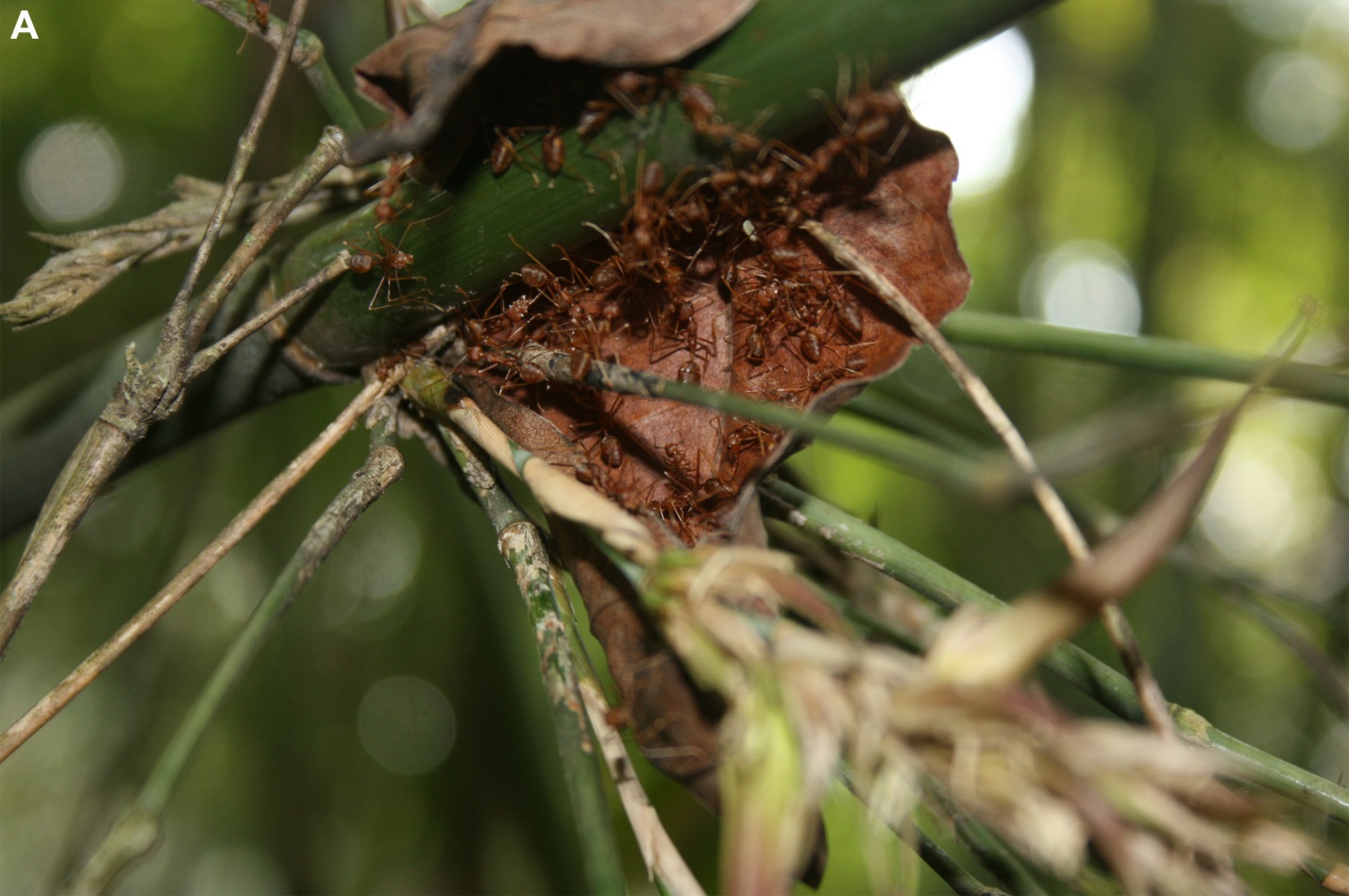

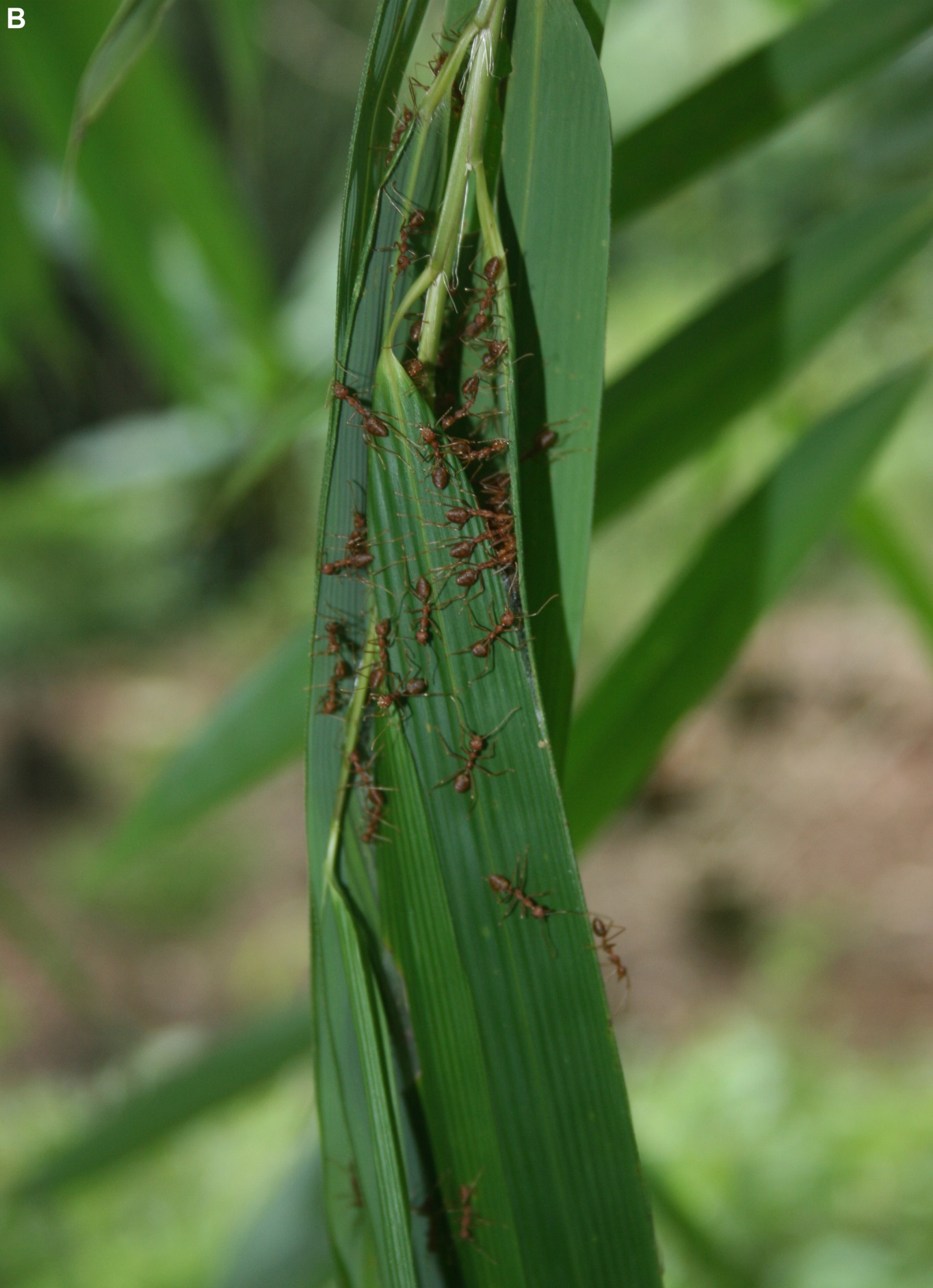


**Fig S7. (A-B).** Ants, *O. smaragdina* on *M. baccifera*.


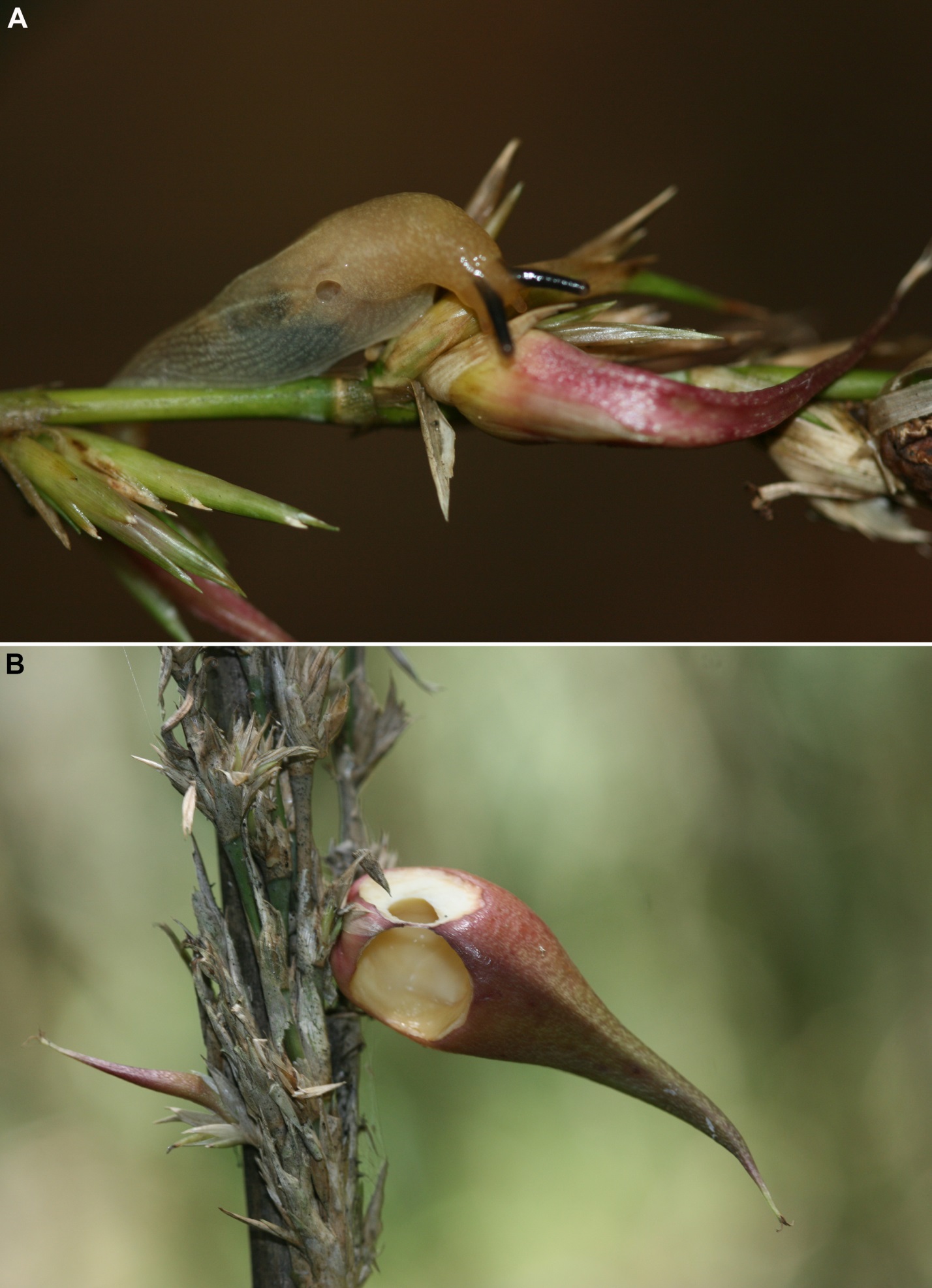

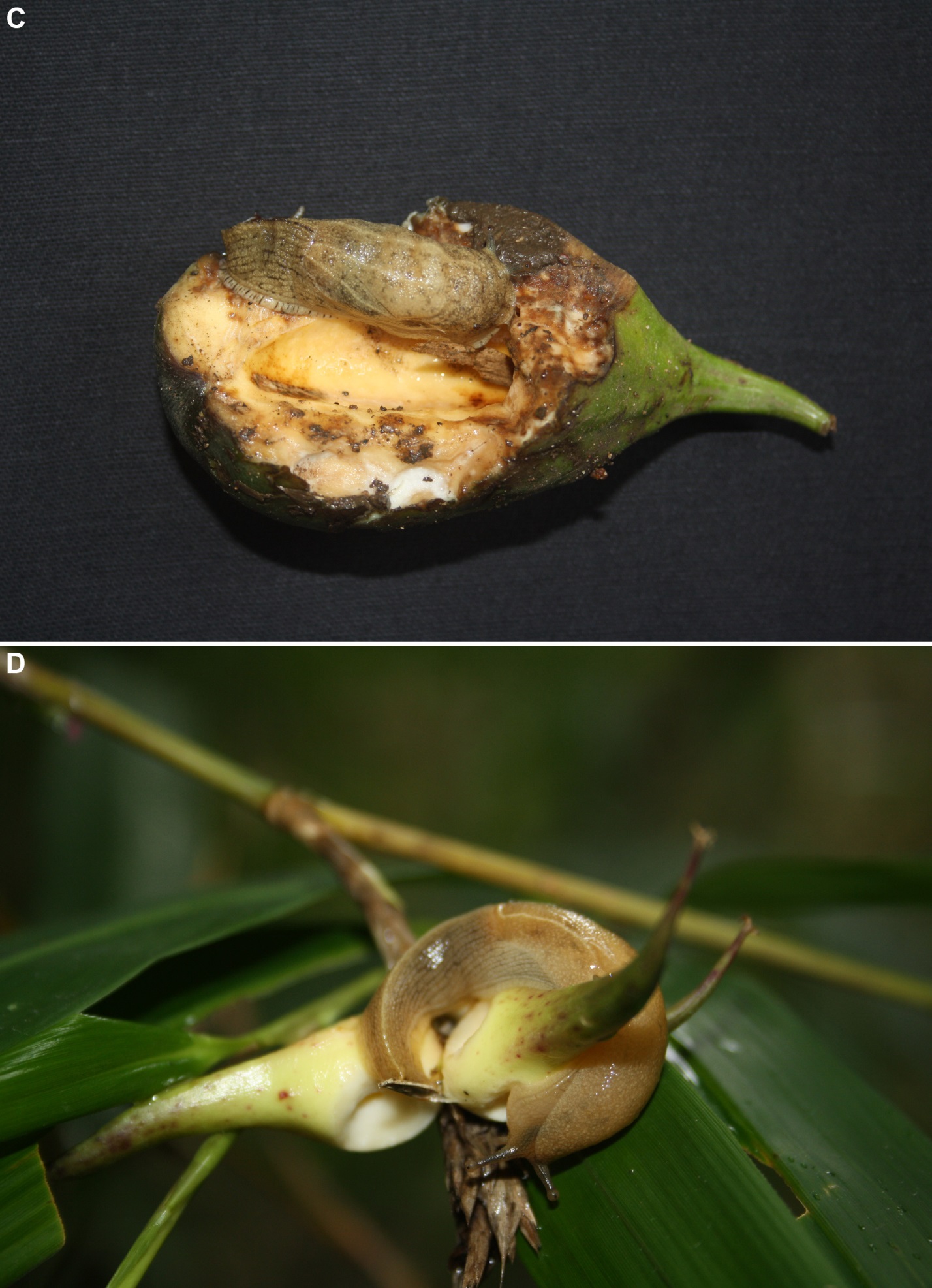


**
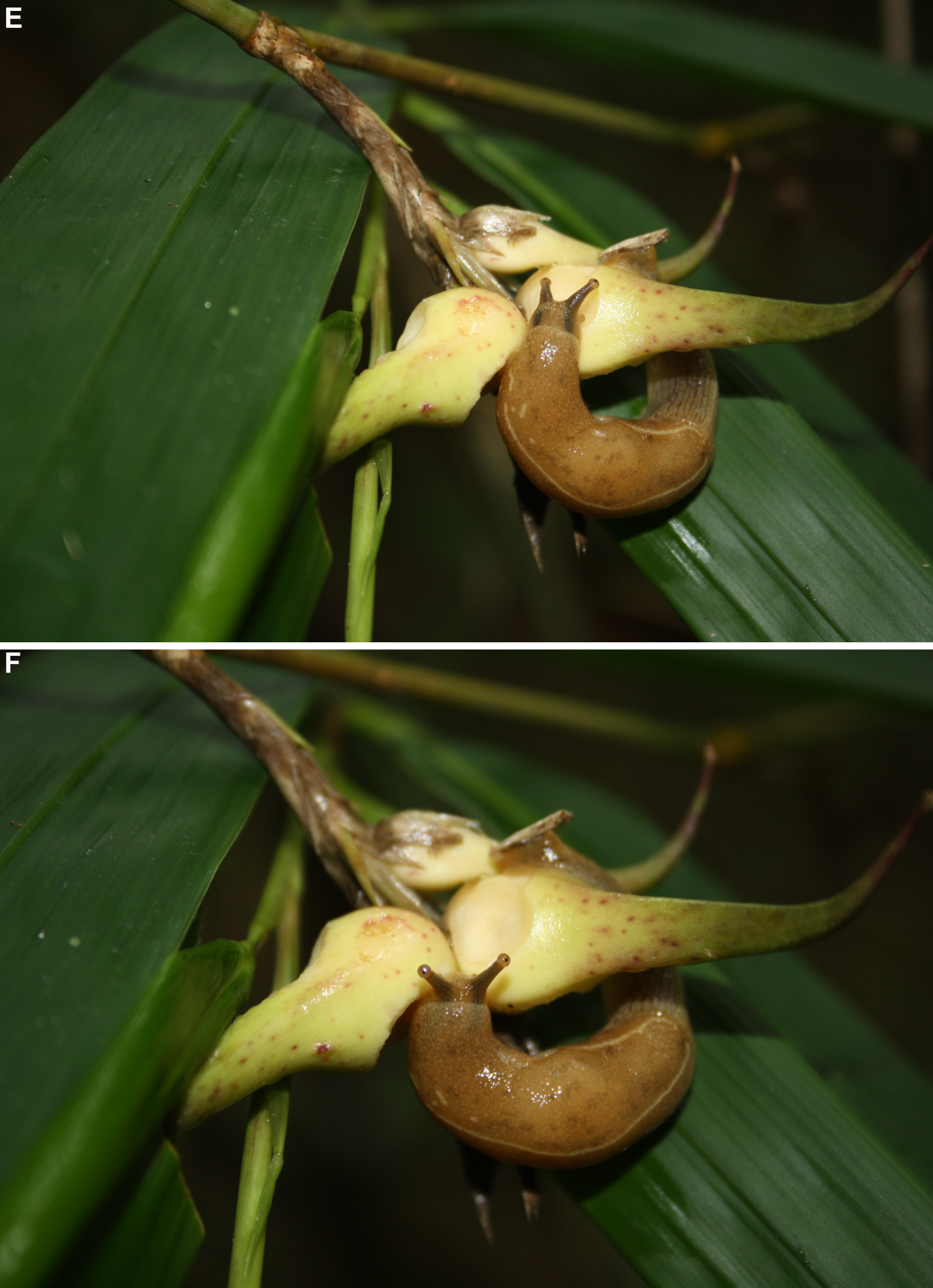
**

**Fig S8. (A-F).** *M. baccifera*, slug attack, *M. dussumieri* on young fruits.


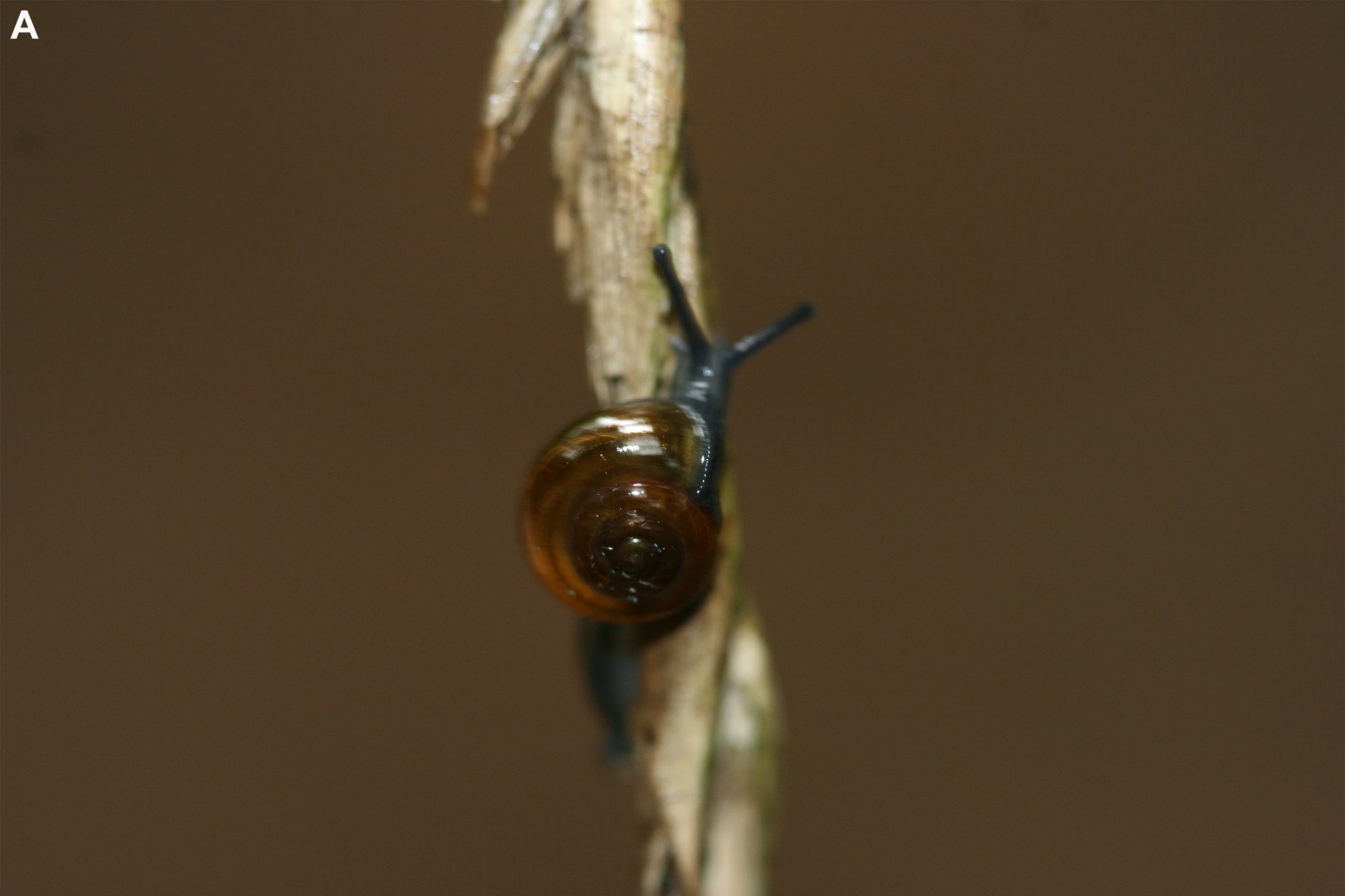

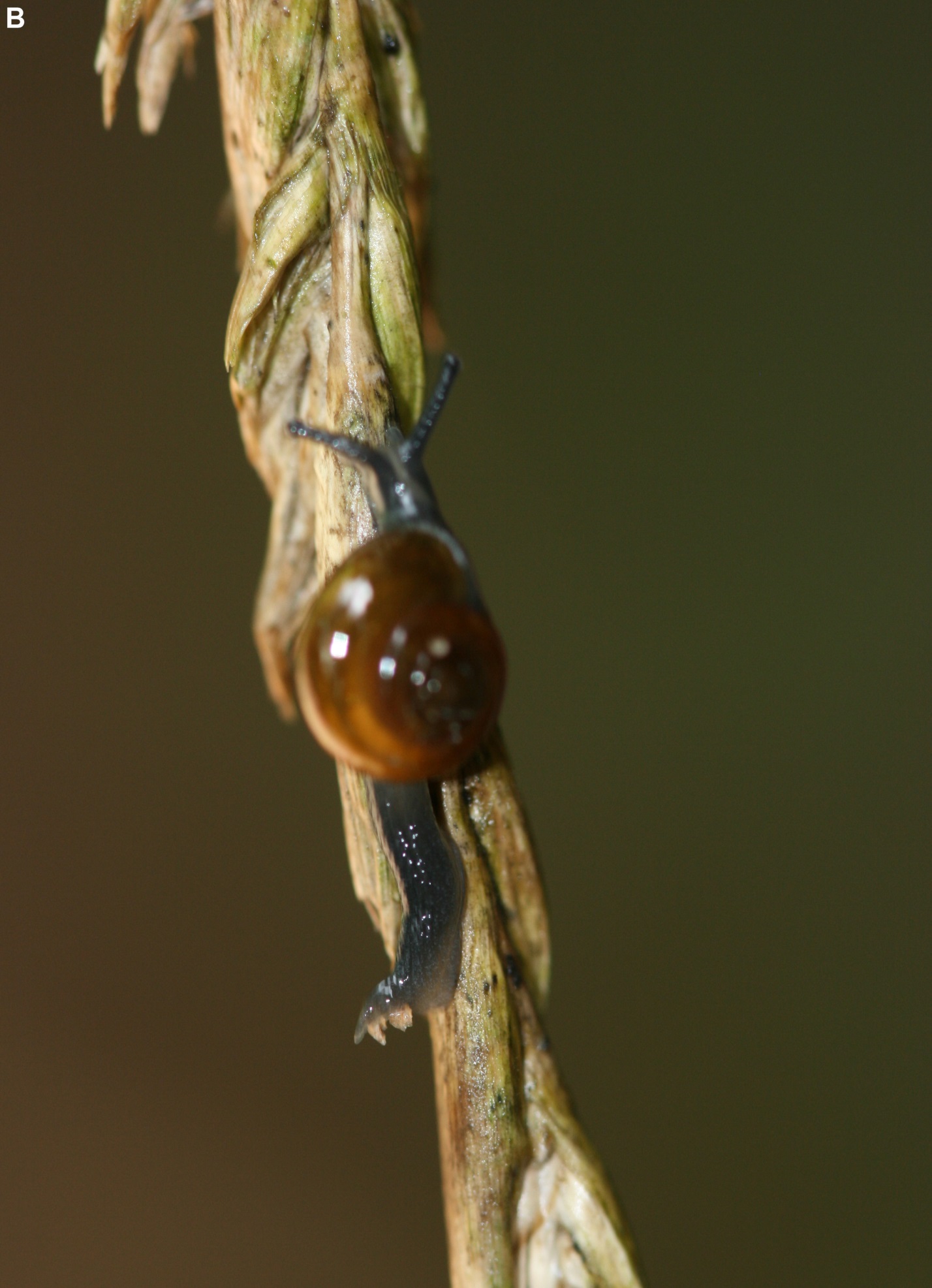

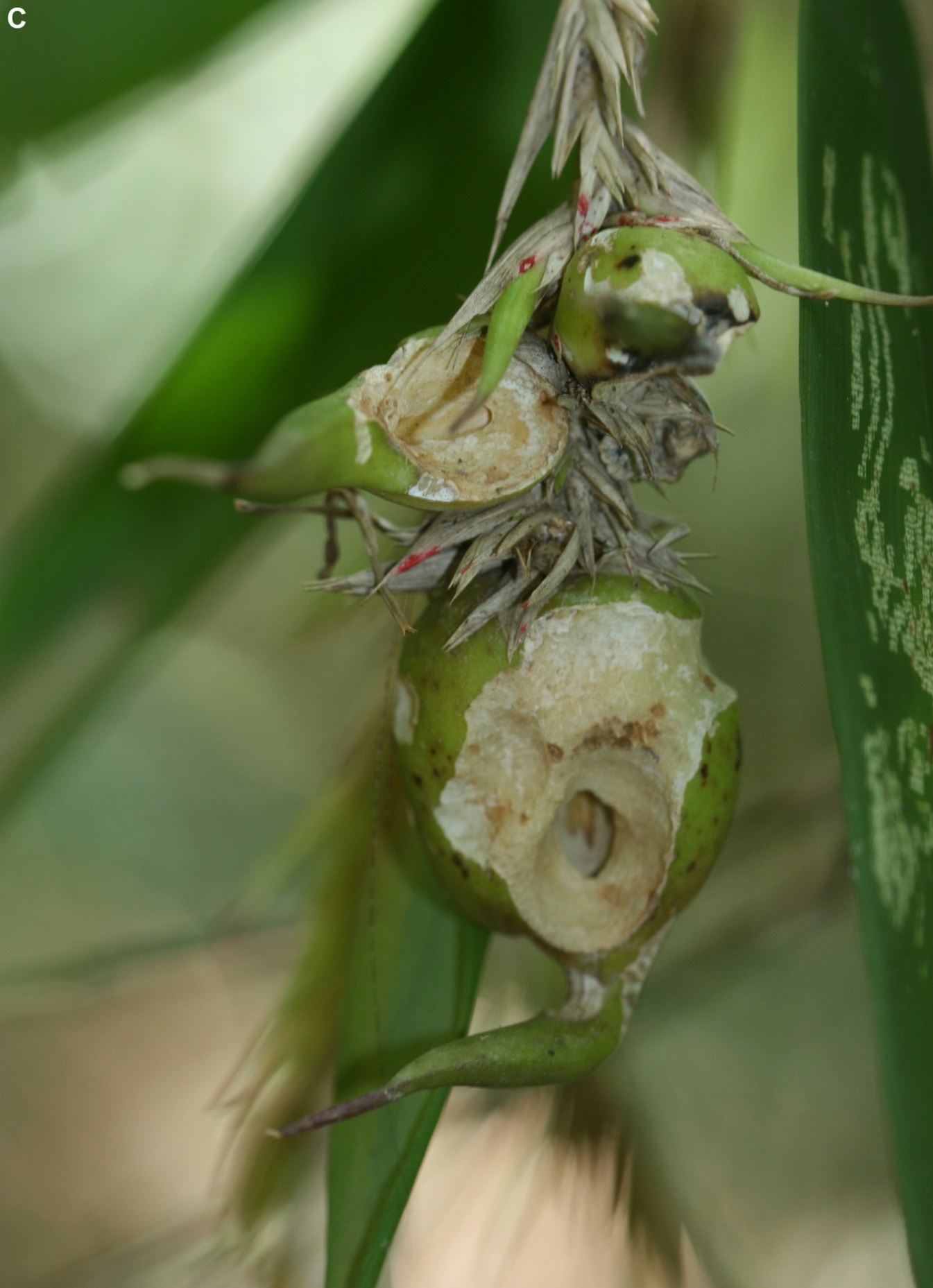


**Fig S9. (A-C).** *M. baccifera*, snail *C. bistrialis* attacking young fruits.


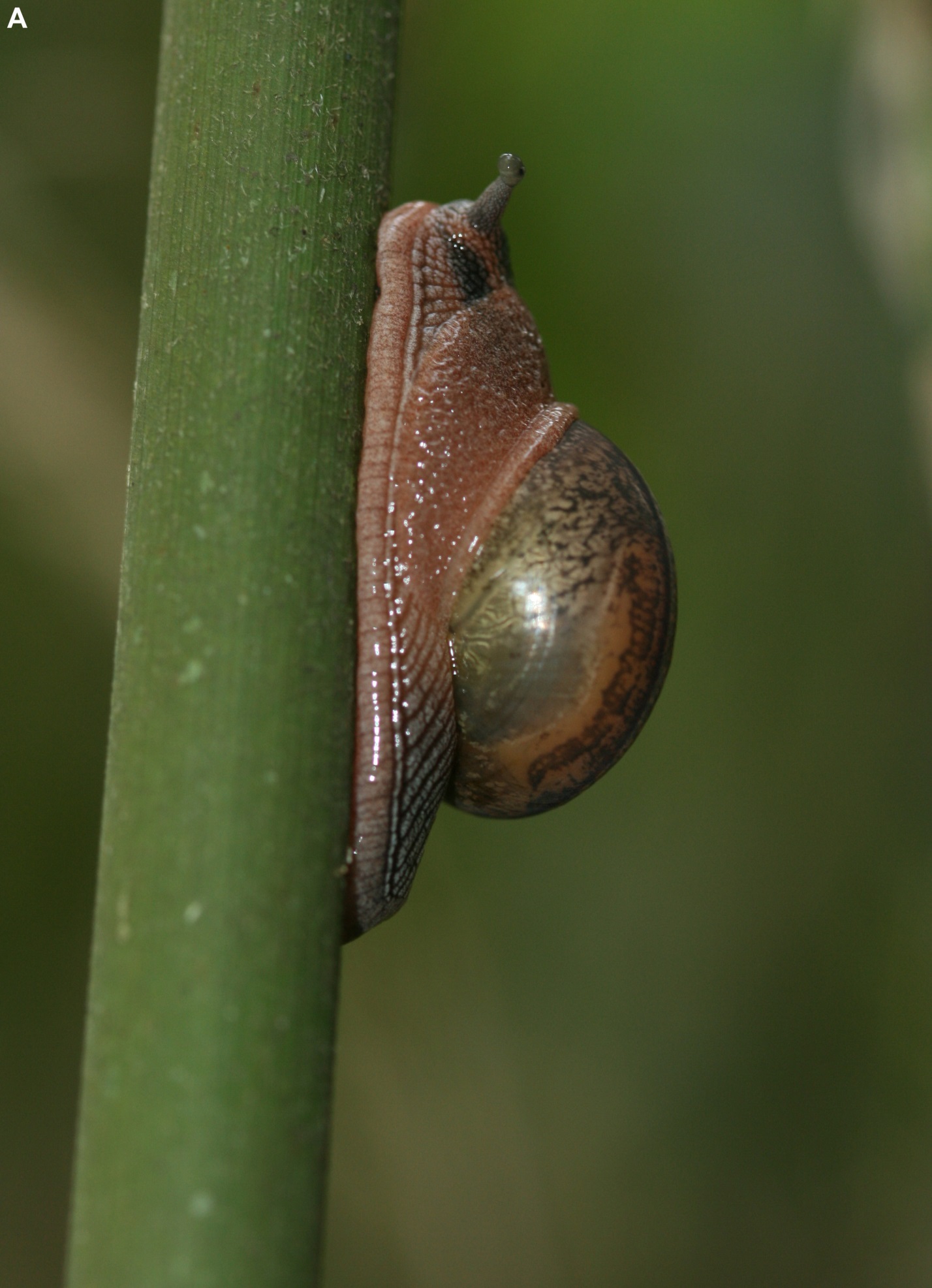

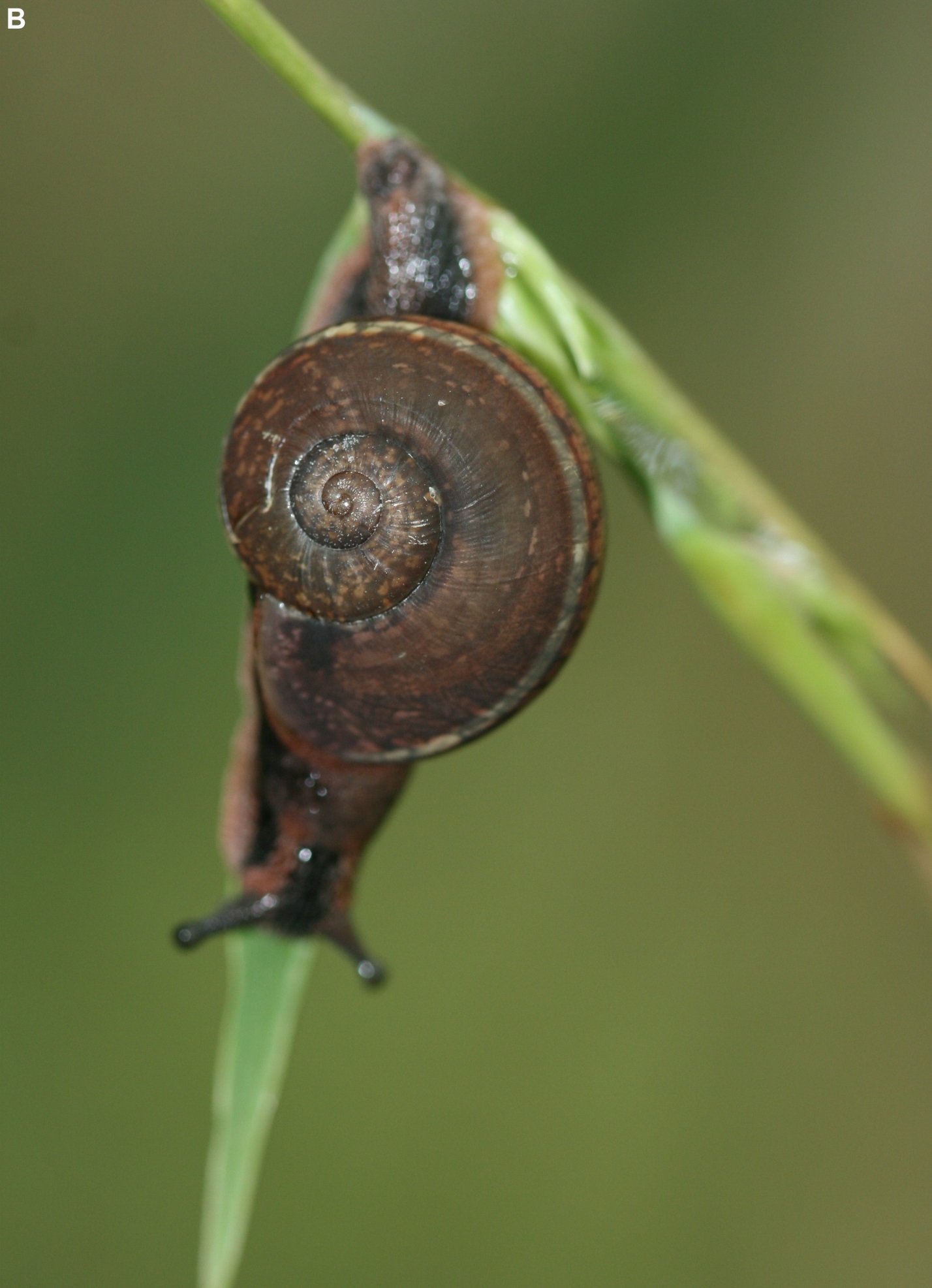

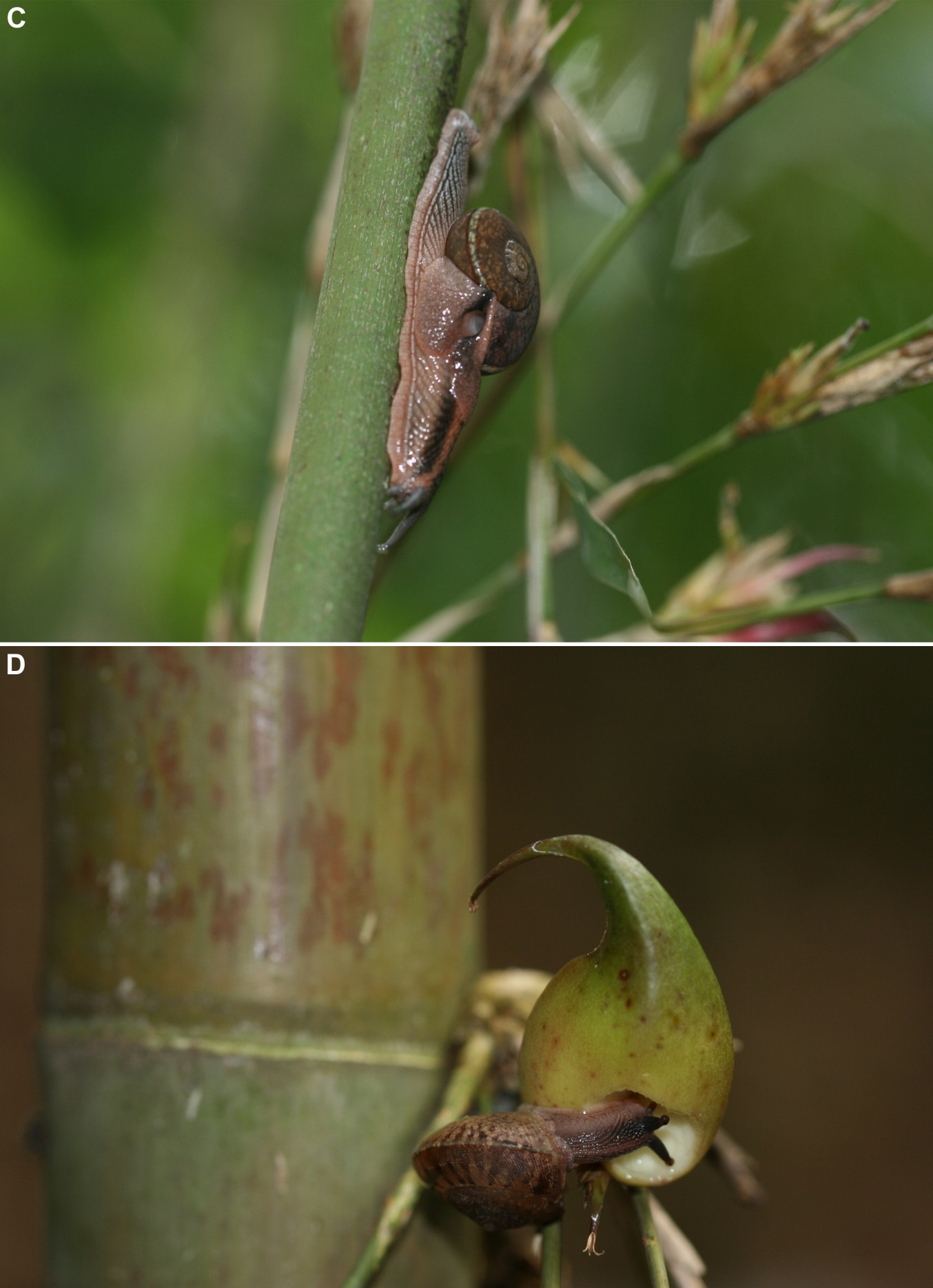


**Fig S10. (A-D).** *M. baccifera*, snail attack, *Macrochlamys* sp. on young fruits.


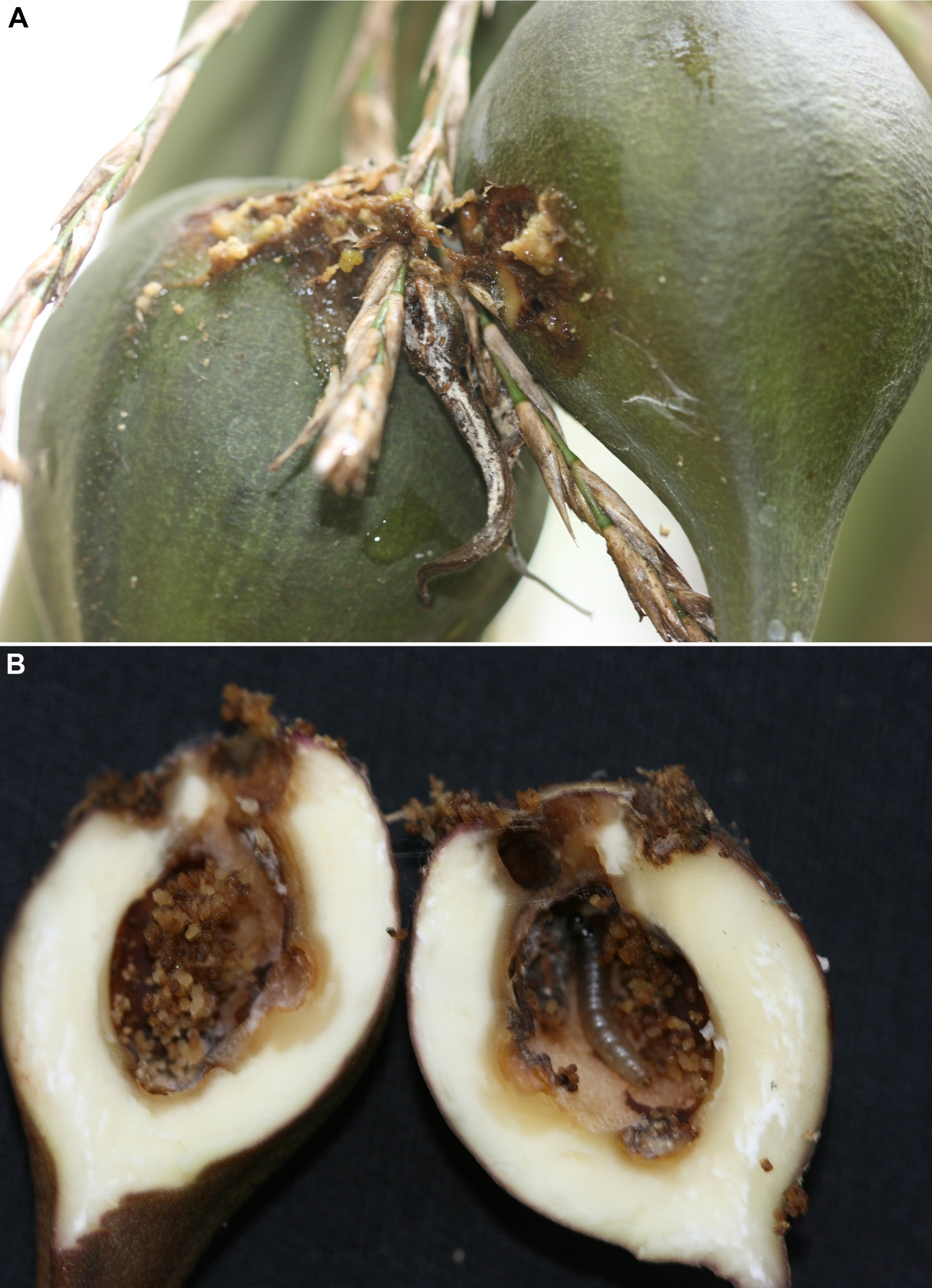

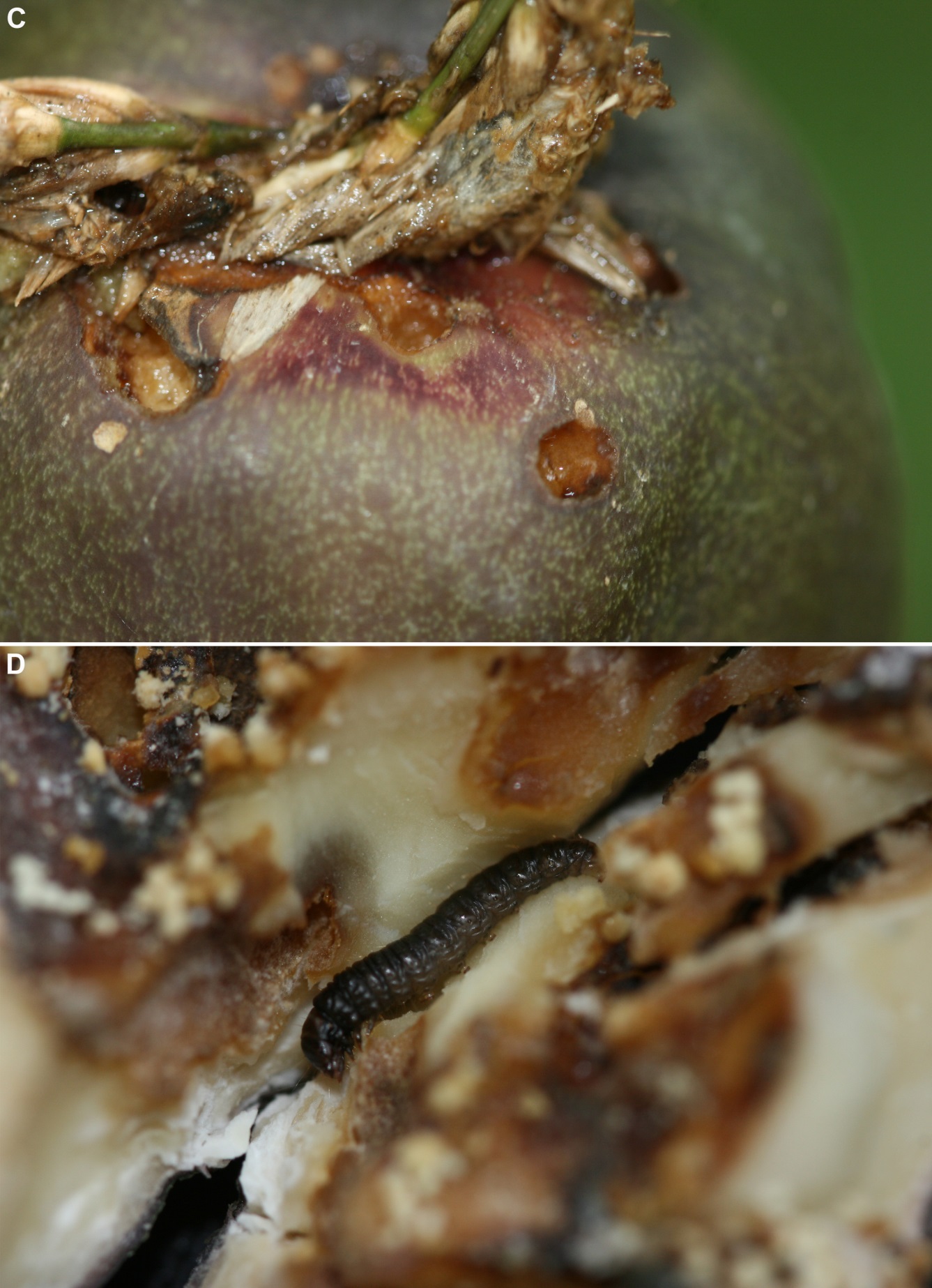


**Fig S11. (A-D).** Larvae of *A. grisella* (lesser wax moth) on *M. baccifera* fruits.

*
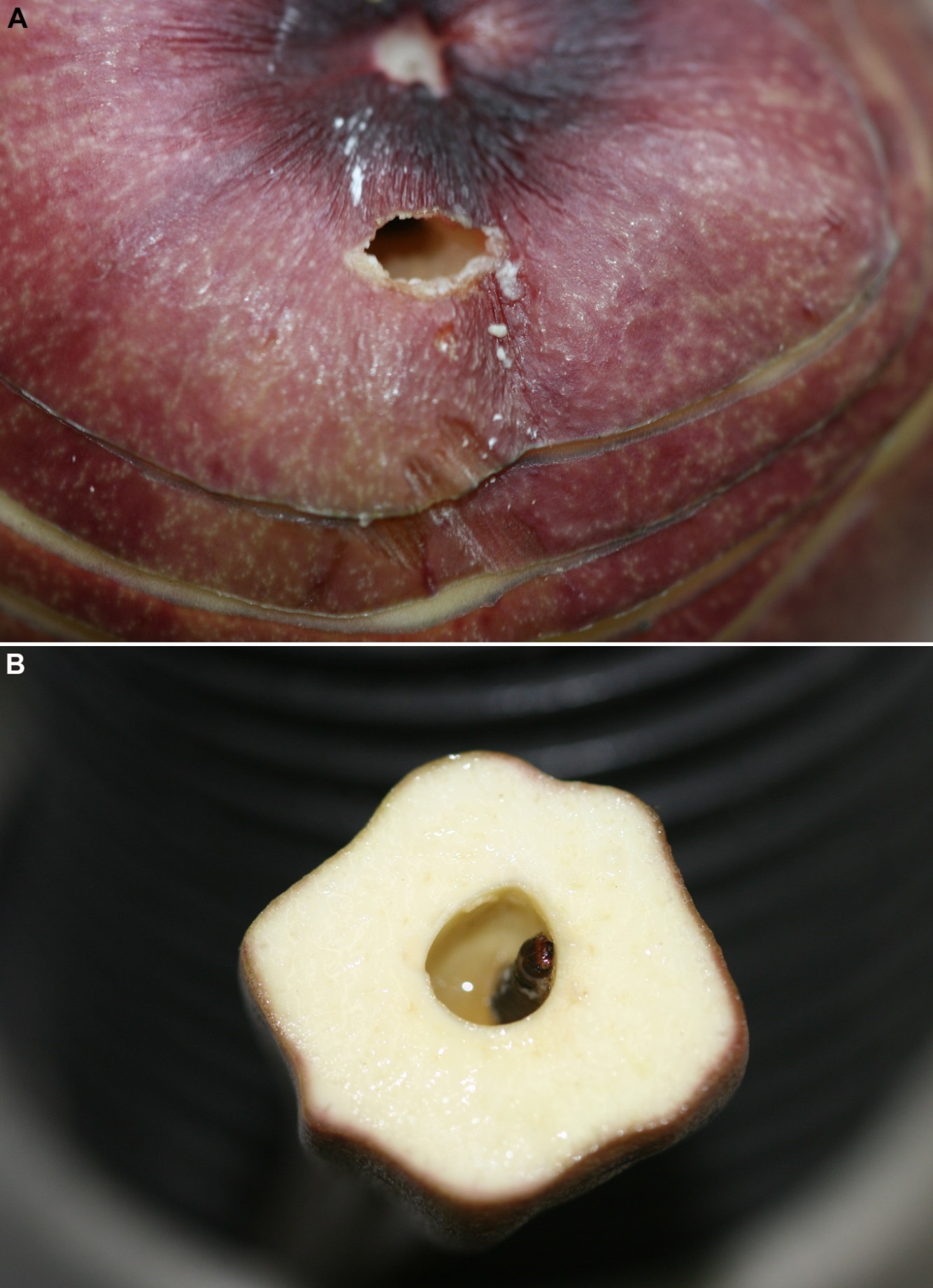
*

**Fig S12. (A-B).** *B. germanica* (German cockroach) larva inside *M. baccifera* fruit.


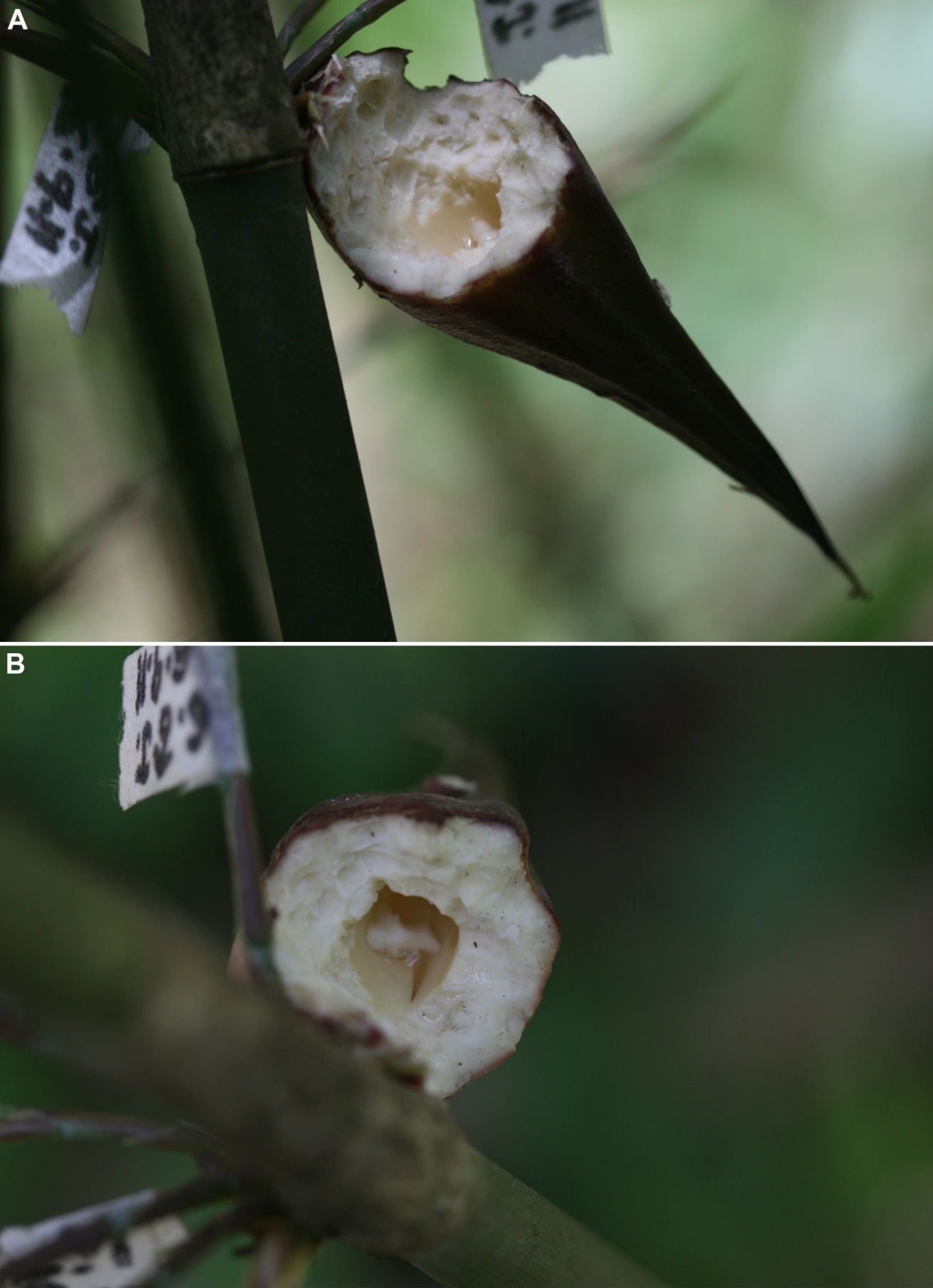


**Fig S13. (A-B).** *R. rattus* predation, *M. baccifera* fruits.


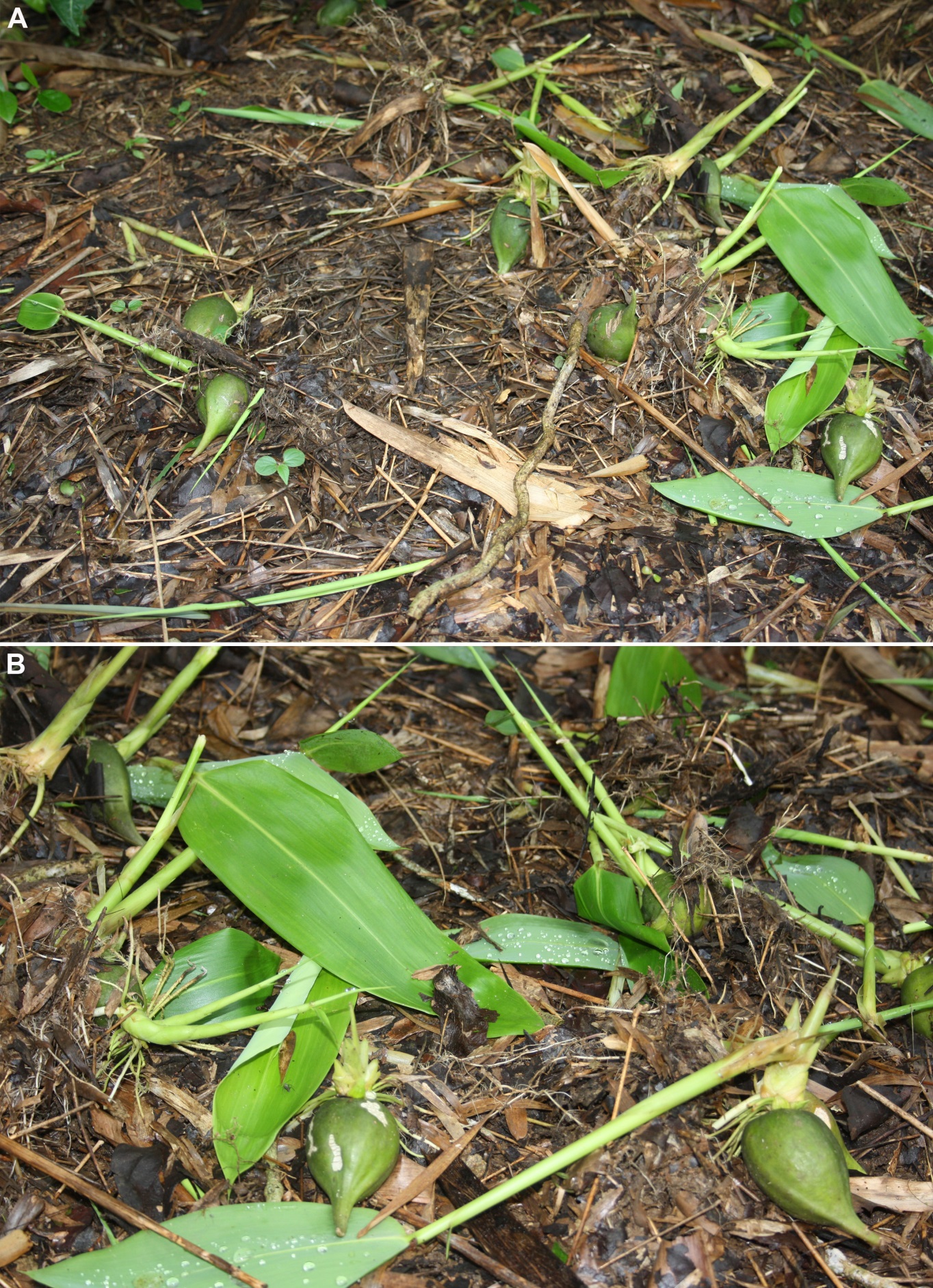


**Fig S14. (A-B).** *M. baccifera* seedling predation, damage by *S. scrofa*.

*
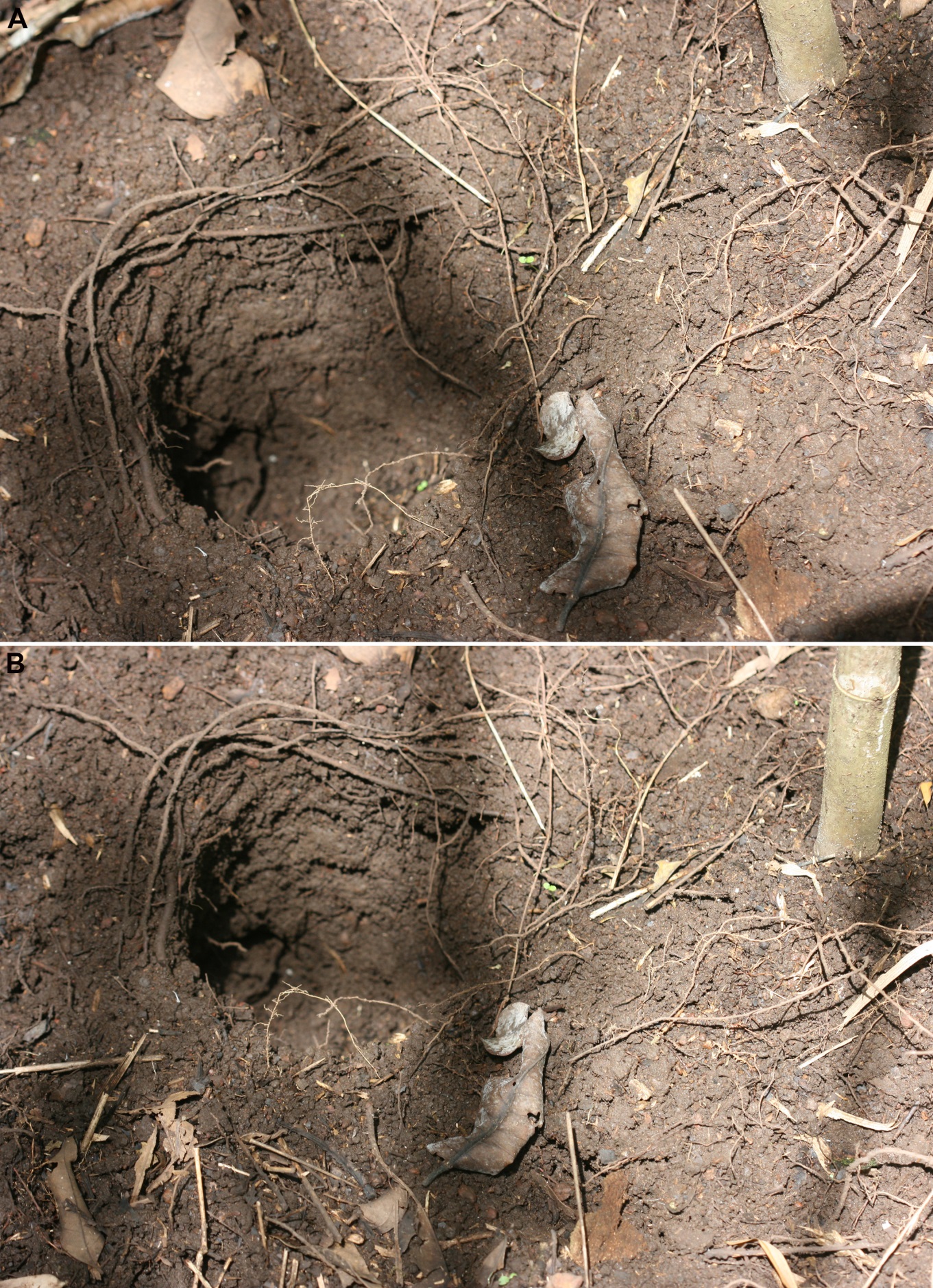
*

**Fig S15. (A-B).**  *S. scrofa*, hoof marks and soil rooting characteristics.


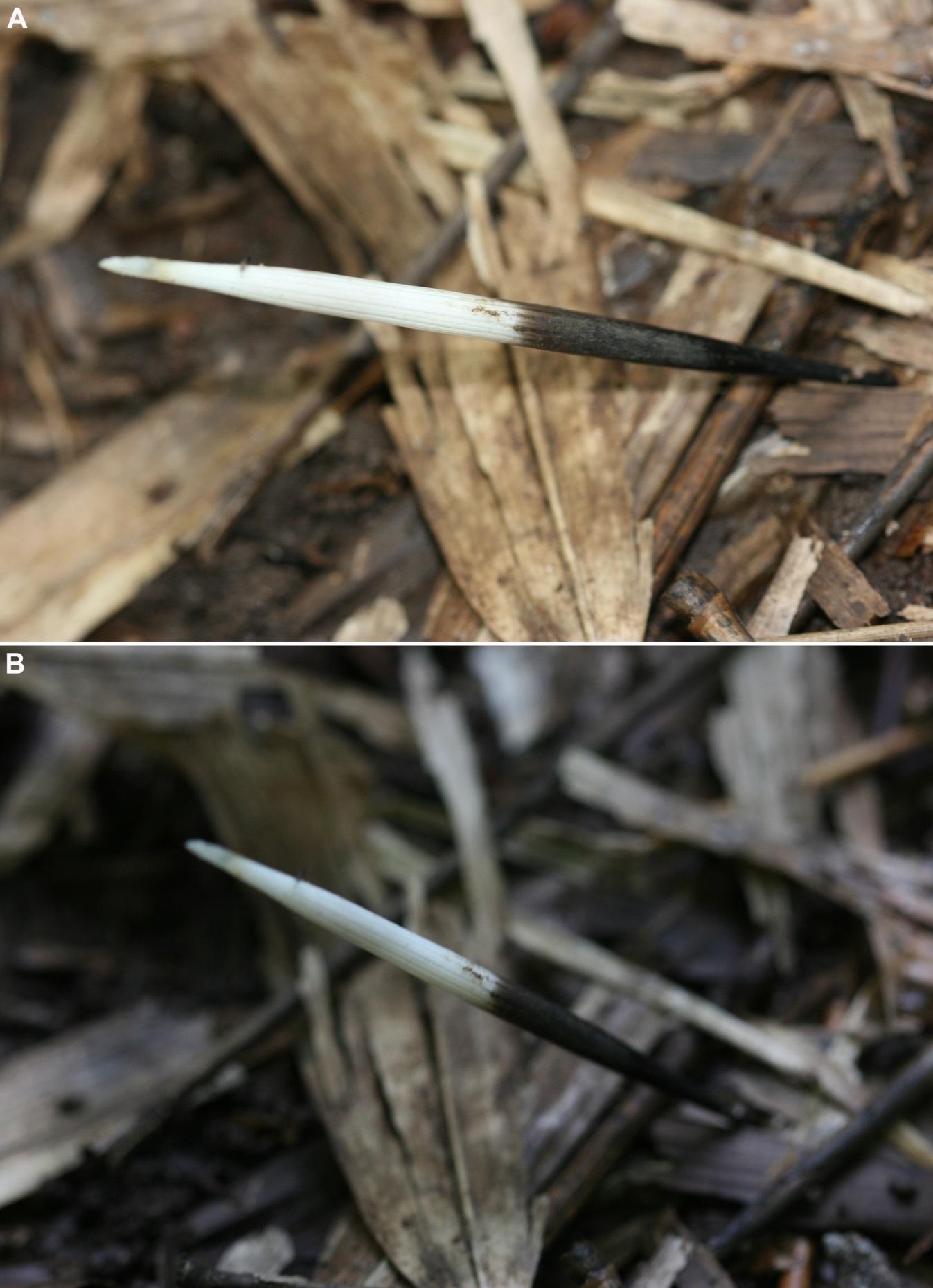


**Fig S16. (A-B).** Quills of porcupine *H. indica*, below *M. baccifera* clump.


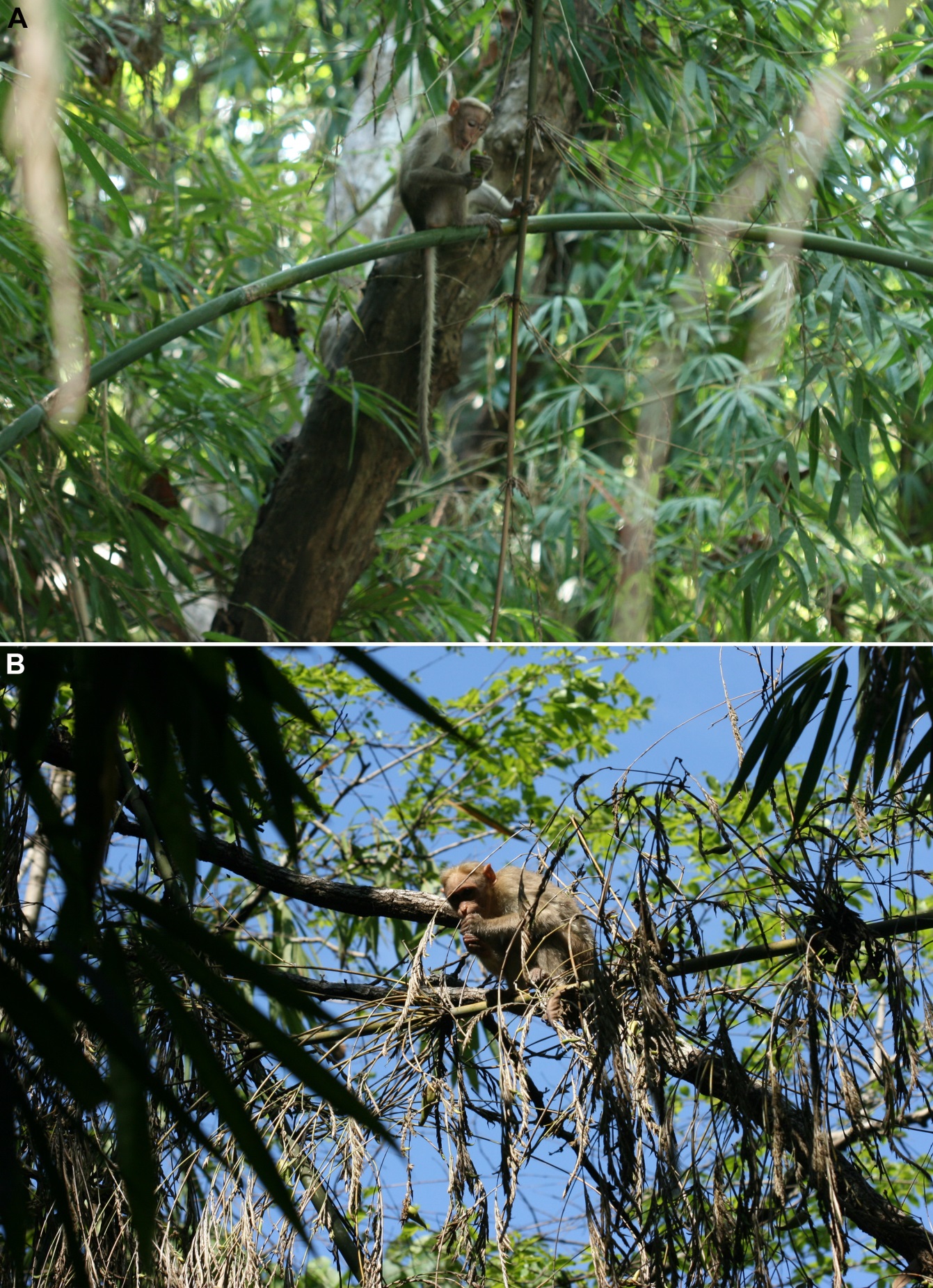


**Fig S17. (A-B).** Bonnet macaque, *M. radiata*, eating *M. baccifera* fruits.


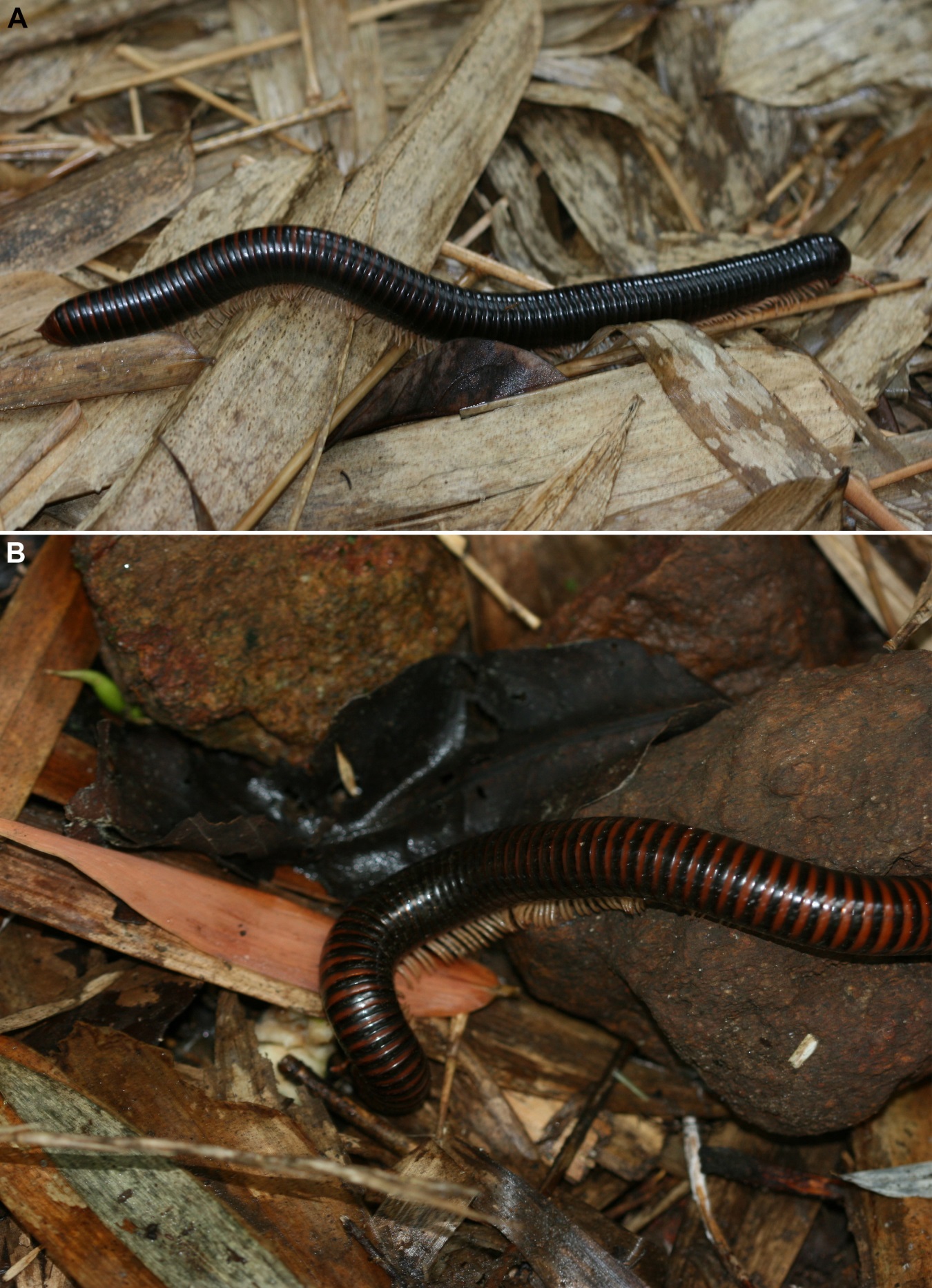


**Fig S18. (A-B).**  Millipede, *S. colosseus*.
